# Supplementary material for: Pre-clinical development of AP4B1 gene replacement therapy for hereditary spastic paraplegia type 47
Source: EMBO Mol Med. 2024 Oct 2;16(11):2882–917. doi: 10.1038/s44321-024-00148-5 (PMC11554807; doi:10.1038/s44321-024-00148-5)
Supplement: Supplementary file 1 — Appendix [file 44321_2024_148_MOESM1_ESM.pdf]

Appendix

Table of contents

Appendix Figure S1 - page 2
Appendix Figure S2 - page 3
Appendix Figure S3 - page 4
Appendix Figure S4 - page 5
Appendix Table S1- page 6 - 22
Appendix Table S2 - page 23 - 35
Appendix Table S3 - page 36
Appendix Table S4 – page 37

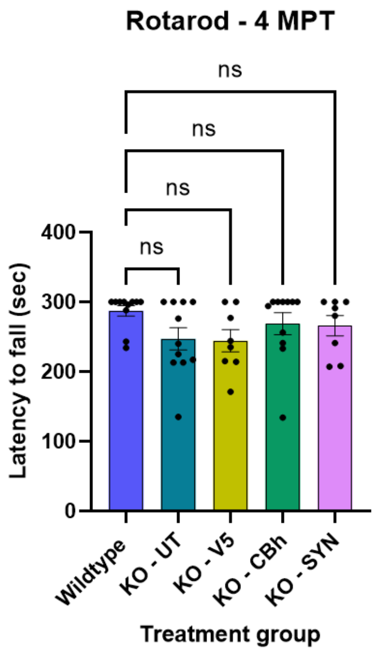

Appendix Figure S1: Rotarod performance at 4 months of age following injection in neonatal demonstrates no significant phenotypic difference between wild type mice and untreated/control treated ap4b1 -/- mice. A trend of reduced latency to fall is observed with untreated and control treated mice and a rescue with treatment. All data groups are males, n ≥ 8 per group. Data are presented as mean ± SEM and are analysed by the Kruskal-Wallis test with Dunn's multiple comparison test.

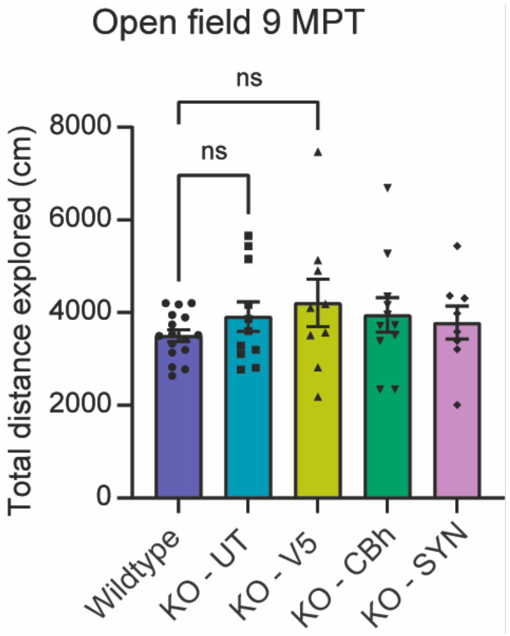

Appendix Figure S2: Open field assessments and 9 months following treatment in neonatal mice demonstrates no significant difference between any of the treatment groups. All data groups are males, n ≥ 8 per group. Data are presented as mean ± SEM and are analysed by a one-way ANOVA with Tukey's multiple comparison test.

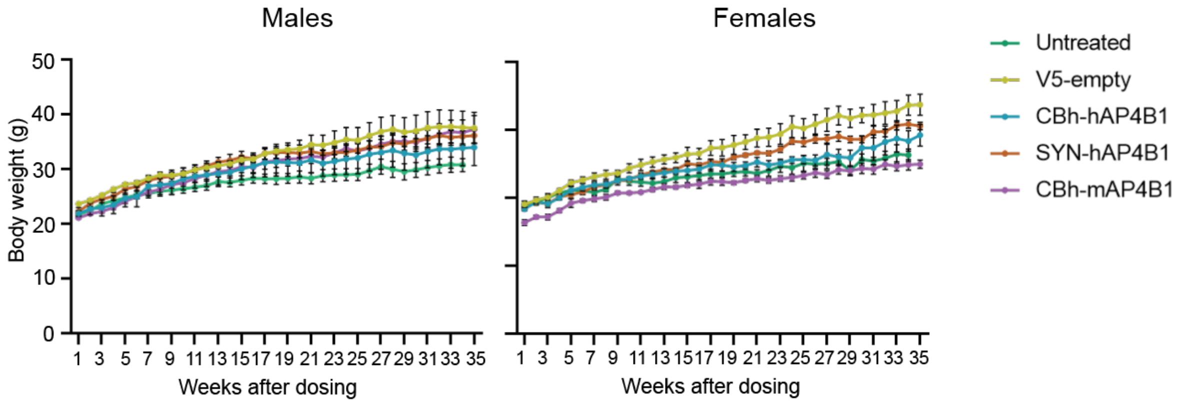

Appendix Figure S3: Male and female weight gain plotted over 35 weeks. Comparison of vectors used in the efficacy and safety studies.

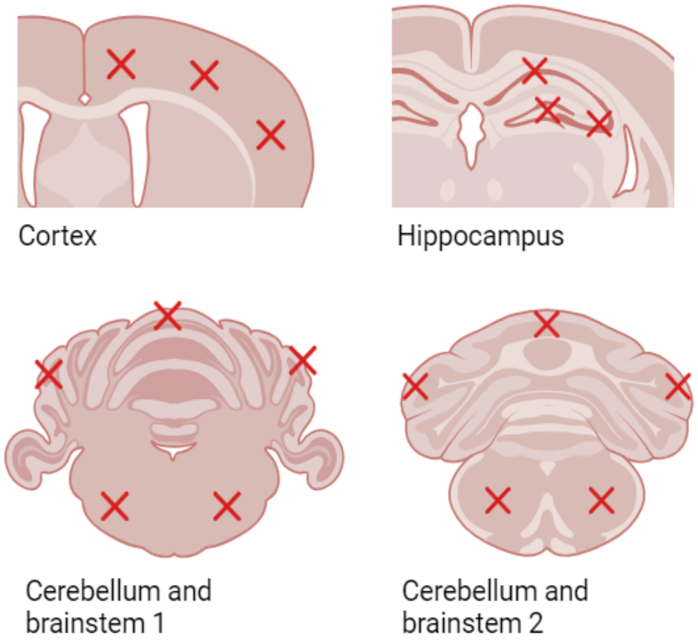

Appendix Figure S4: Immunofluorescent imaging areas within distinct anatomical regions for direct comparison of ATG9A accumulation at the TGN between treatment groups

Appendix Table S1. Tabulated summary of results of a long-term mouse safety study.

This study was designed to demonstrate the long-term safety of AAV9 vectors for AP4B1 gene therapy. A number of constructs expressing AP4B1 cDNAs from non-specific and cell specific promoters were compared by both intra cisterna magna and intravenous routes. The first table summarizes the study design and mortality. The subsequent tables summarize group averages and standard errors for haematology, serum chemistry and liver histopathology.

List of Results

Study design and mortality 7
Weight gain – Female 9
Weight gain - Male 11
Haematology at Day 28 13
Haematology at 1 year 16
Serum Chemistry at Day 28 18
Serum Chemistry at 1-year 18
Liver Histology Day 28 19
Liver Histology at 1 year 21

Study design and mortality								
**Group**	**N**	**Treatment**[1]	**Dose (genome copies/Kg)**	**Volume (µl)**	**Route**[2]	[3]**Sac Day 28**	[4]**Sac day 365**	**Mortality**
1	6F/6M	Untreated	NA	NA	NA	3F/3M	3F/3M	None
2	6F/6M	AAV9/V5	$3X10^{12}$	3ul	ICM	3F/3M	3F/3M	None
3	6F/6M	AAV9/CBh-hAP4B1	$3X10^{12}$	3ul	ICM	3F/3M	3F/3M	1F/ d241[5]<br>1M/ d80[6]
4	6F/6M	AAV9/pSYN-hAP4B1	$3X10^{12}$	3ul	ICM	3F/3M	3F/3M	None
5	6F/6M	AAV9/pAP4B1-hAP4B1	$3X10^{12}$	3ul	ICM	3F/3M	3F/3M	1F/d266[5]
6	6F/6M	AAV9/pMep229-hAP4B1	$3X10^{12}$	3ul	ICM	3F/3M	3F/3M	1F/d241[5]
7	6F/6M	AAV9/CBh-mAP4B1	$3X10^{12}$	3ul	ICM	3F/3M	3F/3M	None
8	8F/8M	AAV9/CBh-hAP4B1	$3X10^{13}$	300ul	IV	0	5F/2M[4]	None
9	8F/8M	AAV9/pSYN-hAP4B1	$3X10^{13}$	300ul	IV	0	5F/2M[4]	None

[1] Mice were treated at age 5 weeks.
[2] ICM = intra-cisterna magna. IV =intravenous. NA – not applicable
[3] Interim sacrifice to assess CBC, serum chemistry, histopathology, gene expression
[4] At terminal sacrifice assessment included CBC, serum chemistry, histopathology
[5] Mice with dermatitis resulting from heterobarbering. Incalcitrant to treatment
[6] One mouse from undetermined cause. Failed to gain weight following injection. Humane sacrifice due to lethargy. No necropsy,

**Weight gain - Female**									
	**1. Untreated**	**2. ICM. AAV9/V5**	**3. ICM. AAV9/Cbh-AP4B1**	**4. ICM. AAV9/SYN-AP4B1**	**5. ICM. AAV9/pAP4-AP4B1**	**6. ICM. AAV9/pMEP-AP4B1**	**7. ICM. AAV9/CBh-mAP4B1**	**8. IV. AAV9/Cbh-AP4B1**	**9. IV. AAV9/SYN-AP4B1**
Week 1	0 ± 0	0 ± 0	0.1 ± 0.05	0.2 ± 0.14	0 ± 0	0 ± 0.05	8.3 ± 0.1	0.1 ± 0.05	0.1 ± 0.08

Weight gain - Female									
	**1. Untreated**	**2. ICM. AAV9/V5**	**3. ICM. AAV9/Cbh-AP4B1**	**4. ICM. AAV9/SYN-AP4B1**	**5. ICM. AAV9/pAP4-AP4B1**	**6. ICM. AAV9/pMEP-AP4B1**	**7. ICM. AAV9/CBh-mAP4B1**	**8. IV. AAV9/Cbh-AP4B1**	**9. IV. AAV9/SYN-AP4B1**
Week 2	0 ± 0.02	0.6 ± 0.12	1.1 ± 0.23	1.1 ± 0.46	1.5 ± 0.31	0.5 ± 0.18	8.3 ± 0.35	0.2 ± 0.11	0.1 ± 0.07
Week 3	0.4 ± 0.24	1 ± 0.24	0.8 ± 0.3	0.8 ± 0.27	1.5 ± 0.44	0.8 ± 0.36	8.3 ± 0.36	1.2 ± 0.28	1.3 ± 0.27
Week 4	1.1 ± 0.19	2 ± 0.28	1.7 ± 0.2	1.9 ± 0.38	2.9 ± 0.25	1.2 ± 0.4	8.3 ± 0.37	2.3 ± 1.27	2 ± 0.6
Week 5	1.8 ± 0.3	3.2 ± 0.1	2.8 ± 0.44	2.2 ± 0.48	3.7 ± 0.14	2.6 ± 0.59	8.3 ± 0.38	2.5 ± 0.4	2.6 ± 0.28
Week 6	2.2 ± 0.23	3.6 ± 0.4	3.2 ± 0.42	2.6 ± 0.38	4.4 ± 0.29	2.8 ± 0.16	8.3 ± 0.25	3.5 ± 0.37	2.6 ± 0.13
Week 7	2.1 ± 0.13	4.1 ± 0.51	3.6 ± 0.31	3.3 ± 0.46	4.7 ± 0.24	3.1 ± 0.41	8.3 ± 0.45	3.1 ± 0.49	2.9 ± 0.36
Week 8	1.9 ± 0.12	4.4 ± 0.6	3.7 ± 0.39	3.6 ± 0.4	4.8 ± 0.26	3.2 ± 0.31	8.3 ± 0.46	3.2 ± 0.25	3.1 ± 0.22
Week 9	2.3 ± 0.33	4.6 ± 0.35	4.2 ± 0.57	4.2 ± 0.26	5 ± 0.3	3.9 ± 0.21	8.3 ± 0.27	3.6 ± 0.4	3.2 ± 0.22
Week 10	3.9 ± 0.57	5.3 ± 0.44	4.5 ± 0.28	4.4 ± 0.14	5.4 ± 0.57	4.3 ± 0.29	8.3 ± 0.23	3.9 ± 0.39	3.6 ± 0.14
Week 11	3.5 ± 0.2	5.8 ± 0.64	4.9 ± 0.29	5.1 ± 0.31	5.9 ± 0.3	4.1 ± 0.27	8.3 ± 0.26	4.7 ± 0.55	4.2 ± 0.91
Week 12	3.4 ± 0.16	6.2 ± 0.55	5.1 ± 0.36	5.5 ± 0.2	6.2 ± 0.22	4.4 ± 0.24	8.3 ± 0.25	4.8 ± 0.34	4.5 ± 0.09
Week 13	3.2 ± 0.17	6.6 ± 0.46	5.4 ± 0.45	5.8 ± 0.24	6.4 ± 0.24	4.8 ± 0.23	8.3 ± 0.28	4.9 ± 0.38	4.6 ± 0.11
Week 14	3.5 ± 0.43	6.9 ± 0.7	5.6 ± 0.43	5.9 ± 0.27	6.4 ± 0.4	5.2 ± 0.55	8.3 ± 0.18	5 ± 0.59	4.7 ± 0.18
Week 15	4 ± 0.28	7.4 ± 0.96	5.7 ± 0.54	6.7 ± 0.3	6.7 ± 0.29	5.4 ± 0.5	8.3 ± 0.32	5.1 ± 0.5	5.4 ± 0.23
Week 16	4.2 ± 0.29	7.5 ± 0.77	5.9 ± 0.45	6.5 ± 0.3	7.2 ± 0.65	5.5 ± 0.32	8.3 ± 0.43	5.3 ± 0.88	5.4 ± 0.33
Week 17	4.4 ± 0.47	8.3 ± 0.99	6.5 ± 0.68	7 ± 0.27	7.7 ± 0.67	5.9 ± 0.3	8.3 ± 0.43	5 ± 0.84	5.1 ± 0.3
Week 18	4.6 ± 0.32	8.3 ± 1.23	6.5 ± 0.75	6.9 ± 0.21	7.7 ± 0.44	6.2 ± 0.56	8.3 ± 0.34	5.8 ± 0.67	5.6 ± 0.28
Week 19	4.6 ± 0.36	8.8 ± 1.36	6.2 ± 0.53	7.7 ± 0.32	7.9 ± 0.49	5.9 ± 0.46	8.3 ± 0.25	5.5 ± 0.58	5.7 ± 0.24
Week 20	4.8 ± 0.4	9.2 ± 1.02	6.4 ± 0.39	8 ± 0.26	8.6 ± 0.86	6 ± 0.45	8.3 ± 0.3	5.9 ± 0.63	5.7 ± 0.35
Week 21	4.9 ± 0.35	9.7 ± 1.2	6.9 ± 0.62	8.3 ± 0.2	9.2 ± 0.9	6.3 ± 0.35	8.3 ± 0.21	6.8 ± 0.52	6.1 ± 0.48
Week 22	4.7 ± 0.32	9.9 ± 1.3	6.5 ± 0.53	8.3 ± 0.36	8.3 ± 0.79	6.6 ± 0.55	8.3 ± 0.35	6.7 ± 0.61	6.2 ± 0.47
Week 23	5.1 ± 0.59	10.3 ± 1.39	6.8 ± 0.49	8.9 ± 0.42	9.2 ± 0.93	6.8 ± 0.51	8.3 ± 0.19	6.6 ± 0.47	6.2 ± 0.51
Week 24	5.7 ± 0.48	11.4 ± 1.53	7.3 ± 0.35	10 ± 0.39	9.9 ± 1.36	7.4 ± 0.54	8.3 ± 0.47	7.4 ± 0.62	6.7 ± 0.58

Weight gain - Female									
	**1. Untreated**	**2. ICM. AAV9/V5**	**3. ICM. AAV9/Cbh-AP4B1**	**4. ICM. AAV9/SYN-AP4B1**	**5. ICM. AAV9/pAP4-AP4B1**	**6. ICM. AAV9/pMEP-AP4B1**	**7. ICM. AAV9/CBh-mAP4B1**	**8. IV. AAV9/Cbh-AP4B1**	**9. IV. AAV9/SYN-AP4B1**
Week 25	5.5 ± 0.77	11.2 ± 1.35	7.3 ± 0.52	9.9 ± 0.6	9.7 ± 1.27	7.3 ± 0.7	8.3 ± 0.3	7.3 ± 1.03	6.9 ± 0.58
Week 26	6.2 ± 0.79	11.8 ± 1.32	7.2 ± 0.66	10.3 ± 0.53	10.5 ± 1.64	8.1 ± 0.82	8.3 ± 0.47	7.4 ± 0.94	7.5 ± 0.94
Week 27	6 ± 0.44	12.4 ± 1.5	8 ± 1.41	10.4 ± 0.59	10.5 ± 1.74	8 ± 0.81	8.3 ± 0.55	8.1 ± 0.89	8.3 ± 1.06
Week 28	6.1 ± 0.43	13 ± 1.29	7.7 ± 1.03	10.7 ± 0.67	10.8 ± 1.61	8.3 ± 0.88	8.3 ± 0.46	8.1 ± 0.98	8.3 ± 0.73
Week 29	6.4 ± 0.95	12.6 ± 1.26	7.6 ± 1.06	10.3 ± 0.45	10.1 ± 1.6	8.2 ± 0.77	8.3 ± 0.56	8 ± 1.05	8.9 ± 0.55
Week 30	5.1 ± 0.2	13.1 ± 1.13	9.2 ± 1.3	10.3 ± 0.45	10.6 ± 1.63	8.8 ± 1.01	8.3 ± 0.68	8.1 ± 1.11	8.1 ± 0.98
Week 31	6.7 ± 0.54	13.1 ± 1.44	9.3 ± 1.39	11.4 ± 0.78	10.8 ± 1.75	8.9 ± 1.13	8.3 ± 0.69	8.3 ± 0.86	7.6 ± 1.02
Week32	6.5 ± 0.17	13.4 ± 1.52	10.1 ± 1.41	11.5 ± 0.72	11.8 ± 1.88	9.2 ± 0.88	8.7 ± 0.66	8.6 ± 1.03	8.2 ± 0.87
Week 33	6.9 ± 0.58	13.7 ± 1.44	10.6 ± 1.47	12.4 ± 0.63	12.5 ± 2.24	9.2 ± 1.28	8.7 ± 0.63	9.4 ± 1.03	8.9 ± 0.94
Week 34	7.5 ± 0.95	14.6 ± 1.32	10.3 ± 1.11	12.5 ± 0.57	12.9 ± 2.32	9.7 ± 1.1	8.7 ± 0.64	10.3 ± 1.24	9.3 ± 1.1
Week 35	7.4 ± 0.47	14.6 ± 1.44	11.1 ± 1.15	12.3 ± 0.48	12.9 ± 2.4	9.9 ± 1.51	8.7 ± 0.54	10.1 ± 1.21	9.2 ± 1.14
Week 36	8.2 ± 0.63	14.9 ± 1.34	11.1 ± 1.41	13.5 ± 0.48	13.4 ± 2.39	9.8 ± 1.15	8.7 ± 0.73	10.6 ± 1.46	9.4 ± 1.11
Week 37	7.1 ± 0.53	14.2 ± 1.71	11.1 ± 1.08	12.5 ± 0.43	13 ± 2.27	9.7 ± 1.02	8.7 ± 0.63	11.2 ± 1.5	9.8 ± 1.03
Week 38	8 ± 0.74	14.6 ± 1.51	11.8 ± 1.17	12.9 ± 0.48	13.2 ± 2.11	10.4 ± 1.05	8.7 ± 0.35	9.5 ± 1.25	9.4 ± 1.52

Weight gain - Female									
	1. Untreated	2. ICM. AAV9/V5	3. ICM. AAV9/Cbh-AP4B1	4. ICM. AAV9/SYN-AP4B1	5. ICM. AAV9/pAP4-AP4B1	6. ICM. AAV9/pMEP-AP4B1	7. ICM. AAV9/CBh-mAP4B1	8. IV. AAV9/Cbh-AP4B1	9. IV. AAV9/SYN-AP4B1
Week 39	9.2 ± 1.02	15.5 ± 1.51	12.2 ± 1.34	12.7 ± 0.6	14 ± 2.49	10.3 ± 1.46	8.7 ± 0.62	10 ± 1.37	9.6 ± 1.26
Week 40	8.3 ± 0.8	14.9 ± 0.72	12.9 ± 1.53	13.8 ± 0.65	13.7 ± 2.59	10.2 ± 1.27	8.7 ± 0.8	10.3 ± 1.44	10.1 ± 1.31
Week 41	8.6 ± 0.81	16.2 ± 0.95	12.9 ± 2.02	14.3 ± 0.76	14.1 ± 2.81	11.7 ± 1.32	8.7 ± 0.91	11.1 ± 1.36	10.2 ± 1.25
Week 42	9.4 ± 0.78	16.1 ± 1.45	13.8 ± 1.53	13.7 ± 1.08	14 ± 2.77	11.4 ± 1.11	8.7 ± 0.89	10.7 ± 1.36	10.4 ± 1.39
Week 43	9.7 ± 0.71	16.6 ± 1.34	14.8 ± 1.62	14 ± 1.39	14.1 ± 2.82	11.3 ± 0.94	8.7 ± 0.96	11 ± 1.59	10.4 ± 1.23
Week 44	9.9 ± 1.01	16.5 ± 1.3	14.1 ± 1.53	13.9 ± 1.3	13.8 ± 3.05	11.5 ± 0.91	8.7 ± 1.04	11.4 ± 1.3	10.4 ± 1.02
Week 45	10.2 ± 0.55	17 ± 1.39	14.6 ± 1.53	14.1 ± 1.46	14.3 ± 3.3	11.8 ± 1.25	8.7 ± 1.16	11.3 ± 1.61	10.4 ± 1.17
Week 46	9.5 ± 0.69	16.9 ± 1.44	14.8 ± 1.91	13.9 ± 1.07	14 ± 3.15	11.3 ± 1.5	8.7 ± 1.12	11.8 ± 1.85	10.4 ± 1.26
Week 47	9.8 ± 0.58	16.8 ± 1.43	14.8 ± 1.69	13.9 ± 1.1	13.6 ± 3.03	11.4 ± 1.12	8.7 ± 1.2	10.7 ± 1.62	10.1 ± 1.21

Weight gain - Male									
	1. Untreated	2. ICM. AAV9/V5	3. ICM. AAV9/Cbh-AP4B1	4. ICM. AAV9/SYN-AP4B1	5. ICM. AAV9/pAP4-AP4B1	6. ICM. AAV9/pMEP-AP4B1	7. ICM. AAV9/CBh-mAP4B1	8. IV. AAV9/Cbh-AP4B1	9. IV. AAV9/SYN-AP4B1
Week 1	0 ± 0	0 ± 0	0.9 ± 0.64	0 ± 0	1.5 ± 1.26	0 ± 0	0 ± 0	0.2 ± 0	0 ± 0
Week 2	0 ± 0	0.7 ± 0.11	1.7 ± 0.75	1.6 ± 0.3	0.5 ± 0.32	0.7 ± 0.17	0.7 ± 0.12	0 ± 0	0.5 ± 0
Week 3	0.8 ± 0.31	1.6 ± 0.29	1.9 ± 1.14	2.3 ± 0.57	3.1 ± 1.49	1.2 ± 0.13	1.2 ± 0.24	1.7 ± 0	2.1 ± 0
Week 4	2 ± 0.27	2.7 ± 0.06	2.5 ± 1.27	2.8 ± 0.37	3.8 ± 1.35	1.9 ± 0.41	1.9 ± 0.43	2.2 ± 0	2.8 ± 0
Week 5	2.6 ± 0.21	3.5 ± 0.2	3.7 ± 1.21	4.2 ± 0.37	4.8 ± 1.28	2.7 ± 0.27	3 ± 0.57	3.4 ± 0	3.9 ± 0

Weight gain - Male									
	1. Untreated	2. ICM. AAV9/V5	3. ICM. AAV9/Cbh-AP4B1	4. ICM. AAV9/SYN-AP4B1	5. ICM. AAV9/pAP4-AP4B1	6. ICM. AAV9/pMEP-AP4B1	7. ICM. AAV9/CBh-mAP4B1	8. IV. AAV9/Cbh-AP4B1	9. IV. AAV9/SYN-AP4B1
Week 6	3.1 ± 0.35	3.8 ± 0.2	3.9 ± 1.46	4.6 ± 0.33	4.9 ± 1.37	3.4 ± 0.19	3.8 ± 0.5	3.5 ± 0	4.8 ± 0
Week 7	3.7 ± 0.27	4.7 ± 0.33	5.6 ± 1.19	5.7 ± 0.19	6 ± 1.28	4.1 ± 0.35	4.7 ± 0.56	4.1 ± 0	4.8 ± 0
Week 8	3.9 ± 0.39	5.2 ± 0.35	5.8 ± 1.53	6.2 ± 0.3	6.9 ± 1.22	4.5 ± 0.49	5.2 ± 0.62	4.9 ± 0	5.8 ± 0
Week 9	4.3 ± 0.3	5.2 ± 0.44	6.1 ± 1.42	6.5 ± 0.38	7.3 ± 1.22	4.9 ± 0.58	6 ± 0.76	5.3 ± 0	6.4 ± 0
Week 10	4.5 ± 0.48	5.5 ± 0.34	6.9 ± 1.41	7 ± 0.45	7.7 ± 1.05	5.3 ± 0.41	6.4 ± 0.71	5.9 ± 0	6.6 ± 0
Week 11	4.8 ± 0.47	6.2 ± 0.26	7.4 ± 1.48	7.6 ± 0.4	8.4 ± 1.06	5.9 ± 0.67	7.2 ± 0.75	6.1 ± 0	6.7 ± 0
Week 12	5.1 ± 0.45	6.6 ± 0.28	7.6 ± 1.47	8.3 ± 0.38	8.7 ± 1.09	6.4 ± 0.55	7.8 ± 0.88	6.9 ± 0	7.4 ± 0
Week 13	5.4 ± 0.44	7 ± 0.44	7.9 ± 1.45	9.1 ± 0.4	9 ± 1.13	6.9 ± 0.45	8.4 ± 1.02	7.7 ± 0	7.7 ± 0
Week 14	6.1 ± 0.49	7.3 ± 0.47	8.1 ± 1.27	9.4 ± 0.43	9.7 ± 1.06	7 ± 0.69	8.6 ± 1.05	8.5 ± 0	8 ± 0
Week 15	6 ± 0.54	8.1 ± 0.68	8.8 ± 1.35	10 ± 0.45	10.1 ± 0.95	8.2 ± 0.66	9.3 ± 1.14	8.6 ± 0	8.8 ± 0
Week 16	6.3 ± 0.53	8.2 ± 0.62	9.3 ± 1.44	9.7 ± 0.39	10.4 ± 1.1	8.3 ± 0.85	9.2 ± 1.07	8.6 ± 0	9.3 ± 0
Week 17	6.7 ± 0.6	9.2 ± 0.94	9.9 ± 1.87	10.7 ± 0.45	11.2 ± 1.07	8.9 ± 0.92	10.2 ± 1.4	9.2 ± 0	9.5 ± 0
Week 18	6.6 ± 0.59	9.6 ± 1.23	10 ± 1.82	10.8 ± 0.6	11.2 ± 0.91	9.3 ± 1.14	10.4 ± 1.27	9.4 ± 0	9.5 ± 0
Week 19	6.6 ± 0.8	9.9 ± 1.23	9.9 ± 1.82	10.8 ± 0.81	11.6 ± 1.08	8.7 ± 1.08	10.7 ± 1.31	9.6 ± 0	9.6 ± 0
Week 20	6.7 ± 0.69	10 ± 1.61	9.8 ± 1.77	10.7 ± 0.96	11.3 ± 0.98	8.8 ± 1.04	10.8 ± 1.26	4.7 ± 0	10.4 ± 0
Week 21	6.9 ± 0.85	10.8 ± 1.77	10.4 ± 1.63	11 ± 0.99	11.6 ± 1.07	9.3 ± 1.08	11.2 ± 1.35	9.3 ± 0	9.5 ± 0
Week 22	6.7 ± 0.77	10.7 ± 1.83	9.8 ± 1.7	10.6 ± 0.88	11.6 ± 0.96	9.2 ± 1.19	11.1 ± 1.35	9.8 ± 0	9.3 ± 0
Week 23	7.2 ± 0.9	8.7 ± 3.58	10.1 ± 1.74	10.7 ± 0.57	11.5 ± 0.84	9.6 ± 1.28	11.9 ± 1.31	9.9 ± 0	9.5 ± 0
Week 24	7.3 ± 0.86	11.7 ± 2.2	10.5 ± 2.07	10.9 ± 0.9	12.1 ± 1.18	10 ± 1.24	12.6 ± 1.41	10.3 ± 0	10.1 ± 0
Week 25	7.4 ± 0.86	11.6 ± 2.32	10.7 ± 2.13	11.3 ± 0.94	12 ± 1.17	10 ± 1.46	12.2 ± 1.42	10.2 ± 0	9.9 ± 0
Week 26	7.4 ± 0.77	12.5 ± 2.34	11.4 ± 2.3	11.8 ± 0.82	12.4 ± 1.07	11 ± 1.45	12.7 ± 1.39	10.4 ± 0	9.8 ± 0
Week 27	8.1 ± 0.96	13.2 ± 2.67	11.7 ± 2.31	11.9 ± 0.83	12.9 ± 0.91	11 ± 1.32	13.4 ± 1.51	11.2 ± 0	10.7 ± 0

Weight gain - Male									
	**1. Untreated**	**2. ICM. AAV9/V5**	**3. ICM. AAV9/Cbh-AP4B1**	**4. ICM. AAV9/SYN-AP4B1**	**5. ICM. AAV9/pAP4-AP4B1**	**6. ICM. AAV9/pMEP-AP4B1**	**7. ICM. AAV9/CBh-mAP4B1**	**8. IV. AAV9/Cbh-AP4B1**	**9. IV. AAV9/SYN-AP4B1**
Week 28	8.8 ± 1.18	13.6 ± 2.47	12.1 ± 2.29	12.6 ± 0.82	13.6 ± 0.69	11.2 ± 1.27	13.9 ± 1.64	11.8 ± 0	11 ± 0
Week 29	8.3 ± 1.29	13.1 ± 2.64	11.6 ± 2.27	12.4 ± 0.97	13.4 ± 0.73	10.7 ± 1.29	13.9 ± 1.61	11.6 ± 0	11.8 ± 0
Week 30	7.9 ± 1.28	13.3 ± 2.87	11.3 ± 2.22	13 ± 1.01	13.5 ± 0.66	10.7 ± 1.56	13.8 ± 1.72	11.4 ± 0	11.3 ± 0
Week 31	8.2 ± 1.2	13.9 ± 2.91	11.9 ± 2.17	13.4 ± 1.09	13.9 ± 0.66	11.2 ± 1.6	14.5 ± 1.93	10.9 ± 0	11 ± 0
Week 32	8.6 ± 1.14	14 ± 3.1	4.2 ± 8.3	13.9 ± 1.08	14 ± 0.74	11.6 ± 1.47	15.2 ± 2.03	11.3 ± 0	5.8 ± 0
Week 33	9 ± 1.09	14.1 ± 2.96	4.2 ± 8.4	13.6 ± 1.22	13.7 ± 0.75	11.5 ± 1.76	15.7 ± 2.1	12.9 ± 0	12.3 ± 0
Week 34	9.2 ± 1.18	13.9 ± 2.97	4.4 ± 8.4	13.7 ± 1.43	13.8 ± 0.88	12 ± 1.71	15.6 ± 2.31	12.4 ± 0	11.9 ± 0
Week 35	9.1 ± 0.97	13.8 ± 2.83	4.5 ± 8.44	13.9 ± 1.24	14.2 ± 0.81	12.1 ± 1.7	16.1 ± 2.56	12.2 ± 0	12.2 ± 0
Week 36	9.7 ± 1.05	14.1 ± 2.77	4.4 ± 8.46	14.2 ± 1.26	13.9 ± 0.98	11.7 ± 1.9	16.5 ± 2.82	12.4 ± 0	12.4 ± 0
Week 37	9.9 ± 1.14	14.4 ± 2.84	4.3 ± 8.46	13.9 ± 1.22	13.6 ± 1.1	11.6 ± 1.68	16.3 ± 2.87	11.7 ± 0	12.4 ± 0
Week 38	10.9 ± 1.11	15.1 ± 2.94	4.4 ± 8.5	14.5 ± 1.19	13.9 ± 1.1	12.3 ± 1.72	16.8 ± 3	11.5 ± 0	10.5 ± 0
Week 39	9.3 ± 1.08	15.6 ± 2.99	4.5 ± 8.5	15.1 ± 1.45	14.5 ± 1.3	13 ± 1.77	17.6 ± 2.94	11.5 ± 0	11.9 ± 0
Week 40	9.4 ± 1.22	15.2 ± 3.12	4.8 ± 8.6	14.6 ± 1.42	14.3 ± 1.09	13.2 ± 1.97	17.3 ± 3.14	12.4 ± 0	13 ± 0
Week 41	10 ± 1.33	15.5 ± 3.04	5 ± 8.65	15.4 ± 1.35	14.8 ± 1.05	13.3 ± 1.92	16.1 ± 2.33	12.3 ± 0	13 ± 0

Weight gain - Male									
	1. Untreated	2. ICM. AAV9/V5	3. ICM. AAV9/Cbh-AP4B1	4. ICM. AAV9/SYN-AP4B1	5. ICM. AAV9/pAP4-AP4B1	6. ICM. AAV9/pMEP-AP4B1	7. ICM. AAV9/CBh-mAP4B1	8. IV. AAV9/Cbh-AP4B1	9. IV. AAV9/SYN-AP4B1
Week 42	9.9 ± 1.21	15.8 ± 3.14	5.4 ± 8.8	15.5 ± 1.34	15.4 ± 1.19	13.5 ± 1.94	16.3 ± 2.76	12.2 ± 0	13.3 ± 0
Week 43	9.2 ± 1.48	16.2 ± 3.44	5.5 ± 8.86	15.6 ± 1.65	15.4 ± 1.21	13.2 ± 2	16.4 ± 2.63	11.6 ± 0	13.7 ± 0
Week 44	9.3 ± 1.1	16.4 ± 3.65	5.3 ± 8.86	15.3 ± 1.37	15.7 ± 1.29	13.4 ± 2.01	16 ± 2.26	12.2 ± 0	13.4 ± 0
Week 45	10 ± 1.08	16.7 ± 3.65	5.6 ± 8.91	15.7 ± 1.07	15.5 ± 1.12	14 ± 2.06	16.2 ± 2.42	11.3 ± 0	12.8 ± 0
Week 46	9.8 ± 1.01	16.5 ± 3.64	5 ± 8.73	15.5 ± 1.27	15.5 ± 1.25	13.5 ± 2.48	16.3 ± 2.69	11.6 ± 0	13 ± 0
Week 47	9.6 ± 1.27	16.4 ± 3.4	5.1 ± 8.72	15.6 ± 1.22	15 ± 1.14	13.6 ± 2.28	15.9 ± 2.99	11.5 ± 0	13 ± 0

Hematology at Day 28							
	1. Untreated	2. ICM. V5	3. ICM. CBh-hAP4B1	4. ICM. SYN-hAP4B1	5. ICM. AP4B1-AP4B1	6. ICM. MEP-hAP4B1	7. ICM. Cbh-mAP4B1
WBC x10^3/uL	5.2 ± 0.39	5.8 ± 0.7	6.4 ± 0.41	6.7 ± 0.85	6.5 ± 0.51	6.1 ± 0.59	6.1 ± 0.68
RBC x10^6/uL	10.5 ± 0.24	10.3 ± 0.15	10.3 ± 0.25	10.5 ± 0.42	10.4 ± 0.33	10.3 ± 0.06	10.3 ± 0.27
HGB g/dL	15.4 ± 0.33	15.1 ± 0.26	14.9 ± 0.33	15.2 ± 0.53	15 ± 0.41	14.9 ± 0.14	14.9 ± 0.27
HCT %	51.1 ± 1.09	50.4 ± 0.85	50.1 ± 1.24	50.9 ± 1.98	50.4 ± 1.6	49.8 ± 0.36	50 ± 1.09
MCV fL	48.6 ± 0.24	48.7 ± 0.29	48.5 ± 0.53	48.5 ± 0.25	48.5 ± 0.13	48.4 ± 0.37	48.7 ± 0.65
MCH pg	14.6 ± 0.06	14.6 ± 0.09	14.5 ± 0.05	14.4 ± 0.13	14.4 ± 0.07	14.5 ± 0.11	14.5 ± 0.22
MCHC g/dL	30 ± 0.19	29.9 ± 0.03	29.8 ± 0.31	29.8 ± 0.16	29.7 ± 0.15	29.8 ± 0.2	29.8 ± 0.17
RDW %	17 ± 0.15	17 ± 0.17	17 ± 0.3	17.1 ± 0.43	17.1 ± 0.33	16.9 ± 0.04	16.8 ± 0.28

Hematology at Day 28							
	**1. Untreated**	**2. ICM. V5**	**3. ICM. CBh-hAP4B1**	**4. ICM. SYN-hAP4B1**	**5. ICM. AP4B1-AP4B1**	**6. ICM. MEP-hAP4B1**	**7. ICM. Cbh-mAP4B1**
PLT x10^3/uL	1041 ± 187	1135 ± 230	1122 ± 163	1068 ± 266	1049 ± 182	864 ± 51	877 ± 106
MPV fL	7 ± 0.09	7 ± 0.11	7 ± 0.05	6.9 ± 0.07	6.9 ± 0.08	7 ± 0.03	7.1 ± 0.04
%NEUT	7.9 ± 1.61	6.6 ± 2.06	6.6 ± 0.8	6.7 ± 2.02	7.1 ± 0.59	9.1 ± 0.93	9.2 ± 0.9
%LYMPH	86 ± 2.46	87 ± 2.31	86.2 ± 1.27	86.3 ± 2.92	85.5 ± 1.64	82.7 ± 1.03	82.8 ± 1.13
%MONO	3.6 ± 0.62	3.9 ± 0.62	4.4 ± 0.53	4.3 ± 0.55	4.5 ± 1.37	4.9 ± 0.2	4.7 ± 0.61
%EOS	1.5 ± 0.23	1.7 ± 0.36	2 ± 0.21	1.9 ± 0.3	2.1 ± 0.26	2.2 ± 0.35	2.2 ± 0.24
%BASO	1.2 ± 0.32	0.8 ± 0.24	0.9 ± 0.1	0.8 ± 0.38	0.9 ± 0.21	1.1 ± 0.13	1.1 ± 0.17
%RETIC	3.7 ± 0.05	3.6 ± 0.13	3.5 ± 0.16	3.4 ± 0.16	3.5 ± 0.08	3.4 ± 0.11	3.4 ± 0.09
#RETIC 10^9cells/L	391 ± 10	372 ± 16	361 ± 15	359 ± 19	359 ± 20	350 ± 9	350 ± 11
#NEUT 10^3cells/ul	0.4 ± 0.06	0.4 ± 0.07	0.4 ± 0.07	0.5 ± 0.07	0.5 ± 0.05	0.5 ± 0.05	0.5 ± 0.05
#LYMPH 10^3cells/ul	4.5 ± 0.41	5.1 ± 0.61	5.5 ± 0.38	5.8 ± 0.79	5.5 ± 0.43	5.1 ± 0.57	5.1 ± 0.6
#MONO 10^3cells/ul	0.18 ± 0.03	0.24 ± 0.07	0.3 ± 0.06	0.3 ± 0.05	0.3 ± 0.09	0.3 ± 0.04	0.3 ± 0.07
#EOS 10^3cells/ul	0.1 ± 0.01	0.1 ± 0.04	0.1 ± 0.02	0.1 ± 0.02	0.1 ± 0.02	0.1 ± 0.03	0.1 ± 0.03
#BASO 10^3cells/ul	0.1 ± 0.01	0 ± 0.01	0.1 ± 0.01	0.1 ± 0.01	0.1 ± 0.01	0.1 ± 0.01	0.1 ± 0.01
LCR %	5.2 ± 0.48	4.7 ± 0.54	4.6 ± 0.37	4.4 ± 0.58	4.4 ± 0.47	4.6 ± 0.16	4.8 ± 0.22
NRBC# 10^3cells/ul	0 ± 0.01	0 ± 0	0 ± 0.01	0 ± 0.01	0 ± 0.01	0 ± 0.01	0 ± 0.01
NRBC% %	0.2 ± 0.12	0.2 ± 0.07	0.3 ± 0.16	0.3 ± 0.13	0.3 ± 0.07	0.4 ± 0.11	0.3 ± 0.15

Hematology at Day 28							
	1. Untreated	2. ICM. V5	3. ICM. CBh-hAP4B1	4. ICM. SYN-hAP4B1	5. ICM. AP4B1-AP4B1	6. ICM. MEP-hAP4B1	7. ICM. Cbh-mAP4B1
RET-HE 0	18 ± 0.05	17.9 ± 0.11	17.9 ± 0.13	18 ± 0.14	18 ± 0.13	18 ± 0.1	18.1 ± 0.34
IRF %	57.7 ± 1.63	57.6 ± 1.6	57.7 ± 1.21	55.8 ± 1.45	56.2 ± 1.13	55.7 ± 0.95	55.5 ± 1.08
RDW-SD 0	23 ± 0.34	23.4 ± 0.37	23.4 ± 1.31	23.1 ± 0.43	23.3 ± 0.34	23.1 ± 0.21	23.2 ± 0.32
LFR %	42.3 ± 1.63	42.4 ± 1.6	42.3 ± 1.21	44.2 ± 1.45	43.8 ± 1.13	44.3 ± 0.95	44.5 ± 1.08
MFR %	19.6 ± 0.44	19.6 ± 0.56	19.4 ± 0.5	19.8 ± 0.32	20 ± 0.41	20.4 ± 0.51	19.8 ± 0.96
HFR %	38.2 ± 1.88	38 ± 1.8	38.3 ± 0.76	36.1 ± 1.26	36.2 ± 1.29	35.3 ± 1.14	35.7 ± 1.51
IPF %	0.2 ± 0.05	0.2 ± 0.03	0.2 ± 0.02	0.2 ± 0.26	0.2 ± 0.02	0.2 ± 0.02	0.2 ± 0.02
PLT-I 10^3/uL	1426 ± 296	1538 ± 333	1534 ± 412	1467 ± 370	1442 ± 274	1174 ± 75	1207 ± 166
PLT-O 10^3/uL	998 ± 182	1106 ± 213	1100 ± 150	1059 ± 250	1039 ± 171	867 ± 50	879 ± 103
PLT-F 10^3/uL	1041 ± 187	1135 ± 230	1122 ± 163	1068 ± 266	1049 ± 182	864 ± 51	877 ± 106
PCT %	1 ± 0.2	1.1 ± 0.23	1.1 ± 0.3	1 ± 0.25	1 ± 0.19	0.8 ± 0.05	0.8 ± 0.12
PDW fL	7.2 ± 0.14	7.2 ± 0.16	7.3 ± 0.1	7.3 ± 0.17	7.3 ± 0.13	7.4 ± 0.06	7.5 ± 0.11

Hematology at 1 year									
	**1. Untreated**	**2. ICM. AAV9/V5**	**3. ICM. AAV9/CBh-AP4B1**	**4. ICM. AAV9/SYN-AP4B1**	**5. ICM. AAV9/pAP4-AP4B1**	**6. ICM. AAV9/pMEP-AP4B1**	**7. ICM. AAV9/CBh-mAP4B1**	**8. IV. AAV9/Cbh-AP4B1**	**9. IV. AAV9/SYN-AP4B1**
WBC x10^3/uL	4.3 ± 0.37	6.4 ± 1.47	6.4 ± 0.55	7.6 ± 0.97	7.7 ± 1.45	9.6 ± 1.53	6.1 ± 1.3	5.1 ± 1.28	8.6 ± 1.51
RBC x10^6/uL	10.2 ± 0.17	10.2 ± 0.11	10 ± 0.08	9.9 ± 0.24	10.1 ± 0.25	10.0 ± 0.3	10.4 ± 0.28	9.7 ± 0.73	11.0 ± 0.17
HGB g/dL	14.3 ± 0.08	14.3 ± 0.14	14.2 ± 0.15	13.7 ± 0.34	14.1 ± 0.32	13.9 ± 0.24	14.7 ± 0.38	13.5 ± 0.91	14.8 ± 0.14
HCT %	48.2 ± 0.41	48.6 ± 0.54	48.2 ± 0.41	47.2 ± 1.11	48.3 ± 1.06	47.9 ± 0.76	49.1 ± 1.36	45.9 ± 2.64	50.5 ± 0.51
MCV fL	47.6 ± 0.53	47.7 ± 0.17	48.2 ± 0.38	47.4 ± 0.28	47.6 ± 0.46	48.1 ± 0.88	47.1 ± 0.26	47.7 ± 1.3	46.0 ± 0.29
MCH pg	14.1 ± 0.18	14.1 ± 0.08	14.2 ± 0.11	13.8 ± 0.09	13.9 ± 0.14	14.0 ± 0.24	14.1 ± 0.09	14.0 ± 0.22	13.5 ± 0.1
MCHC g/dL	29.7 ± 0.11	29.5 ± 0.07	29.5 ± 0.14	29.0 ± 0.05	29.1 ± 0.08	29.0 ± 0.11	29.9 ± 0.1	29.4 ± 0.45	29.4 ± 0.09
RDW %	17.4 ± 0.28	17.3 ± 0.15	17.3 ± 0.22	17.4 ± 0.18	17.5 ± 0.21	18.1 ± 0.55	17.9 ± 0.28	17.8 ± 0.36	18.4 ± 0.29
PLT x10^3/uL	1710 ± 158	1545 ± 73	1547 ± 101	1593 ± 93	1710 ± 96	1442 ± 210	1649 ± 96	1616 ± 310	1887 ± 126
MPV fL	7.1 ± 0.08	7.2 ± 0.05	7.2 ± 0.04	7.3 ± 0.09	7.2 ± 0.06	7.7 ± 0.49	7.2 ± 0.05	7.6 ± 0.51	6.9 ± 0.08
%NEUT	15.8 ± 1.6	14.7 ± 1.25	10.8 ± 0.8	9.9 ± 0.86	12 ± 1.19	12.1 ± 2.3	12.8 ± 1.52	17.8 ± 3.13	11.6 ± 1.48
%LYMPH	76.5 ± 1.83	79.9 ± 1.36	83.1 ± 1	84.5 ± 1.12	81.8 ± 1.95	81.5 ± 3.45	81.3 ± 1.89	72.4 ± 5.43	83.4 ± 1.81
%MONO	4.4 ± 0.56	3.5 ± 0.44	4.0 ± 0.41	3.7 ± 0.42	3.9 ± 0.64	4.6 ± 1.2	4.0 ± 0.39	6.0 ± 2.54	2.9 ± 0.26
%EOS	2.3 ± 0.42	1.3 ± 0.16	1.7 ± 0.15	1.5 ± 0.13	1.9 ± 0.25	1.7 ± 0.26	1.4 ± 0.23	3.2 ± 1.5	1.9 ± 0.23
%BASO	1.0 ± 0.37	0.5 ± 0.05	0.5 ± 0.08	0.4 ± 0.08	0.4 ± 0.08	0.2 ± 0.05	0.5 ± 0.07	0.6 ± 0.12	0.3 ± 0.09
%RETIC	3.2 ± 0.14	3.6 ± 0.1	3.9 ± 0.12	3.8 ± 0.13	4.2 ± 0.35	5.4 ± 1.51	3.7 ± 0.09	6.9 ± 3.19	3.6 ± 0.06
#RETIC 10^9cells/L	328 ± 14	372 ± 12	387 ± 12	374 ± 17	417 ± 25	510 ± 118	388 ± 15	535 ± 148	400 ± 10
#NEUT 10^3cells/ul	0.7 ± 0.1	0.9 ± 0.16	0.7 ± 0.07	0.7 ± 0.04	0.8 ± 0.08	1.2 ± 0.39	0.7 ± 0.07	0.8 ± 0.17	1.0 ± 0.19

Hematology at 1 year									
	1. Untreated	2. ICM. AAV9/V5	3. ICM. AAV9/CBh -AP4B1	4. ICM. AAV9/SYN -AP4B1	5. ICM. AAV9/pAP 4-AP4B1	6. ICM. AAV9/pM EP-AP4B1	7. ICM. AAV9/CBh -mAP4B1	8. IV. AAV9/Cbh -AP4B1	9. IV. AAV9/SYN -AP4B1
#LYMPH 10^3cells/ ul	3.3 ± 0.3	5.2 ± 1.24	5.3 ± 0.49	6.5 ± 0.92	6.5 ± 1.36	7.8 ± 1.22	5.1 ± 1.19	3.9 ± 1.16	7.3 ± 1.3
#MONO 10^3cells/ ul	0.18 ± 0.02	0.23 ± 0.05	0.25 ± 0.02	0.25 ± 0.02	0.26 ± 0.03	0.43 ± 0.17	0.24 ± 0.04	0.32 ± 0.14	0.23 ± 0.03
#EOS 10^3cells/ ul	0.1 ± 0.02	0.09 ± 0.02	0.11 ± 0.01	0.12 ± 0.02	0.14 ± 0.02	0.14 ± 0.02	0.09 ± 0.02	0.14 ± 0.05	0.15 ± 0.03
#BASO 10^3cells/ ul	0.04 ± 0.02	0.04 ± 0.01	0.03 ± 0	0.03 ± 0	0.02 ± 0	0.02 ± 0.01	0.03 ± 0	0.02 ± 0	0.022 ± 0
LCR %	5.6 ± 0.3	6.5 ± 0.27	5.9 ± 0.27	7.4 ± 0.86	6.1 ± 0.29	10.7 ± 4.24	6.4 ± 0.2	9.5 ± 3.84	5.5 ± 0.61
NRBC# 10^3cells/ ul	0.02 ± 0	0.01 ± 0	0.02 ± 0.01	0 ± 0	0.01 ± 0	0.02 ± 0.02	0 ± 0	0.03 ± 0.02	0 ± 0
NRBC% %	0.35 ± 0.06	0.15 ± 0.07	0.4 ± 0.14	0.08 ± 0.06	0.05 ± 0.03	0.17 ± 0.11	0.05 ± 0.04	0.63 ± 0.43	0 ± 0
RET-HE 0	17.7 ± 0.32	17.7 ± 0.13	17.7 ± 0.13	17.1 ± 0.1	17.4 ± 0.17	17.2 ± 0.14	17.7 ± 0.14	17.2 ± 0.25	17 ± 0.19
IRF %	52.3 ± 1.03	56.4 ± 1.13	60 ± 1.21	57.7 ± 0.65	60.6 ± 1.3	59.1 ± 2.43	54.5 ± 1.48	58.6 ± 3.36	56.3 ± 0.83
RDW-SD 0	23.9 ± 0.32	24.2 ± 0.29	24.4 ± 0.54	24.4 ± 0.55	23.8 ± 0.26	25.5 ± 1.62	24.0 ± 0.2	25.6 ± 1.99	23.5 ± 0.31
LFR %	47.8 ± 1.03	43.6 ± 1.13	40.0 ± 1.21	42.3 ± 0.65	39.4 ± 1.3	41.0 ± 2.43	45.5 ± 1.48	41.4 ± 3.36	43.7 ± 0.83
MFR %	18.0 ± 0.4	17.4 ± 0.49	16.9 ± 0.11	17.9 ± 0.87	16.3 ± 0.51	16.4 ± 0.67	16.9 ± 0.39	15.4 ± 0.47	18.3 ± 0.28
HFR %	34.3 ± 1.02	39.0 ± 1.6	43.1 ± 1.25	39.8 ± 1.23	44.3 ± 1.2	42.7 ± 2.9	37.6 ± 1.58	43.2 ± 3.73	38.1 ± 0.77
IPF %	0.20 ± 0.03	0.28 ± 0.04	0.20 ± 0	0.46 ± 0.15	0.27 ± 0.02	0.43 ± 0.17	0.20 ± 0	1.30 ± 0.97	0.32 ± 0.06
PLT-I 10^3/uL	2426 ± 191	2245 ± 115	2324 ± 163	2332 ± 152	2525 ± 128	2074 ± 320	2499 ± 187	2171 ± 468	2577 ± 167

Hematology at 1 year									
	1. Untreated	2. ICM. AAV9/V5	3. ICM. AAV9/CBh -AP4B1	4. ICM. AAV9/SYN -AP4B1	5. ICM. AAV9/pAP 4-AP4B1	6. ICM. AAV9/pM EP-AP4B1	7. ICM. AAV9/CBh -mAP4B1	8. IV. AAV9/Cbh -AP4B1	9. IV. AAV9/SYN -AP4B1
PLT-O 10^3/uL	1648 ± 167	1462 ± 81	1434 ± 100	1485 ± 76	1582 ± 92	1347 ± 201	1506 ± 92	1504 ± 315	1803 ± 139
PLT-F 10^3/uL	1593 ± 135	1545 ± 73	1547 ± 101	1593 ± 93	1710 ± 96	1442 ± 210	1649 ± 96	1616 ± 310	1887 ± 126
PCT %	1.7 ± 0.13	1.6 ± 0.08	1.7 ± 0.12	1.7 ± 0.11	1.8 ± 0.08	1.5 ± 0.21	1.8 ± 0.12	1.5 ± 0.33	1.8 ± 0.1
PDW fL	7.4 ± 0.13	7.5 ± 0.05	7.7 ± 0.06	7.6 ± 0.12	7.6 ± 0.11	7.4 ± 0.1	7.8 ± 0.07	7.4 ± 0.11	7.3 ± 0.13

Serum Chemistry at Day 28					
**Vector**	**Route**	**ALP IU/L**	**ALT IU/L**	**AST IU/L**	**LIPASE U/L**
1. Untreated	-	151 ± 9	33.3 ± 8.8	181 ± 129	260 ± 120
2. AAV9-V5 Control	ICM	154 ± 10	34.7 ± 5.5	69 ± 13	512 ± 349
3. AAV9-CBh-hAP4B1	ICM	145 ± 10	36.2 ± 6.1	76 ± 20	438 ± 359
4. AAV9-SYN-hAP4B1	ICM	153 ± 12	37.3 ± 6.5	95 ± 26	122 ± 27
5. AAV9-pAP4B1-hAP4B1	ICM	155 ± 11	24 ± 1.1	53 ± 9	123 ± 40
6. AAV9-MEP-hAP4B1	ICM	147 ± 12	31.5 ± 3.9	99 ± 19	759 ± 427
7. AAV9-Cbh-mAP4B1	ICM	154 ± 11	30.2 ± 4.4	73 ± 16	1441 ± 1276

Serum Chemistry at 1-year					
**Vector**	**Route**	**ALP IU/L**	**ALT IU/L**	**AST IU/L**	**LIPASE U/L**
1. Untreated	-	102 ± 8.1	39 ± 6.7	62 ± 6.5	86 ± 6.4
2. AAV9-V5 Control	ICM	129 ± 15.7	65 ± 23.5	77 ± 11.1	93 ± 5.8

3. AAV9-CBh-hAP4B1	ICM	134 ± 23.6	43 ± 4.7	54 ± 3.5	84 ± 6.7
4. AAV9-SYN-hAP4B1	ICM	111 ± 16.1	40 ± 3.4	58 ± 10.2	81 ± 4.8
5. AAV9-pAP4B1-hAP4B1	ICM	144 ± 37.7	34 ± 1.7	54 ± 3.9	80 ± 1.9
6. AAV9-MEP-hAP4B1	ICM	98 ± 8.2	40 ± 5.7	55 ± 7.6	72 ± 4.5
7. AAV9-Cbh-mAP4B1	ICM	127 ± 20.8	46 ± 8.9	60 ± 4.3	84 ± 3.4
8. AAV9-CBh-hAP4B1	IV	110 ± 15.7	66 ± 31.9	80 ± 22.1	73 ± 5.6
9. AAV9-SYN-hAP4B1	IV	173 ± 38.5	37 ± 3.6	57 ± 4.2	76 ± 3.3

Liver Histology Day 28							
	**1. Untreated**	**2. ICM, V5**	**3. ICM, CBh-hAP4B1**	**4. ICM, SYN-hAP4B1**	**5. ICM, AP4B1-AP4B1**	**6. ICM, MEP-hAP4B1**	**7. ICM Cbh-mAP4B1**
**Inflammation**							
None	2	1	0	3	3	0	1
Minimal	4	3	4	3	3	6	3
Mild	0	2	2	0	0	0	2
Moderate	0	0	0	0	0	0	0
Severe	0	0	0	0	0	0	0
**Average**	**0.67**	**1.17**	**1.33**	**0.50**	**0.50**	**1.00**	**1.17**
**SEM**	**0.23**	**0.34**	**0.23**	**0.27**	**0.24**	**0.00**	**0.34**
**Necrosis**							
None	3	1	1	3	3	1	2
Minimal	3	5	5	3	3	5	4

Liver Histology Day 28							
	**1. Untreated**	**2. ICM, V5**	**3. ICM, CBh-hAP4B1**	**4. ICM, SYN-hAP4B1**	**5. ICM, AP4B1-AP4B1**	**6. ICM, MEP-hAP4B1**	**7. ICM Cbh-mAP4B1**
Mild	0	0	0	0	0	0	0
Moderate	0	0	0	0	0	0	0
Severe	0	0	0	0	0	0	0
**Average**	**0.50**	**0.83**	**0.83**	**0.50**	**0.50**	**0.83**	**0.67**
**SEM**	**0.24**	**0.18**	**0.18**	**0.27**	**0.24**	**0.18**	**0.23**
**Fibrosis**							
None	2	4	0	4	4	3	3
Minimal	4	1	6	2	2	1	2
Mild	0	1	0	0	0	2	1
Moderate	0	0	0	0	0	0	0
Severe	0	0	0	0	0	0	0
**Average**	**0.67**	**0.50**	**1.00**	**0.33**	**0.33**	**0.83**	**0.67**
**SEM**	**0.23**	**0.37**	**0.00**	**0.26**	**0.23**	**0.44**	**0.37**
**Steatosis**							
**Average**	**0**	**0**	**0**	**0**	**0**	**0**	

Liver Histology at 1 year								
**1. Untreated**	**2. ICM, V5**	**3. ICM, CBh-hAP4B1**	**4. ICM, SYN-hAP4B1**	**5. ICM, AP4B1-AP4B1**	**6. ICM, MEP-hAP4B1**	**7. ICM Cbh-mAP4B1**	**8. IVCBh-hAP4B1**	**9. IV SYN-hAP4B1**
**Inflammation**								
None	1	0	2	0	0	0	0	0
Minimal	1	6	3	4	5	2	4	0
Mild	2	0	1	1	1	3	2	3
Moderate	2	0	0	0	0	1	0	3
Severe	0	0	0	0	0	0	0	1
**Average**	**1.83**	**1.00**	**0.83**	**1.20**	**1.17**	**1.83**	**1.33**	**2.83**
**SEM**	**0.52**	**0.00**	**0.34**	**0.22**	**0.18**	**0.34**	**0.23**	**0.31**
**Necrosis**								
None	0	0	0	0	0	0	0	0
Minimal	5	2	6	2	4	5	5	6
Mild	1	3	0	2	1	1	0	1
Moderate	0	1	0	1	1	0	1	0
Severe	0	0	0	0	0	0	0	0
**Average**	**1.17**	**1.83**	**1.00**	**1.80**	**1.50**	**1.17**	**1.33**	**1.00**
**SEM**	**0.18**	**0.34**	**0.00**	**0.42**	**0.37**	**0.18**	**0.37**	**0.00**

Pre-necrosis								
None	0	0	0	0	0	0	0	0
Minimal	0	0	4	0	1	2	0	1
Mild	3	4	1	1	3	1	3	4
Moderate	2	1	0	3	0	2	1	1
Severe	0	0	0	0	1	0	1	0
**Average**	**2.40**	**2.20**	**1.20**	**2.75**	**2.20**	**2.00**	**2.60**	**1.80**
**SEM**	**0.24**	**0.20**	**0.20**	**0.25**	**0.49**	**0.45**	**0.40**	**0.18**
**Steatosis and Fibrosis**								
**Average**	**0**	**0**	**0**	**0**	**0**	**0**	**0**	**0**

Appendix Table S2. Tabulated summary of results of non-human primate GLP toxicology study.

This was a GLP study of the safety of AAV9/CBh-hAP4B1 by intra-cisterna magna injection of AAV vector that was a process comparable to the intended clinical product. As well as the standard general observations, weight gain, hematology, chemistry, coagulation and histopathological examinations, there was assessment of nerve conductance velocity as an index of potential functional impairment resulting from the known/previously reported DRG inflammation caused by AAV vectors. In addition, biodistribution and immune responses were measured. The following tables summarize group averages and standard error.

List of results

Study cohorts: Agreed with FDA at Pre-IND stage 24
Schedule of events 24
Weight -individual animals 25
Serum Chemistry 25
Complete blood count 28
Coagulation 32
Nerve conductance Velocity and Amplitude 33
Humoral immune response 33
Biodistribution 34
CNS Histopathology 35

Study cohorts: Agreed with FDA at Pre-IND stage			
**Group**	**Treatment vehicle or test item (vg/animal)**	**Sacrifice at 1 month # and sex**	**Sacrifice at 4 months # and sex**
1	Vehicle	1M, 1F	1M, 1F
2	1.35×10^{13} vg	-	1M, 2F
3	7.2×10^{13} vg	2M, 1F	1M, 2F

Schedule of events											
	**Pre**	**0**	**3**	**7**	**22**	**50**	**78**	**106**	**Daily**	**Weekly**	**Post mortem**
Vector / vehicle administration		X									
Morbidity / mortality									X		
General exam - weight										X	
Immune response Cellular and Humoral)	X				X	X	X	X			
Nerve conductance	X				X	X	X	X			
Serum chemistry	X			X	X	X		X			
CBC / coagulation	X			X	X	X		X			
Urine analysis	X			X	X	X		X			
Histology											X
Biodistribution											X
Gene Expression											X

Weight -individual animals																			
**Group**	**Gender**	**ID**	**4**	**7**	**14**	**21**	**28**	**35**	**42**	**49**	**56**	**63**	**70**	**77**	**84**	**91**	**98**	**105**	**112**
Cohort 1 Vehicle	M	220013002	4.25	4.5	4.8	4.6	4.55												
	F	220013015	3.8	3.85	4.05	4.05	4	4.1	4.15	4.1	3.95	3.95	3.95	4	4	4.1	4.15	4.1	4.05
	F	220013003	2.95	3.4	3.5	3.4	3.45	3.3	3.35	3.2	3.3	3.2	3.2	3.3	3.4	3.35	3.35	3.35	3.3
	M	220013004	2.85	3.05	3.2	3.2	3.35												
Cohort 2 Low dose	F	220013005	4.35	4.25	4.15	4.3	4.3	4.4	4.4	4.5	4.6	4.6	4.65	4.6	4.65	4.75	4.65	4.55	4.55
	M	220013006	3.05	3.05	3.15	3.1	3.1	3.1	3	3.05	3.05	3.1	3.1	3.1	3.1	3.15	3.15	3.1	3.1
	F	220013007	3.2	3.3	3.5	3.45	3.4	3.45	3.45	3.5	3.5	3.55	3.6	3.55	3.55	3.55	3.6	3.55	3.6
Cohort 3 High dose	M	220013008	4.9	4.95	5	4.9	5.05												
	F	220013009	4.7	4.65	4.7	4.65	4.65												
	M	220013010	3.95	3.85	3.9	3.9	3.95	3.95	3.9	3.95	4	3.95	4.05	4.05	3.95	4.05	3.95	4.1	4
	F	220013011	3.4	3.45	3.35	3.3	3.2												
	M	220013012	3	3	3	3	3.1	3.3	3.15	3.05	3.1	3	3.25	3.1	3	3.1	3.1	3.05	3.2
	F	220013013	2.7	2.6	2.6	2.7	2.7	2.85	2.75	2.8	2.85	2.75	3	2.85	2.95	2.9	2.85	2.85	2.9

Serum Chemistry					
**Cohort**	**Pre**	**Day 7**	**Day 22**	**Day 50**	**Day 106**
**ALT**					
1. PBS	43.5 ± 4.1	40.8 ± 2.8	42.8 ± 5.9	36 ± 7	34 ± 11
2. AAV9/CBh-hAP4B1 low dose	51 ± 5.2	48.3 ± 2.6	37.7 ± 4.8	42.7 ± 1.7	40.7 ± 2.4
3. AAV9/CBh-hAP4B1 high dose	52.2 ± 3.2	97.2 ± 19.4	41.8 ± 4.9	64 ± 19.6	57.7 ± 16.2
**AST**					
1. PBS	44 ± 4.3	49.3 ± 3.6	44.8 ± 2.7	42 ± 3	47.5 ± 0.5
2. AAV9/CBh-hAP4B1 low dose	37 ± 3.5	36.3 ± 3.4	44.3 ± 4.6	36.3 ± 3.3	31 ± 2.1
3. AAV9/CBh-hAP4B1 high dose	42.7 ± 3.2	59.5 ± 6.5	51.7 ± 4.4	45.3 ± 5.9	47.7 ± 8.2
**ALP**					
1. PBS	996 ± 49	869 ± 72	930 ± 58	811 ± 28	958 ± 10

Serum Chemistry					
**Cohort**	**Pre**	**Day 7**	**Day 22**	**Day 50**	**Day 106**
2. AAV9/CBh-hAP4B1 low dose	724 ± 34	627 ± 42	707 ± 45	751 ± 44	694 ± 64
3. AAV9/CBh-hAP4B1 high dose	686 ± 111	606 ± 83	580 ± 84	605 ± 75	659 ± 114
**TBIL**					
1. PBS	5.93 ± 1.06	4.23 ± 1	6.61 ± 1.92	2.97 ± 1.3	6.19 ± 1.85
2. AAV9/CBh-hAP4B1 low dose	5.68 ± 0.75	3.17 ± 0.34	2.92 ± 0.08	3.5 ± 0.17	5.17 ± 0.97
3. AAV9/CBh-hAP4B1 high dose	3.86 ± 0.65	3.32 ± 0.47	2.96 ± 0.48	3.13 ± 0.55	3.96 ± 0.6
	DBIL				
1. PBS	1.87 ± 0.26	2.11 ± 0.29	1.87 ± 0.45	1.59 ± 0.57	1.86 ± 0.44
2. AAV9/CBh-hAP4B1 low dose	2.1 ± 0.19	1.22 ± 0.07	1.6 ± 0.13	1.93 ± 0.03	2.02 ± 0.34
3. AAV9/CBh-hAP4B1 high dose	1.49 ± 0.28	1.89 ± 0.16	1.71 ± 0.18	1.6 ± 0.14	1.38 ± 0.18
	PROT				
1. PBS	81.3 ± 1.7	79.8 ± 1.3	81.3 ± 1.8	72.5 ± 2.5	74 ± 1
2. AAV9/CBh-hAP4B1 low dose	81.7 ± 0.9	78.3 ± 0.3	78.7 ± 0.7	79.7 ± 1.7	71.3 ± 0.7
3. AAV9/CBh-hAP4B1 high dose	80.3 ± 1.4	77.5 ± 1.8	75.8 ± 2.1	72 ± 3.1	70 ± 1.2
	ALB				
1. PBS	44.5 ± 0.6	45 ± 0.7	43.8 ± 0.8	42 ± 0	43.5 ± 1.5
2. AAV9/CBh-hAP4B1 low dose	42.7 ± 1.2	45.7 ± 0.7	43.3 ± 0.9	42 ± 1.5	42.3 ± 0.7
3. AAV9/CBh-hAP4B1 high dose	42.5 ± 1.8	43.3 ± 1.8	41 ± 1.8	41.3 ± 2.6	39.7 ± 3.2
	CREA				
1. PBS	81.5 ± 8	71.8 ± 7.4	76.3 ± 5.2	74 ± 13	83.5 ± 9.5
2. AAV9/CBh-hAP4B1 low dose	62 ± 10.5	60 ± 7.5	59.7 ± 5.2	71.3 ± 7.9	66.7 ± 6.5
3. AAV9/CBh-hAP4B1 high dose	85.7 ± 7.7	72.5 ± 7.2	76 ± 8.2	266 ± 192.2	75.7 ± 7.8
	UREA				
1. PBS	5.88 ± 0.15	5 ± 0.15	6.28 ± 0.14	4.1 ± 0.3	6.75 ± 0.35
2. AAV9/CBh-hAP4B1 low dose	5.8 ± 0.15	4.83 ± 0.19	6.27 ± 0.26	4.57 ± 0.17	5 ± 0.25
3. AAV9/CBh-hAP4B1 high dose	6.02 ± 0.09	5.12 ± 0.4	5.98 ± 0.26	4.83 ± 0.57	4.73 ± 0.55
	GLU				
1. PBS	4.16 ± 0.34	4.96 ± 0.25	4.38 ± 0.26	5.39 ± 0.39	4 ± 0.16
2. AAV9/CBh-hAP4B1 low dose	3.86 ± 0.14	4.72 ± 0.18	4.98 ± 0.71	4.69 ± 0.74	4.28 ± 0.35
3. AAV9/CBh-hAP4B1 high dose	4.2 ± 0.37	4.46 ± 0.43	3.84 ± 0.13	4.53 ± 0.53	4.54 ± 0.58

Serum Chemistry					
**Cohort**	**Pre**	**Day 7**	**Day 22**	**Day 50**	**Day 106**
	CK				
1. PBS	191 ± 28	162 ± 24	174 ± 17	115 ± 5	151 ± 3
2. AAV9/CBh-hAP4B1 low dose	154 ± 22	151 ± 21	164 ± 21	186 ± 6	137 ± 17
3. AAV9/CBh-hAP4B1 high dose	229 ± 25	205 ± 31	132 ± 9	163 ± 18	152 ± 29
	CHOL				
1. PBS	3.18 ± 0.28	2.9 ± 0.21	3.13 ± 0.24	2.75 ± 0.09	3.01 ± 0.12
2. AAV9/CBh-hAP4B1 low dose	2.86 ± 0.19	2.92 ± 0.17	3.45 ± 0.22	2.93 ± 0.31	3 ± 0.18
3. AAV9/CBh-hAP4B1 high dose	2.66 ± 0.23	2.54 ± 0.16	2.97 ± 0.3	2.33 ± 0.18	2.56 ± 0.25
	TRIG				
1. PBS	0.4 ± 0.06	0.46 ± 0.04	0.54 ± 0.09	0.39 ± 0.06	0.69 ± 0.13
2. AAV9/CBh-hAP4B1 low dose	0.43 ± 0.01	0.42 ± 0.01	0.33 ± 0.02	0.43 ± 0.03	0.34 ± 0.03
3. AAV9/CBh-hAP4B1 high dose	0.43 ± 0.06	0.44 ± 0.05	0.41 ± 0.03	0.47 ± 0.11	0.48 ± 0.03
	GLOB				
1. PBS	37.3 ± 1.7	35 ± 1	37.5 ± 1.6	31 ± 2	30 ± 0
2. AAV9/CBh-hAP4B1 low dose	38.3 ± 0.9	32.7 ± 0.9	35.3 ± 0.3	37 ± 0.6	29.3 ± 1.2
3. AAV9/CBh-hAP4B1 high dose	38.3 ± 0.8	34.5 ± 1.3	34.8 ± 1	30.7 ± 2.2	31 ± 3.2
	A/G				
1. PBS	1.21 ± 0.05	1.29 ± 0.05	1.18 ± 0.06	1.36 ± 0.1	1.45 ± 0.05
2. AAV9/CBh-hAP4B1 low dose	1.12 ± 0.04	1.38 ± 0.07	1.23 ± 0.04	1.13 ± 0.03	1.47 ± 0.08
3. AAV9/CBh-hAP4B1 high dose	1.12 ± 0.07	1.27 ± 0.08	1.19 ± 0.06	1.36 ± 0.13	1.33 ± 0.27
	CA				
1. PBS	2.32 ± 0.01	2.22 ± 0.02	2.54 ± 0.03	2.46 ± 0.03	2.36 ± 0.09
2. AAV9/CBh-hAP4B1 low dose	2.33 ± 0.04	2.3 ± 0.04	2.65 ± 0.03	2.37 ± 0.03	2.37 ± 0.02
3. AAV9/CBh-hAP4B1 high dose	2.33 ± 0.04	2.2 ± 0.03	2.44 ± 0.04	2.38 ± 0.1	2.23 ± 0.07
	P				
1. PBS	1.67 ± 0.07	1.64 ± 0.14	1.67 ± 0.12	1.25 ± 0.1	1.52 ± 0.01
2. AAV9/CBh-hAP4B1 low dose	1.58 ± 0.11	1.52 ± 0.11	1.67 ± 0.11	1.4 ± 0.04	1.31 ± 0.07

Serum Chemistry					
**Cohort**	**Pre**	**Day 7**	**Day 22**	**Day 50**	**Day 106**
3. AAV9/CBh-hAP4B1 high dose	1.74 ± 0.07	1.47 ± 0.03	1.51 ± 0.08	1.13 ± 0.09	0.99 ± 0.04
	CL				
1. PBS	110.9 ± 0.8	108.7 ± 0.4	108.5 ± 0.6	109.9 ± 0.7	104.9 ± 1.8
2. AAV9/CBh-hAP4B1 low dose	110.1 ± 0.6	107.6 ± 0.6	110.5 ± 0.2	107.1 ± 0.5	106.4 ± 0.5
3. AAV9/CBh-hAP4B1 high dose	109.6 ± 0.5	108.4 ± 0.5	110.9 ± 0.6	115.2 ± 0.8	108.3 ± 1.3
	K				
1. PBS	4.13 ± 0.19	3.99 ± 0.19	3.83 ± 0.16	3.71 ± 0.38	3.76 ± 0.1
2. AAV9/CBh-hAP4B1 low dose	3.96 ± 0.21	3.74 ± 0.23	3.98 ± 0.07	3.69 ± 0.31	3.79 ± 0.22
3. AAV9/CBh-hAP4B1 high dose	4.14 ± 0.22	3.59 ± 0.12	3.62 ± 0.24	3.66 ± 0.32	3.44 ± 0.18
	NA				
1. PBS	148 ± 0.8	142 ± 0.5	144 ± 0.8	145 ± 0.5	149 ± 2.5
2. AAV9/CBh-hAP4B1 low dose	146 ± 0.3	145 ± 0	147 ± 0.6	144 ± 0.6	148 ± 1.2
3. AAV9/CBh-hAP4B1 high dose	148 ± 1.3	143 ± 0.7	145 ± 1.2	149 ± 2.2	145 ± 1

Complete blood count					
**Cohort**	**Pre**	**Day 7**	**Day 22**	**Day 50**	**Day 106**
**WBC**					
1. PBS	14 ± 2.5	11.6 ± 1.8	9 ± 0.7	9.2 ± 1.5	9.9 ± 1.2
2. AAV9/CBh-hAP4B1 low dose	9.5 ± 1.2	9.8 ± 0.9	8.9 ± 1.1	8.2 ± 0.7	6.9 ± 1
3. AAV9/CBh-hAP4B1 high dose	11.8 ± 2	8.8 ± 1	9.4 ± 1.3	8.7 ± 1.5	12.5 ± 0.7
	neut				
1. PBS	9.9 ± 2.2	5.7 ± 1.5	3.2 ± 0.3	3.4 ± 1.2	3.4 ± 1.1
2. AAV9/CBh-hAP4B1 low dose	5.3 ± 1	4 ± 0.3	3 ± 0.5	4.1 ± 0.3	2.5 ± 0.4
3. AAV9/CBh-hAP4B1 high dose	6.9 ± 1.8	2.7 ± 0.4	3.2 ± 0.4	3 ± 0.5	6.8 ± 2
	eos				
1. PBS	0.13 ± 0.05	0.31 ± 0.12	0.34 ± 0.11	0.48 ± 0.23	0.5 ± 0.14
2. AAV9/CBh-hAP4B1 low dose	0.12 ± 0.05	0.17 ± 0.03	0.19 ± 0.02	0.1 ± 0.03	0.15 ± 0.04
3. AAV9/CBh-hAP4B1 high dose	0.25 ± 0.09	0.27 ± 0.07	0.26 ± 0.09	0.24 ± 0.12	0.18 ± 0.09

Complete blood count					
**Cohort**	**Pre**	**Day 7**	**Day 22**	**Day 50**	**Day 106**
	baso				
1. PBS	0.033 ± 0.009	0.045 ± 0.01	0.033 ± 0.006	0.035 ± 0.005	0.045 ± 0.015
2. AAV9/CBh-hAP4B1 low dose	0.03 ± 0.006	0.057 ± 0.015	0.053 ± 0.012	0.03 ± 0.012	0.027 ± 0.009
3. AAV9/CBh-hAP4B1 high dose	0.047 ± 0.015	0.048 ± 0.013	0.057 ± 0.014	0.04 ± 0.01	0.043 ± 0.009
	lymp				
1. PBS	3.45 ± 0.81	4.8 ± 0.79	4.8 ± 0.5	4.65 ± 0.05	4.55 ± 0.65
2. AAV9/CBh-hAP4B1 low dose	3.53 ± 0.46	4.87 ± 0.58	5.03 ± 0.49	3.47 ± 0.84	3.7 ± 0.4
3. AAV9/CBh-hAP4B1 high dose	4.03 ± 1.01	4.97 ± 0.83	5.22 ± 0.88	4.9 ± 1.62	4.43 ± 1.04
	mono				
1. PBS	0.39 ± 0.07	0.53 ± 0.11	0.49 ± 0.1	0.53 ± 0.04	1.14 ± 0.47
2. AAV9/CBh-hAP4B1 low dose	0.38 ± 0.06	0.55 ± 0.13	0.51 ± 0.14	0.39 ± 0.15	0.48 ± 0.18
3. AAV9/CBh-hAP4B1 high dose	0.4 ± 0.05	0.63 ± 0.14	0.55 ± 0.09	0.43 ± 0.07	0.96 ± 0.33
	luc				
1. PBS	0.093 ± 0.013	0.135 ± 0.028	0.098 ± 0.014	0.14 ± 0.02	0.15 ± 0
2. AAV9/CBh-hAP4B1 low dose	0.087 ± 0.023	0.1 ± 0.015	0.09 ± 0.021	0.083 ± 0.023	0.067 ± 0.022
3. AAV9/CBh-hAP4B1 high dose	0.134 ± 0.036	0.157 ± 0.044	0.157 ± 0.039	0.127 ± 0.052	0.08 ± 0.023
	rbc				
1. PBS	6.68 ± 0.33	6.15 ± 0.39	6.58 ± 0.44	6.8 ± 0.8	6.95 ± 0.85
2. AAV9/CBh-hAP4B1 low dose	6.3 ± 0.32	6.2 ± 0.31	6.67 ± 0.33	6.57 ± 0.37	6.2 ± 0.49
3. AAV9/CBh-hAP4B1 high dose	6.7 ± 0.14	6.2 ± 0.22	6.62 ± 0.19	6.53 ± 0.32	6.83 ± 0.22
	hgb				48 ± 2
1. PBS	13.9 ± 0.29	12.7 ± 0.39	14 ± 0.56	14.2 ± 1.05	41.7 ± 2.4
2. AAV9/CBh-hAP4B1 low dose	13 ± 0.55	12.9 ± 0.6	14 ± 0.5	13.8 ± 0.76	45.3 ± 0.9
3. AAV9/CBh-hAP4B1 high dose	13.8 ± 0.27	12.7 ± 0.32	13.6 ± 0.26	13.2 ± 0.55	48 ± 2
**Hematocrit (%)**					
1. PBS	46 ± 1.6	42.8 ± 1.1	46.3 ± 1.7	48.5 ± 1.5	48 ± 2
2. AAV9/CBh-hAP4B1 low dose	42 ± 1.7	41.7 ± 1.8	45.7 ± 1.8	44 ± 2.1	41.7 ± 2.4
3. AAV9/CBh-hAP4B1 high dose	46 ± 1	41.7 ± 1.4	45 ± 1.2	44.7 ± 2	45.3 ± 0.9
	rdw				

Complete blood count					
**Cohort**	**Pre**	**Day 7**	**Day 22**	**Day 50**	**Day 106**
1. PBS	13.2 ± 0.39	13.4 ± 0.32	14.3 ± 0.56	13.1 ± 0.9	13.5 ± 0.9
2. AAV9/CBh-hAP4B1 low dose	13.2 ± 0.47	13.5 ± 0.23	13.8 ± 0.44	12.9 ± 0.52	13.2 ± 0.25
3. AAV9/CBh-hAP4B1 high dose	13.1 ± 0.25	13.5 ± 0.23	13.5 ± 0.22	13.2 ± 0.45	13.3 ± 0.55
	mcv				
1. PBS	69.5 ± 3.48	70 ± 3.24	70.8 ± 3.12	73 ± 7	70 ± 6
2. AAV9/CBh-hAP4B1 low dose	66 ± 0.58	67 ± 0.58	68 ± 0.58	67.3 ± 0.67	67.3 ± 1.2
3. AAV9/CBh-hAP4B1 high dose	68.2 ± 1.05	67.3 ± 0.88	68 ± 0.73	68 ± 1.53	66.7 ± 1.45
	mch				
1. PBS	20.9 ± 0.67	20.8 ± 0.75	21.3 ± 0.65	21 ± 1.05	21.3 ± 1.1
2. AAV9/CBh-hAP4B1 low dose	20.7 ± 0.12	20.8 ± 0.09	20.9 ± 0.27	21.2 ± 0.27	21.6 ± 0.28
3. AAV9/CBh-hAP4B1 high dose	20.6 ± 0.41	20.5 ± 0.44	20.6 ± 0.48	20.2 ± 0.87	20.4 ± 0.67
	mchc				
1. PBS	30.3 ± 0.85	30 ± 0.82	30 ± 0.71	29 ± 1	31 ± 1
2. AAV9/CBh-hAP4B1 low dose	31 ± 0	31.3 ± 0.33	30.7 ± 0.33	31.7 ± 0.33	32 ± 0.58
3. AAV9/CBh-hAP4B1 high dose	30.3 ± 0.49	30.2 ± 0.4	30.3 ± 0.33	30 ± 0.58	30.7 ± 0.33
	thr				
1. PBS	272 ± 14	297 ± 15	296 ± 16	286 ± 7	268 ± 35
2. AAV9/CBh-hAP4B1 low dose	309 ± 51	347 ± 40	312 ± 54	301 ± 58	283 ± 69
3. AAV9/CBh-hAP4B1 high dose	312 ± 12	348 ± 32	322 ± 32	312 ± 43	298 ± 34
	mvp				
1. PBS	9.9 ± 0.36	9.9 ± 0.45	10.2 ± 0.46	9.7 ± 0.8	10.5 ± 1.2
2. AAV9/CBh-hAP4B1 low dose	8.9 ± 0.65	9.2 ± 0.87	9.1 ± 1.09	9.5 ± 0.78	9.3 ± 0.7
3. AAV9/CBh-hAP4B1 high dose	9.4 ± 0.35	9.4 ± 0.36	9.8 ± 0.35	9.8 ± 0.83	9.1 ± 0.17
	ret				
1. PBS	31.5 ± 3	71.8 ± 8.1	65 ± 6	26.5 ± 4.5	39 ± 10
2. AAV9/CBh-hAP4B1 low dose	35 ± 11.5	70.3 ± 21.1	45.3 ± 4.3	38.3 ± 8	32.3 ± 6.7

Complete blood count					
Cohort	Pre	Day 7	Day 22	Day 50	Day 106
3. AAV9/CBh-hAP4B1 high dose	30 ± 3.7	54 ± 9.2	30.5 ± 7.8	40 ± 9.7	42 ± 9.3
**Neutrophils (%)**					
1. PBS	70 ± 5.2	48.5 ± 6.9	36.3 ± 3.2	35.5 ± 6.5	34.5 ± 7.5
2. AAV9/CBh-hAP4B1 low dose	55 ± 5.8	41.3 ± 3.4	33.7 ± 0.9	52 ± 9.2	36.3 ± 0.9
3. AAV9/CBh-hAP4B1 high dose	57 ± 6.5	31.8 ± 4.9	34.5 ± 3.6	37.7 ± 9.4	53 ± 12.9
**Eosinophils (%)**					
1. PBS	0.5 ± 0.3	2.3 ± 0.8	4.3 ± 1.3	5 ± 2	5 ± 2
2. AAV9/CBh-hAP4B1 low dose	1.3 ± 0.7	1.7 ± 0.3	2.3 ± 0.3	1.3 ± 0.3	2.3 ± 0.7
3. AAV9/CBh-hAP4B1 high dose	2.3 ± 0.6	2.8 ± 0.8	2.5 ± 0.8	2.3 ± 1.2	1.7 ± 0.9
**Basophils (%)**					
1. PBS	0 ± 0	0.3 ± 0.3	0.3 ± 0.3	0 ± 0	0.5 ± 0.5
2. AAV9/CBh-hAP4B1 low dose	0 ± 0	0.7 ± 0.3	0.7 ± 0.3	0.3 ± 0.3	0.3 ± 0.3
3. AAV9/CBh-hAP4B1 high dose	0.2 ± 0.2	0.7 ± 0.2	0.8 ± 0.2	0.3 ± 0.3	0.3 ± 0.3
**Lymphocyes (%)**					
1. PBS	25.5 ± 4.7	43 ± 6.6	52.8 ± 1.4	52 ± 8	46.5 ± 0.5
2. AAV9/CBh-hAP4B1 low dose	38.7 ± 5.8	49.7 ± 1.9	57 ± 1.5	41.3 ± 7.3	53.7 ± 2
3. AAV9/CBh-hAP4B1 high dose	35.8 ± 5.7	56.3 ± 4.6	54.8 ± 3.4	53.7 ± 8.2	36.3 ± 9.2
**Monocytes (%)**					
1. PBS	2.8 ± 0.3	4.5 ± 0.5	5.5 ± 1.2	3.5 ± 2.5	12.5 ± 6.5
2. AAV9/CBh-hAP4B1 low dose	4 ± 0	5.7 ± 0.9	5.7 ± 0.9	4.7 ± 1.5	6.7 ± 1.8
3. AAV9/CBh-hAP4B1 high dose	3.5 ± 0.4	6.7 ± 1.2	5.8 ± 0.6	5 ± 0	8 ± 3.1
**Leucocytes (%)**					
1. PBS	1 ± 0	1 ± 0	1.3 ± 0.3	1 ± 1	1.5 ± 0.5
2. AAV9/CBh-hAP4B1 low dose	1 ± 0	1 ± 0	1 ± 0	1 ± 0	1.3 ± 0.3
3. AAV9/CBh-hAP4B1 high dose	1.2 ± 0.3	1.5 ± 0.3	1.5 ± 0.2	1.3 ± 0.3	0.7 ± 0.3

Complete blood count					
Cohort	Pre	Day 7	Day 22	Day 50	Day 106
**Reticulocytes (%)**					
1. PBS	0.5 ± 0.3	1.3 ± 0.3	1 ± 0	0 ± 0	0.5 ± 0.5
2. AAV9/CBh-hAP4B1 low dose	0.3 ± 0.3	1.3 ± 0.3	1 ± 0	0.3 ± 0.3	0.3 ± 0.3
3. AAV9/CBh-hAP4B1 high dose	0.5 ± 0.2	0.8 ± 0.2	0.3 ± 0.2	0.7 ± 0.3	0.7 ± 0.3

Coagulation					
Cohort	Pre	Day 7	Day 22	Day 50	Day 106
**aPTT (sec)**					
1. PBS	24.9 ± 1.1	24.3 ± 0.4	24.5 ± 0.6	23.2 ± 0.2	24.9 ± 0.8
2. AAV9/CBh-hAP4B1 low dose	24.5 ± 0.2	24.9 ± 0	24.9 ± 0.5	25.2 ± 1	26.1 ± 0.3
3. AAV9/CBh-hAP4B1 high dose	26.7 ± 1.1	27.4 ± 0.8	27.1 ± 1.1	26 ± 0.4	26.9 ± 0.9
**PT (sec)**					
1. PBS	12.6 ± 0.3	12.7 ± 0.5	13.4 ± 0.4	13.2 ± 0.6	13.7 ± 0.3
2. AAV9/CBh-hAP4B1 low dose	12.6 ± 0.4	12.3 ± 0.4	12.7 ± 0.3	13 ± 0.3	13.2 ± 0.5
3. AAV9/CBh-hAP4B1 high dose	12.4 ± 0.2	12.6 ± 0.3	12.6 ± 0.5	12.8 ± 0.6	12.8 ± 0.3
**Fibrinogen (g/L)**					
1. PBS	2.2 ± 0.21	1.92 ± 0.08	1.72 ± 0.16	1.57 ± 0.18	1.67 ± 0.11
2. AAV9/CBh-hAP4B1 low dose	2.26 ± 0.26	2.08 ± 0.16	1.94 ± 0.12	2.19 ± 0.19	2.07 ± 0.18
3. AAV9/CBh-hAP4B1 high dose	2.31 ± 0.14	2.38 ± 0.16	2.18 ± 0.13	1.73 ± 0.19	1.97 ± 0.02

Nerve conductance Velocity and Amplitude					
**Cohort**	**Pre**	**Day 22**	**Day 50**	**Day 78**	**Day 106**
**Nerve conductance velocity - Sciatic**					
1. PBS	36.3 ± 1	36.5 ± 1.6	35.6	35.2	37.9
2. AAV9/CBh-hAP4B1 low dose	35.3 ± 1.1	31.8 ± 2.1	36.9 ± 2.4	34.1 ± 1.3	35.2 ± 1
3. AAV9/CBh-hAP4B1 high dose	38.7 ± 1.3	35.9 ± 1.2	36.4 ± 0.5	37.8 ± 1.3	38.5 ± 1.2
**Nerve conductance amplitude - Sciatic**					
1. PBS	44.2 ± 2.5	44.3 ± 0.8	48.6	48.2	48.6
2. AAV9/CBh-hAP4B1 low dose	45.2 ± 6.6	48.6 ± 7.3	49.1 ± 0.5	48.6 ± 0.5	44.4 ± 4.4
3. AAV9/CBh-hAP4B1 high dose	38 ± 4.6	46.6 ± 1.5	51.3 ± 2.6	48.4 ± 0.1	40.8 ± 8.1
**Nerve conductance velocity - Median**					
1. PBS	15.3 ± 0.8	14.9 ± 0.6	17.1	16.4	17.5
2. AAV9/CBh-hAP4B1 low dose	16.3 ± 0.4	14.5 ± 0.3	16.5 ± 0.4	17.5 ± 0.3	17.3 ± 0.2
3. AAV9/CBh-hAP4B1 high dose	17.8 ± 0.8	14.8 ± 0.2	17.6 ± 0.5	17 ± 0.4	16.6 ± 1.1
**Nerve conductance amplitude - Median**					
1. PBS	23.6 ± 1.5	23.4 ± 2.9	27.5	24.8	27.1
2. AAV9/CBh-hAP4B1 low dose	18.3 ± 1	19.8 ± 0.7	24.2 ± 0.1	24.3 ± 0.1	24.6 ± 0.2
3. AAV9/CBh-hAP4B1 high dose	22.5 ± 2.1	24.6 ± 2.3	24.4 ± 0.1	32.5 ± 7.8	40.5 ± 8.2
[1] Entries with no standard error represent cohorts with only 2 animals					

Humoral immune response						
			**Antibody titer**			
**Group**	**Gender**	**ID**	**Pre**	**Day 7**	**Day 29**	**Day113**
Cohort 1 Vehicle	M	220013002	766	650	500	n.d.
	F	220013015	-ve	-ve	-ve	n.d
	F	220013003	141	86	98	86
	M	220013004	193	195	194	194
Cohort 2 Low dose	F	220013005	180	>6250	5131	>6250

	M	220013006	154	5603	5465	>6250
	F	220013007	45	5464	1151	2513
Cohort 3 High dose	M	220013008	169	>6250	>6250	n.d.
	F	220013009	-ve	3540	5858	n.d
	M	220013010	91	>6250	>6250	n.d.
	F	220013011	-v1	4701	5429	>6250
	M	220013012	10	>6250	>6250	>6250
	F	220013013	-ve	>6250	>6250	>6250

Biodistribution			
**Organ**	**high dose 4 months**	**low dose**	**high dose, 1 month**
Cortex	6.8 ± 2.0 x 10^{5}	6.6 ± 3.5 x 10^{4}	5.4 ± 4.0 x 10^{5}
Cerebellum	8.8 ± 4.9 x 10^{5}	3.2 ± 2.1 x 10^{4}	4.1 ± 2.0 x 10^{5}
Thalamus	1.7 ± 0.9 x 10^{6}	1.1 ± 0.3 x 10^{5}	1.9 ± 0. 3 x 10^{5}
Cervical spinal cord	1.1 ± 0.6 x 10^{6}	3.9 ± 1.8 x 10^{5}	7.1 ± 2.5 x 10^{5}
Thoracic spinal cord	1.5 ± 0.5 x 10^{6}	3.2 ± 1.7 x 10^{5}	1.1 ± 0.3 x 10^{6}
Lumbar spinal cord	1.6 ± 0.5 x 10^{6}	2.5 ± 1.7 x 105	1.6 ± 0.05 x 10^{6}
Cervical DRG	1.2 ± 0.2 x 10^{6}	1.9 ± 1.3 x 10^{5}	2.2 ± 0.3 x 10^{6}
Thoracic DRG	2.0 ± 5.2 x 10^{5}	1.3 ± 1.0 x 10^{5}	1.2 ± 0. 5 x 10^{6}
Lumbar DRG	8.6 ± 3.5 x 10^{5}	1.5 ± 1.1 x 10^{5}	8.5 ± 6.9 x 10^{5}
Sciatic Nerve	5.1 ± 2.0 x 10^{3}	2.84 x 10^{2}	2.5 ± 1.4 x 10^{3}
Liver	8.5 ± 1.7 x 10^{6}	8.1 x 10^{1}	2.9 ± 2.5 x 10^{6}
Spleen	2.3 ±0.7 x 10^{5}	5.6 ± 3.8 x 10^{4}	3.4 ± 4.3 X 10^{6}
Kidney	3.6 ± 0.5 x 10^{4}	3.0 x 10^{2}	1.4 ± 0.6 x 10^{4}
Lung	9.0 ± 1.9 x 104	nd	2.2 ± 1.8 x 10^{4}
Ileum	1.1 ± 0.6 x 10^{4}	3.9 x 10^{3}	2.6 ± 2.4 x 10^{3}
Skeletal muscle	3.8 ± 0. 3 x 10^{4}	nd	4.4 ± 4.2 x 10^{3}
Heart	6.4 ± 1.5 x 10^{3}	nd	2.3 ± 2.3 x 10^{4}
Testes	2.0 x 10^{3}	nd	4.4 x 10^{2}
Ovary	5.0 x 10^{3}	nd	1.8 x 10^{4}

CNS histopathology<br>*Histopathology scores – none, minimal, mild, moderate, severe.*							
**Organ**	**Pathology**	**Incidence in cohort 1 (Vehicle, n=2 at 4wk; n=2 at 16wk))**		**Incidence in cohort 2: Low does AAV9-CBh-hAP4B1. (n=3 at 16wk)**		**Incidence in cohort 3: High dose AAV9-CBh-hAP4B1 (n=3 at 4; n=3 at 16wk)_**	
		**Minimal**	**Mild**	**Minimal**	**Mild**	**Minimal**	**Mild**
DRG	Neuron degeneration/necrosis	-	-	-	-	1	1
	Satellite glial cell, increased cellularity	-	-	-	-	4	1
	Mononuclear infiltrate,	-	-	-	-	2	1
	Neuron vacuolation	-	2	2	-	2	1
Spinal cord	Astrocytosis	-	-	3	-	2	2
	Nerve fiber degeneration	-	-	1	2	1	6
	Mononuclear cell infiltrate	-	-	-	-	1	-
	Neuronophagia	-	-	-	-	1	-
Brain	Astrocytosis	-	-	-	-	1	
	Decreased cellularity, neuron	-	-		-		1
	Mononuclear cell infiltrate1	-	-	1	-	3	
Sciatic Nerve	Neuron degeneration	-	-	1	-	1	-
1. Locations included perivascular, meninges, choroid plexus							

Appendix Table S3: **Estimate dose for human trials based on cerebrospinal fluid (CSF) volume.**

Organism	Route	Total dose (genome copies)	CSF volume (ml)[1]	Vector genomes (vg)/ml
Neonatal mice[2]<br>Study # ZLY-02	ICM	1×10^{11}	0.002	5×10^{13}
Adult mouse dosing study<br>Study # JPW-01	ICM	0.4×10^{11} to 2×10^{11}	0.04	1 to 5×10^{12}
Mouse long term toxicology<br>Study # JAX-131579	ICM	7.5×10^{10}	0.04	1.9×10^{12}
GLP NHP toxicology[3]<br>Study # ERBC - 20220013TCYP	ICM	High dose: 7.2×10^{13}	12	6×10^{12}
		Low dose: 1.4×10^{13}	12	1.2×10^{12}
Planned human trial dose[4]	ICM	4.0×10^{14}	75	5.3×10^{12}

1. Volumes from Chen et al. 2023. PMID: 36606687
2. Neonatal mice – assume CSF volume scales with body weight. Note injection volume was 5ul to vector may have spread beyond the intended injection site
3. Primates were an average of 4.3kg - smaller than typical so we propose to reduce Chen's 15ml estimate
4. Assuming 4–5-year-old patients. Please note that inclusion criteria will include 1–5-year-old patients

Appendix table S4: Exact P values for statistics

Figure 1 E	Symbol	P Value										
WT vs. Untreated	****	<0.0001										
WT vs. V5_empty	****	<0.0001										
WT vs. 2E5 vg/cell	****	<0.0001										
WT vs. 4E5 vg/cell	**	0.0026										
Untreated vs. V5_empty	ns	>0.9999										
Untreated vs. 2E5 vg/cell	ns	0.7241										
Untreated vs. 4E5 vg/cell	*	0.0161										
V5_empty vs. 2E5 vg/cell	ns	0.6545										
V5_empty vs. 4E5 vg/cell	*	0.0133										
2E5 vg/cell vs. 4E5 vg/cell	ns	0.1112										
	CEREBELLUM		SPINAL CORD		CEREBRUM		LIVER		HEART			
Figure 3 B-F	Symbol	P Value	Symbol	P Value	Symbol	P Value	Symbol	P Value	Symbol	P Value		

Wildtype vs. KO Untreated	ns	>0.9999	ns	>0.9999	ns	>0.9999	ns	>0.9999	ns	>0.9999		
Wildtype vs. KO V5-empty ICM	ns	>0.9999	ns	>0.9999	ns	>0.9999	ns	>0.9999	ns	0.8364		
Wildtype vs. KO Cbh-hAP4B1 ICM	***	0.0001	*	0.0167	*	0.0297	ns	0.2450	****	<0.0001		
Wildtype vs. KO V5-empty IV	ns	>0.9999	ns	>0.9999	ns	>0.9999	ns	>0.9999	ns	0.9770		
Wildtype vs. KO Cbh-hAP4B1 IV	ns	>0.9999	ns	>0.9999	ns	>0.9999	ns	0.7481	****	<0.0001		
KO Untreated vs. KO V5-empty ICM	ns	>0.9999	ns	>0.9999	ns	>0.9999	ns	>0.9999	ns	0.8718		
KO Untreated vs. KO Cbh-hAP4B1 ICM	***	0.0001	*	0.0167	*	0.0298	ns	0.2479	****	<0.0001		
KO Untreated vs. KO V5-empty IV	ns	>0.9999	ns	>0.9999	ns	>0.9999	ns	>0.9999	ns	0.9865		
KO Untreated vs. KO Cbh-hAP4B1 IV	ns	>0.9999	ns	>0.9999	ns	>0.9999	ns	0.7524	****	<0.0001		
KO V5-empty ICM vs. KO Cbh-hAP4B1 ICM	***	0.0001	*	0.0171	*	0.0298	ns	0.2463	****	<0.0001		
KO V5-empty ICM vs. KO V5-empty IV	ns	>0.9999	ns	>0.9999	ns	>0.9999	ns	>0.9999	ns	0.9963		

KO V5-empty ICM vs. KO Cbh-hAP4B1 IV	ns	>0.9999	ns	>0.9999	ns	>0.9999	ns	0.7500	****	<0.0001		
KO Cbh-hAP4B1 ICM vs. KO V5-empty IV	***	0.0001	*	0.0167	*	0.0297	ns	0.2434	****	<0.0001		
**KO Cbh-hAP4B1 ICM vs. KO Cbh-hAP4B1 IV**	***	**0.0001**	*	**0.0206**	*	**0.0312**	**ns**	**0.9107**	**ns**	**0.9953**		
KO V5-empty IV vs. KO Cbh-hAP4B1 IV	ns	>0.9999	ns	>0.9999	ns	>0.9999	ns	0.7455	****	<0.0001		
	CEREBRUM		SPINAL CORD		CEREBELLUM							
FIGURE 3 H,J,L	Symbol	P Value	Symbol	P Value	Symbol	P Value						
Hom V5-AP4B1 ICM vs. Hom V5 only ICM	*	0.0111	**	0.0049	*	0.0207						
	CLASPING SCORE 3MPT		CLASPING SCORE 6MPT		CLASPING SCORE 9MPT		ROTAROD 6MPT					
FIGURE 4	Symbol	P Value	Symbol	P Value	Symbol	P Value	Symbol	P Value				
WT vs. KO untreated	ns	0.2372	****	<0.0001	****	<0.0001	*	0.0150				
WT vs. KO V5-empty	ns	0.0780	***	0.0001	****	<0.0001	*	0.0115				

WT vs. KO Cbh-hAP4B1	ns	0.8299	ns	0.3203	ns	0.2636	ns	0.9658				
WT vs. KO SYN-hAP4B1	ns	0.6764	ns	0.4941	ns	0.1793	ns	0.5899				
KO untreated vs. KO V5-empty	ns	0.9499	ns	0.9999	ns	0.9898	ns	0.9959				
KO untreated vs. KO Cbh-hAP4B1	ns	0.8299	*	0.0201	*	0.0291	ns	0.0756				
KO untreated vs. KO SYN-hAP4B1	ns	0.9739	*	0.0272	ns	0.1263	ns	0.5188				
KO V5-empty vs. KO Cbh-hAP4B1	ns	0.4514	*	0.0272	*	0.0165	ns	0.0533				
KO V5-empty vs. KO SYN-hAP4B1	ns	0.7325	*	0.0335	ns	0.0711	ns	0.3807				
KO Cbh-hAP4B1 vs. KO SYN-hAP4B1	ns	0.9962	ns	0.9999	ns	0.9954	ns	0.9067				
	CORPUS CALLOSUM THICKNESS (5A)		LATERAL VENTRICLE SIZE (5B)		PLASMA NFL (5D)		CSF NFL (5D)					
FIGURE 5	Symbol	P Value	Symbol	P Value	Symbol	P Value	Symbol	P Value				

WT vs. KO untreated	/	/	/	/	****	<0.0001	ns	0.3788				
Wildtype vs. KO V5-empty	****	<0.0001	****	<0.0001	****	<0.0001	ns	0.4873				
Wildtype vs. KO CBh-hAP4B1	ns	0.2149	ns	0.2719	ns	>0.9999	ns	0.9993				
Wildtype vs. KO SYN-hAP4B1	ns	0.1947	ns	0.8654	ns	>0.9999	ns	0.9800				
KO untreated vs. KO V5-empty	/	/	/	/	ns	0.2602	ns	0.9996				
KO untreated vs. KO CBh-hAP4B1	/	/	/	/	****	<0.0001	ns	0.2834				
KO untreated vs. KO SYN-hAP4B1	/	/	/	/	****	<0.0001	ns	0.6615				
KO V5-empty vs. KO CBh-hAP4B1	***	0.0001	**	0.0013	****	<0.0001	ns	0.3756				
KO V5-empty vs. KO SYN-hAP4B1	***	0.0001	****	<0.0001	****	<0.0001	ns	0.7767				
KO CBh-hAP4B1 vs. KO SYN-hAP4B1	ns	>0.9999	ns	0.7253	ns	>0.9999	ns	0.9316				
	CEREBELLUM		SPINAL CORD		CEREBRUM		BRAINSTEM		LIVER		HEART	

FIGURE 5E	Symbol	P Value	Symbol	P Value	Symbol	P Value	Symbol	P Value	Symbol	P Value	Symbol	P Value
Wildtype vs. KO Untreated	ns	>0.9999	ns	>0.9999	ns	>0.9999	ns	>0.9999	ns	>0.9999	ns	>0.9999
Wildtype vs. KO V5-empty	ns	>0.9999	ns	>0.9999	ns	>0.9999	ns	>0.9999	ns	>0.9999	ns	>0.9999
Wildtype vs. KO CBh-hAP4B1	ns	0.3866	ns	0.9976	**	0.0013	ns	0.9389	ns	0.9314	ns	0.7522
Wildtype vs. KO SYN-hAP4B1	ns	0.4583	ns	0.9996	ns	0.8263	****	<0.0001	ns	0.7751	ns	0.9476
KO Untreated vs. KO V5-empty	ns	>0.9999	ns	>0.9999	ns	>0.9999	ns	>0.9999	ns	>0.9999	ns	>0.9999
KO Untreated vs. KO CBh-hAP4B1	ns	0.3855	ns	0.9976	**	0.0013	ns	0.9357	ns	0.9314	ns	0.7484
KO Untreated vs. KO SYN-hAP4B1	ns	0.4571	ns	0.9996	ns	0.8252	****	<0.0001	ns	0.7752	ns	0.9459
KO V5-empty vs. KO CBh-hAP4B1	ns	0.3901	ns	0.9979	**	0.0013	ns	0.9390	ns	0.9316	ns	0.7528
KO V5-empty vs. KO SYN-hAP4B1	ns	0.4620	ns	0.9997	ns	0.8257	****	<0.0001	ns	0.7754	ns	0.9478
KO CBh-hAP4B1 vs. KO SYN-hAP4B1	ns	>0.9999	ns	>0.9999	*	0.0360	**	0.0016	ns	0.9962	ns	0.9904

	CEREBELLUM		SPINAL CORD		CEREBRUM		BRAINSTEM		LIVER		HEART	
FIGURE 5F	Symbol	P Value	Symbol	P Value	Symbol	P Value	Symbol	P Value	Symbol	P Value	Symbol	P Value
Wildtype vs. KO Untreated	ns	>0.9999	ns	>0.9999	ns	>0.9999	ns	>0.9999	ns	>0.9999	ns	>0.9999
Wildtype vs. KO V5-empty	ns	>0.9999	ns	>0.9999	ns	>0.9999	ns	>0.9999	ns	>0.9999	ns	>0.9999
Wildtype vs. KO CBh-hAP4B1	ns	0.7397	ns	0.9943	*	0.0167	ns	0.6316	ns	>0.9999	ns	0.9996
Wildtype vs. KO SYN-hAP4B1	ns	0.1703	ns	>0.9999	ns	>0.9999	ns	0.0991	ns	>0.9999	ns	>0.9999
KO Untreated vs. KO V5-empty	ns	>0.9999	ns	>0.9999	ns	>0.9999	ns	>0.9999	ns	>0.9999	ns	>0.9999
KO Untreated vs. KO CBh-hAP4B1	ns	0.7397	ns	0.9943	*	0.0167	ns	0.6316	ns	>0.9999	ns	0.9996
KO Untreated vs. KO SYN-hAP4B1	ns	0.1703	ns	>0.9999	ns	>0.9999	ns	0.0991	ns	>0.9999	ns	>0.9999
KO V5-empty vs. KO CBh-hAP4B1	ns	0.7400	ns	0.9943	*	0.0167	ns	0.6316	ns	>0.9999	ns	0.9996
KO V5-empty vs. KO SYN-hAP4B1	ns	0.1704	ns	>0.9999	ns	>0.9999	ns	0.0992	ns	>0.9999	ns	>0.9999

KO CBh-hAP4B1 vs. KO SYN-hAP4B1	ns	0.8397	ns	0.9948	*	0.0246	ns	0.8029	ns	>0.9999	ns	0.9996
	CORTEX		HIPPOCAMPUS		BRAINSTEM		CEREBELLUM					
FIGURE 6B	Symbol	P Value	Symbol	P Value	Symbol	P Value	Symbol	P Value				
WT vs. KO untreated	****	<0.0001	****	<0.0001	****	<0.0001	****	<0.0001				
WT vs. KO V5_control	****	<0.0001	****	<0.0001	****	<0.0001	****	<0.0001				
WT vs. KO hAP4B1_low	***	0.0001	****	<0.0001	*	0.0207	***	0.0001				
WT vs. KO hAP4B1_mid	**	0.0083	*	0.0113	ns	0.1093	ns	0.0877				
WT vs. KO hAP4B1_high	ns	0.2407	ns	0.2884	ns	0.8943	ns	0.4531				
KO untreated vs. KO V5_control	ns	>0.9999	ns	0.8751	ns	0.9474	ns	>0.9999				
KO untreated vs. KO hAP4B1_low	ns	0.9241	ns	0.9998	ns	0.6592	ns	0.1345				
KO untreated vs. KO hAP4B1_mid	ns	0.2928	ns	0.1274	ns	0.2767	***	0.0010				

KO untreated vs. KO hAP4B1_high	*	0.0117	**	0.0029	**	0.0051	****	<0.0001				
KO V5_control vs. KO hAP4B1_low	ns	0.8525	ns	0.7342	ns	0.1638	ns	0.1030				
KO V5_control vs. KO hAP4B1_mid	ns	0.2062	**	0.0055	*	0.0349	***	0.0006				
KO V5_control vs. KO hAP4B1_high	**	0.0064	****	<0.0001	***	0.0002	****	<0.0001				
KO hAP4B1_low vs. KO hAP4B1_mid	ns	0.8700	ns	0.2265	ns	0.9889	ns	0.5070				
KO hAP4B1_low vs. KO hAP4B1_high	ns	0.1585	**	0.0074	ns	0.2717	ns	0.0631				
KO hAP4B1_mid vs. KO hAP4B1_high	ns	0.7886	ns	0.7872	ns	0.6527	ns	0.9366				
	BRAINSTEM		CEREBELLUM		CEREBRUM		LIVER					
FIGURE 6C	Symbol	P Value	Symbol	P Value	Symbol	P Value	Symbol	P Value				
WT vs. KO V5-control	ns	>0.9999	ns	>0.9999	ns	>0.9999	ns	>0.9999				
WT vs. KO low-dose	ns	0.9997	ns	0.9777	ns	0.9786	ns	0.5444				

WT vs. KO mid-dose	ns	0.9862	ns	0.8468	ns	0.7408	ns	>0.9999				
WT vs. KO high-dose	ns	0.4411	**	0.0066	ns	0.9490	ns	0.2376				
KO V5-control vs. KO low-dose	ns	0.9997	ns	0.9783	ns	0.9866	ns	0.5452				
KO V5-control vs. KO mid-dose	ns	0.9862	ns	0.8488	ns	0.7789	ns	>0.9999				
KO V5-control vs. KO high-dose	ns	0.4413	**	0.0067	ns	0.9639	ns	0.2381				
KO low-dose vs. KO mid-dose	ns	0.9975	ns	0.9922	ns	0.9667	ns	0.5604				
KO low-dose vs. KO high-dose	ns	0.5550	*	0.0337	ns	0.9999	ns	0.9808				
KO mid-dose vs. KO high-dose	ns	0.7559	ns	0.0976	ns	0.9880	ns	0.2485				
FIGURE 6D-G	6D		6E		6F		6G					
	Symbol	P Value	Symbol	P Value	Symbol	P Value	Symbol	P Value				
WT vs. KO untreated	**	0.0013	****	<0.0001	****	<0.0001	ns	0.3070				
WT vs. KO V5-empty	***	0.0006	****	<0.0001	****	<0.0001	ns	0.9734				

WT vs. KO low-dose	*	0.0256	ns	0.7014	****	<0.0001	ns	>0.9999				
WT vs. KO mid-dose	ns	0.6493	ns	0.8100	****	<0.0001	ns	>0.9999				
WT vs. KO high-dose	ns	0.7361	ns	>0.9999	****	<0.0001	ns	>0.9999				
KO untreated vs. KO V5-empty	ns	0.9999	ns	>0.9999	ns	0.7178	ns	0.7465				
KO untreated vs. KO low-dose	ns	0.9107	ns	0.1425	ns	0.9352	ns	0.2934				
KO untreated vs. KO mid-dose	ns	0.0901	ns	0.1865	ns	0.1570	ns	0.2754				
KO untreated vs. KO high-dose	ns	0.0648	*	0.0318	ns	0.9360	ns	0.2719				
KO V5-empty vs. KO low-dose	ns	0.8022	ns	0.1218	ns	0.2089	ns	0.9566				
KO V5-empty vs. KO mid-dose	*	0.0500	ns	0.1538	ns	0.8831	ns	0.9616				
KO V5-empty vs. KO high-dose	*	0.0350	*	0.0300	ns	0.2100	ns	0.9601				
KO low-dose vs. KO mid-dose	ns	0.5364	ns	>0.9999	*	0.0204	ns	>0.9999				
KO low-dose vs. KO high-dose	ns	0.4474	ns	>0.9999	ns	>0.9999	ns	>0.9999				

KO mid-dose vs. KO high-dose	ns	>0.9999	ns	>0.9999	*	0.0205	ns	>0.9999				
FIGURE 7A	7A Symbol	7A P Value	7B Symbol	7B P Value								
KO UT vs. KO 1E3 AAV9/AP4B1	ns	0.9995	ns	0.9813								
KO UT vs. KO 1E4 AAV9/AP4B1	ns	0.6803	ns	0.9532								
KO UT vs. KO 1E5 AAV9/AP4B1	****	<0.0001	ns	0.7544								
KO UT vs. KO 1E6 AAV9/AP4B1	****	<0.0001	*	0.0237								
KO UT vs. KO 2E6 AAV9/AP4B1	****	<0.0001	***	0.0002								
KO UT vs. KO 4E6 AAV9/AP4B1	****	<0.0001	****	<0.0001								
KO UT vs. KO 8E6 AAV9/AP4B1	****	<0.0001	****	<0.0001								
KO UT vs. KO 16E6 AAV9/AP4B1	****	<0.0001	****	<0.0001								
FIGURE 8A	WBC count (28 day)		Monocyte (28 day)		Lymphocyte (28 day)		WBC count (1 year)		Monocyte (1 year)		Lymphocyte (1 year)	

	Symbol	P Value	Symbol	P Value	Symbol	P Value	Symbol	P Value	Symbol	P Value	Symbol	P Value
Untreated vs. V5-empty	ns	0.9339	ns	0.7299	ns	0.9878	ns	0.7179	ns	0.9998	ns	0.9175
Untreated vs. CBh-hAP4B1	**	0.0014	ns	0.0503	**	0.0028	ns	0.7463	ns	0.9985	ns	0.9012
Untreated vs. SYN-hAP4B1	ns	0.996	ns	0.9844	ns	0.9996	ns	0.3764	ns	0.9975	ns	0.5776
Untreated vs. CBh-mAP4B1	ns	0.9632	ns	0.314	ns	0.993	ns	0.8249	ns	0.9995	ns	0.9417
V5-empty vs. CBh-hAP4B1	*	0.0254	ns	0.6823	*	0.0212	ns	>0.9999	ns	>0.9999	ns	>0.9999
V5-empty vs. SYN-hAP4B1	ns	0.9992	ns	0.9896	ns	0.9998	ns	0.9673	ns	>0.9999	ns	0.9917
V5-empty vs. CBh-mAP4B1	ns	>0.9999	ns	0.9922	ns	>0.9999	ns	0.9996	ns	>0.9999	ns	>0.9999
CBh-hAP4B1 vs. SYN-hAP4B1	**	0.0077	ns	0.2576	**	0.0086	ns	0.9578	ns	>0.9999	ns	0.9942
CBh-hAP4B1 vs. CBh-mAP4B1	*	0.0184	ns	0.9686	*	0.0174	ns	0.9999	ns	>0.9999	ns	>0.9999
SYN-hAP4B1 vs. CBh-mAP4B1	ns	0.9999	ns	0.7856	ns	>0.9999	ns	0.9195	ns	>0.9999	ns	0.9854
FIGURE EV1	EV1 B		EV1 C									

	Symbol	P value	Symbol	P value								
WT vs UT	ns	0.9073	***	0.0005								
WT vs MOI 5	ns	0.9971	ns	0.0562								
WT vs MOI 10	ns	0.9864	ns	0.9112								
WT vs MOI 20	ns	0.7331	ns	0.9582								
UT vs MOI 5	ns	0.9826	*	0.0443								
UT vs MOI 10	ns	0.6721	**	0.0014								
UT vs MOI 20	ns	0.2957	**	0.0011								
MOI 5 vs MOI 10	ns	0.9186	ns	0.1996								
MOI 5 vs MOI 20	ns	0.5495	ns	0.1560								
MOI 10 vs MOI 20	ns	0.9411	ns	0.9998								
FIGURE EV2	Symbol	P value		FIGURE EV4		Symbol	P value					

WT vs. UT	****	<0.0001		WT vs. UT	****	<0.0001					
WT vs. V5	****	<0.0001		WT vs. V5	****	<0.0001					
WT vs. CBH	ns	0.7952		WT vs. LD	****	<0.0001					
WT vs. SYN	ns	0.6298		WT vs. MD	***	0.0007					
UT vs. V5	ns	0.2437		WT vs. HD	*	0.0126					
UT vs. CBH	****	<0.0001		UT vs. V5	ns	0.7636					
UT vs. SYN	****	<0.0001		UT vs. LD	ns	0.3213					
V5 vs. CBH	****	<0.0001		UT vs. MD	**	0.0066					
V5 vs. SYN	****	<0.0001		UT vs. HD	***	0.0004					
CBH vs. SYN	ns	0.9978		V5 vs. LD	ns	0.9735					
				V5 vs. MD	ns	0.1471					

				V5 vs. HD	*	0.0126					
				LD vs. MD	ns	0.4929					
				LD vs. HD	ns	0.0740					
				MD vs. HD	ns	0.8836					
