# Supplementary figures and images for: Pre-clinical development of AP4B1 gene replacement therapy for hereditary spastic paraplegia type 47
Source: EMBO Mol Med. 2024 Oct 2;16(11):2882–917. doi: 10.1038/s44321-024-00148-5 (PMC11554807; doi:10.1038/s44321-024-00148-5)

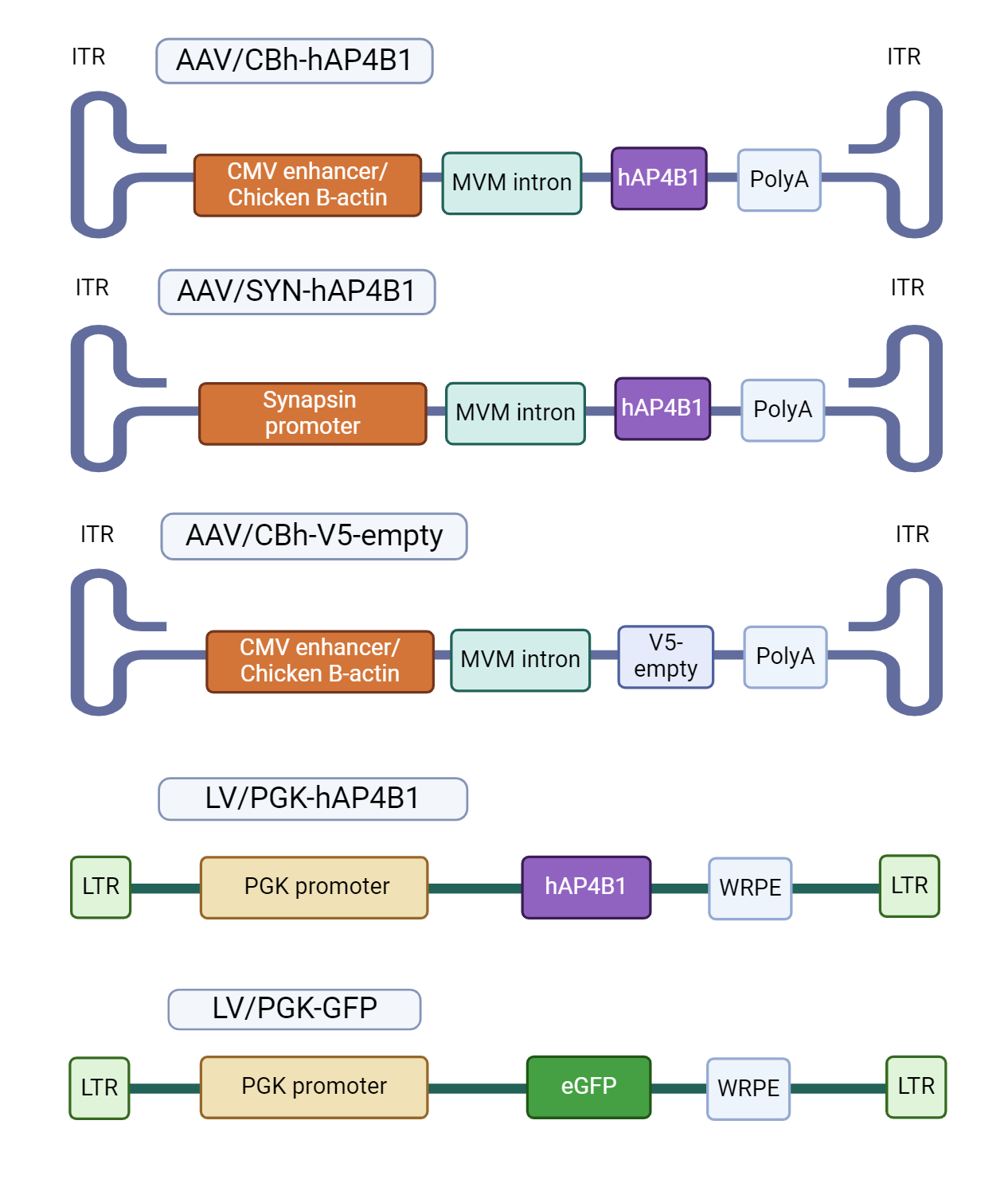

Supplement: Supplementary file 3 — Source data Fig. 1 [file 44321_2024_148_MOESM3_ESM.zip › Figure 1/A/Figure 1A.png]

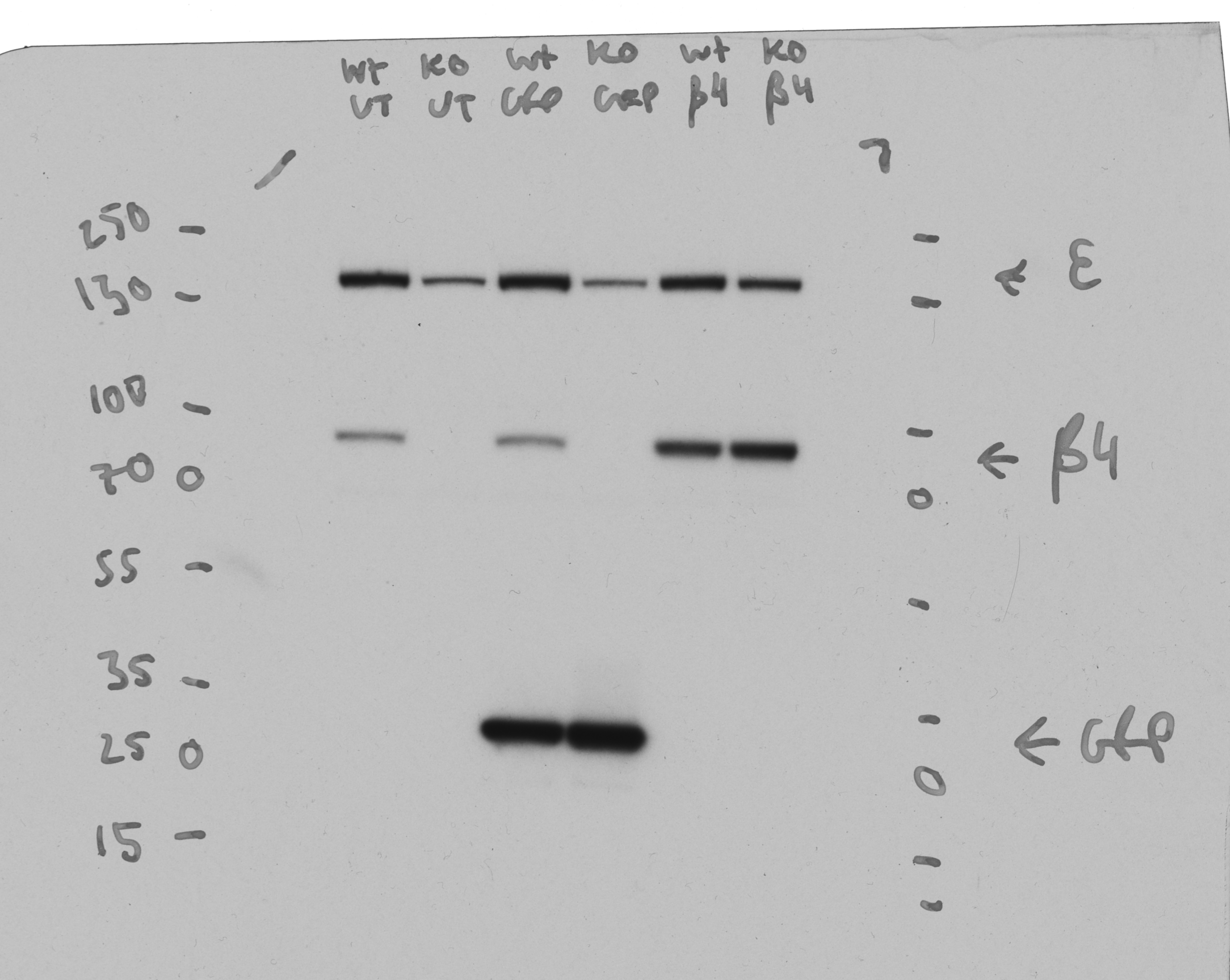

Supplement: Supplementary file 3 — Source data Fig. 1 [file 44321_2024_148_MOESM3_ESM.zip › Figure 1/B/180125_Scarrott_AP4B1001.tif]

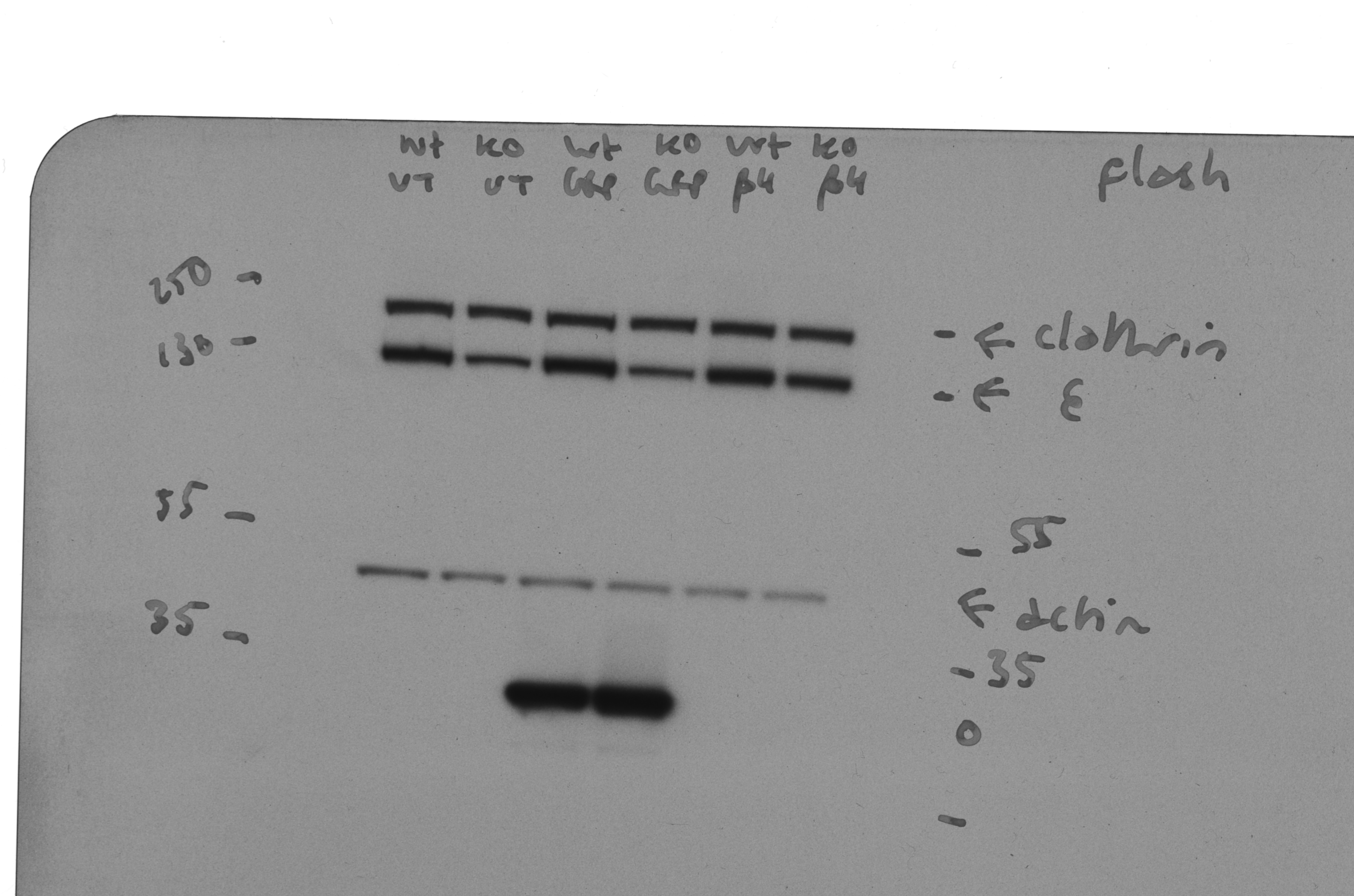

Supplement: Supplementary file 3 — Source data Fig. 1 [file 44321_2024_148_MOESM3_ESM.zip › Figure 1/B/180130_Scarrott_Clathrin001.tif]

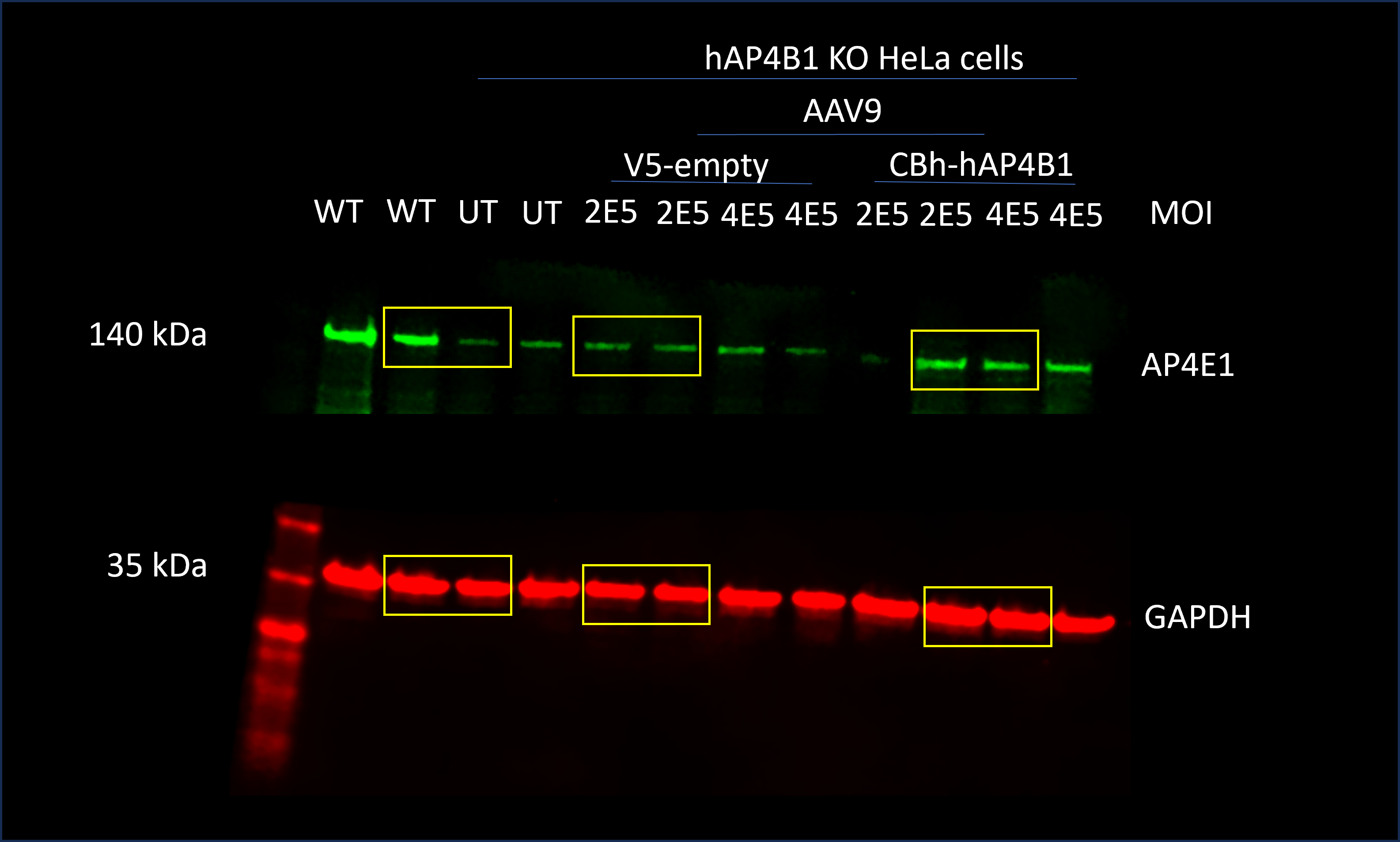

Supplement: Supplementary file 3 — Source data Fig. 1 [file 44321_2024_148_MOESM3_ESM.zip › Figure 1/D/WB AP4E1 and GAPDH - AAV9 transduction HeLa cells v2.png]

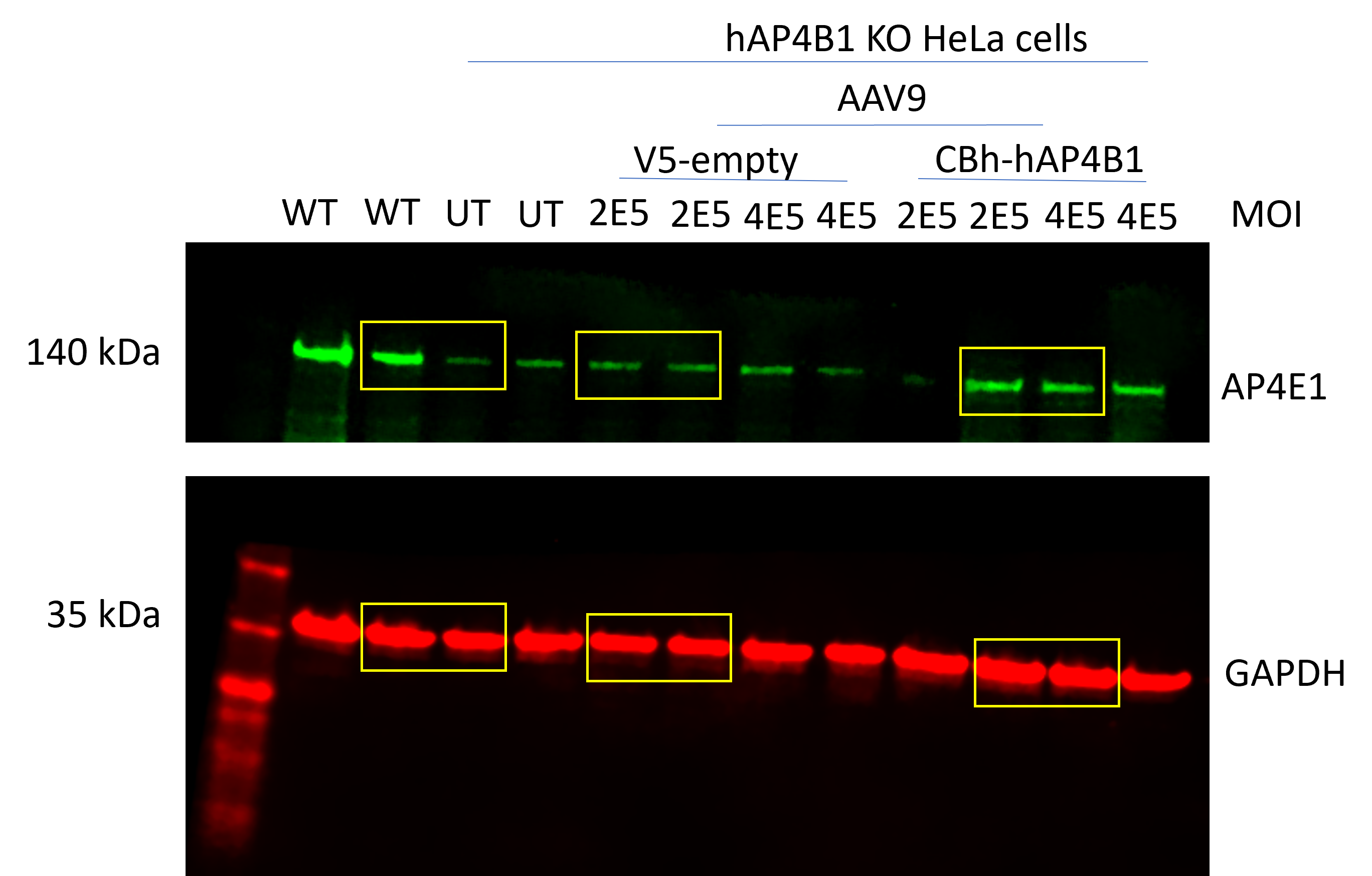

Supplement: Supplementary file 3 — Source data Fig. 1 [file 44321_2024_148_MOESM3_ESM.zip › Figure 1/D/WB AP4E1 and GAPDH - AAV9 transduction HeLa cells.png]

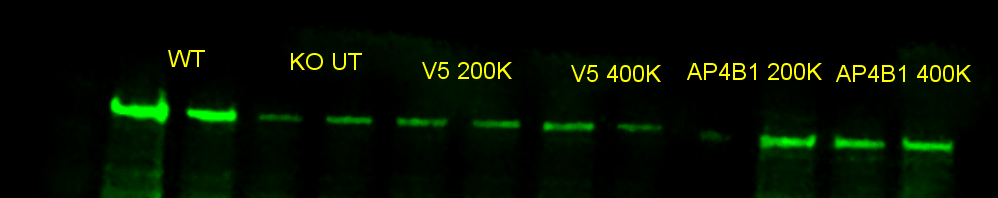

Supplement: Supplementary file 3 — Source data Fig. 1 [file 44321_2024_148_MOESM3_ESM.zip › Figure 1/E/Transduction 1 - ap4e1.tif]

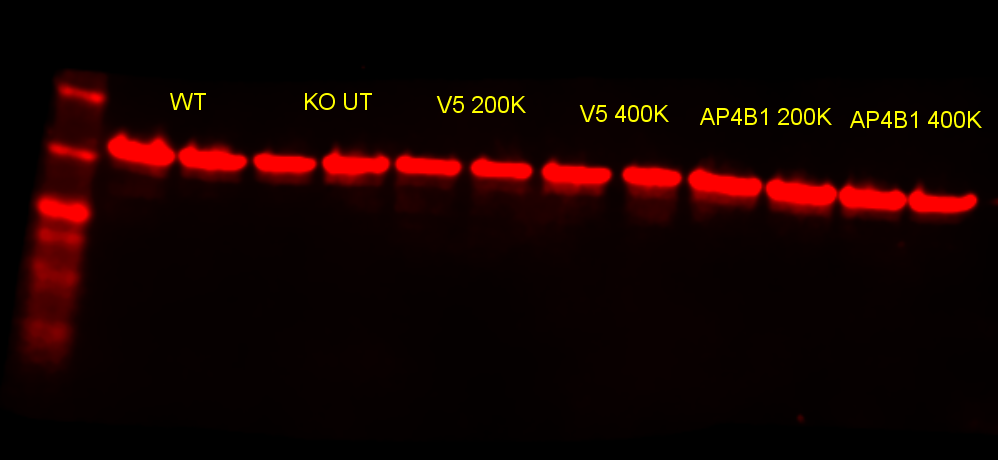

Supplement: Supplementary file 3 — Source data Fig. 1 [file 44321_2024_148_MOESM3_ESM.zip › Figure 1/E/Transduction 1 - gapdh.tif]

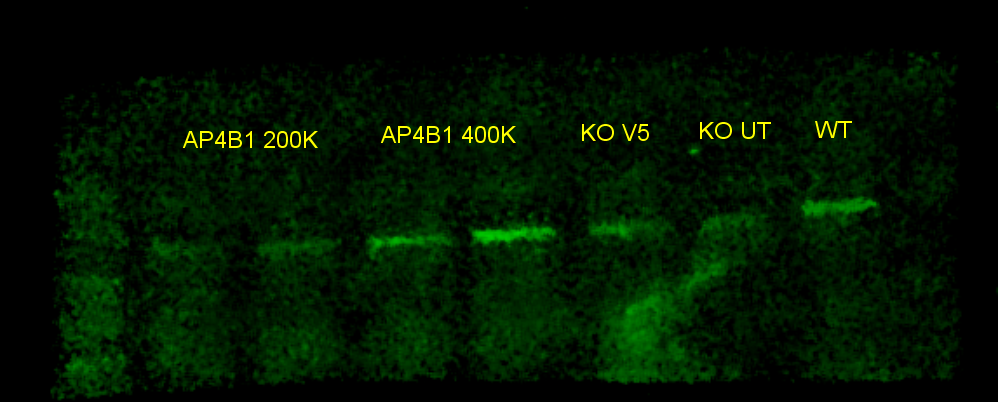

Supplement: Supplementary file 3 — Source data Fig. 1 [file 44321_2024_148_MOESM3_ESM.zip › Figure 1/E/Transduction 2 - ap4e1.tif]

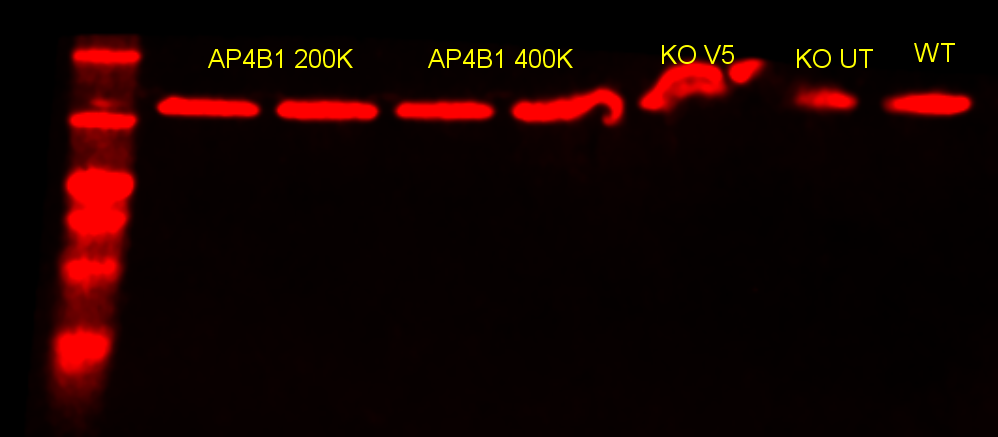

Supplement: Supplementary file 3 — Source data Fig. 1 [file 44321_2024_148_MOESM3_ESM.zip › Figure 1/E/Transduction 2 - gapdh.tif]

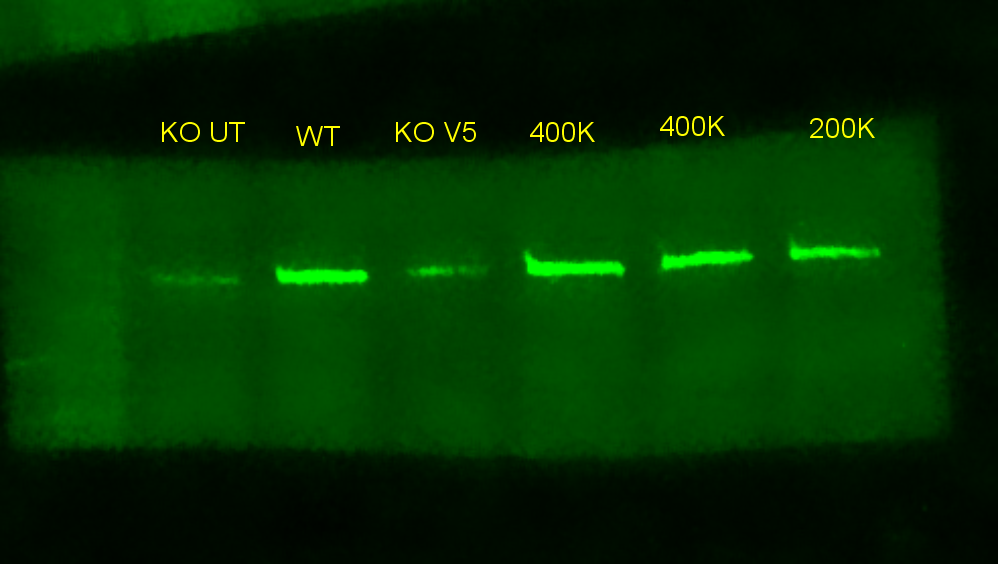

Supplement: Supplementary file 3 — Source data Fig. 1 [file 44321_2024_148_MOESM3_ESM.zip › Figure 1/E/Transduction 3 - ap4e1.tif]

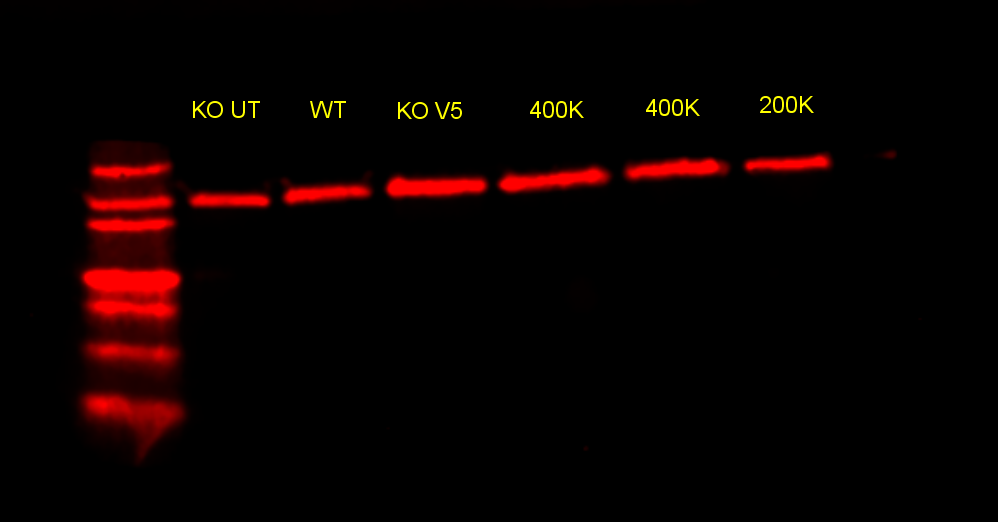

Supplement: Supplementary file 3 — Source data Fig. 1 [file 44321_2024_148_MOESM3_ESM.zip › Figure 1/E/Transduction 3 - gapdh.png]

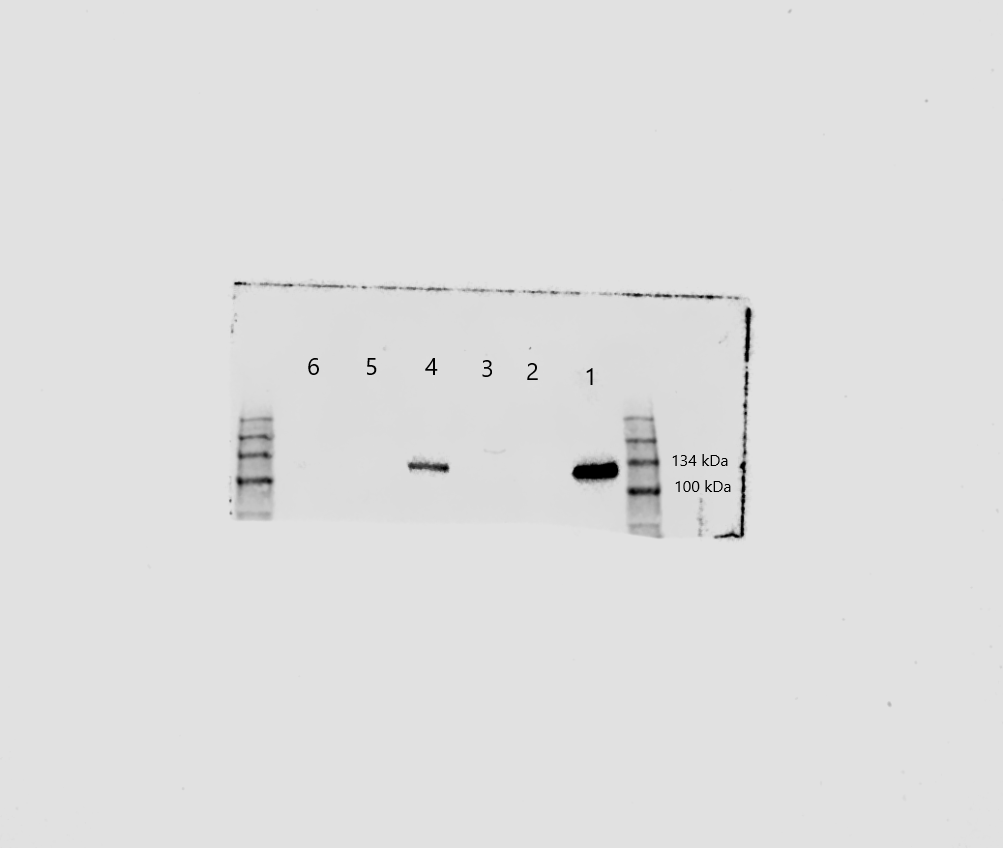

Supplement: Supplementary file 5 — Source data Fig. 3 [file 44321_2024_148_MOESM5_ESM.zip › Figure 3/G-L/G - Cerebrum/n1 - figure images/AP4E1_Cerebrum_JAC-01_1st set.tif]

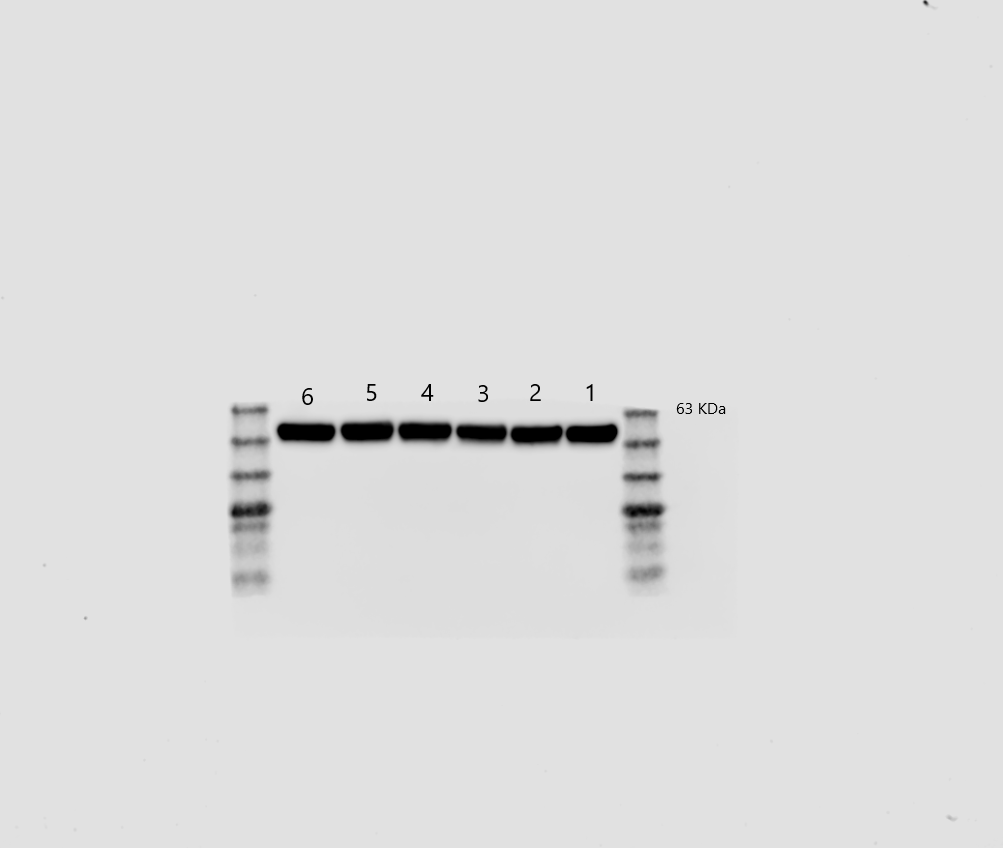

Supplement: Supplementary file 5 — Source data Fig. 3 [file 44321_2024_148_MOESM5_ESM.zip › Figure 3/G-L/G - Cerebrum/n1 - figure images/Tub_for_AP4E1_Cerebrum_JAC-01_1st set.tif]

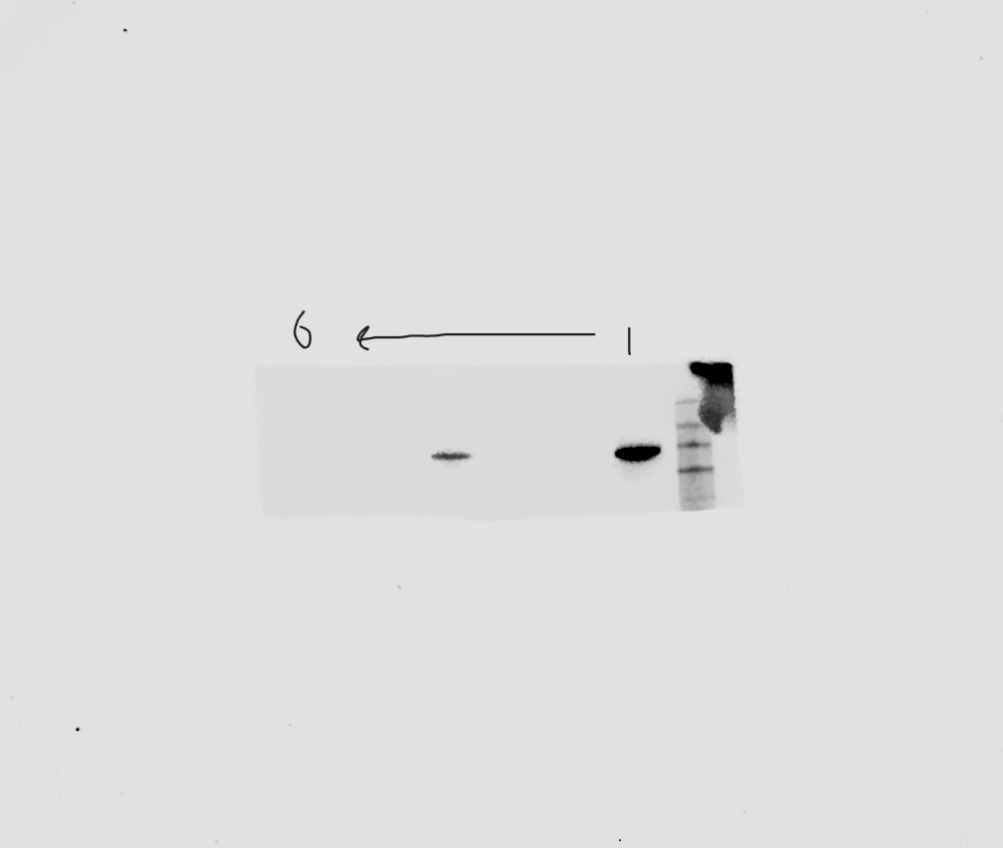

Supplement: Supplementary file 5 — Source data Fig. 3 [file 44321_2024_148_MOESM5_ESM.zip › Figure 3/G-L/G - Cerebrum/n2/AP4E1_Cerebrum_JAC-01_2nd set.tif]

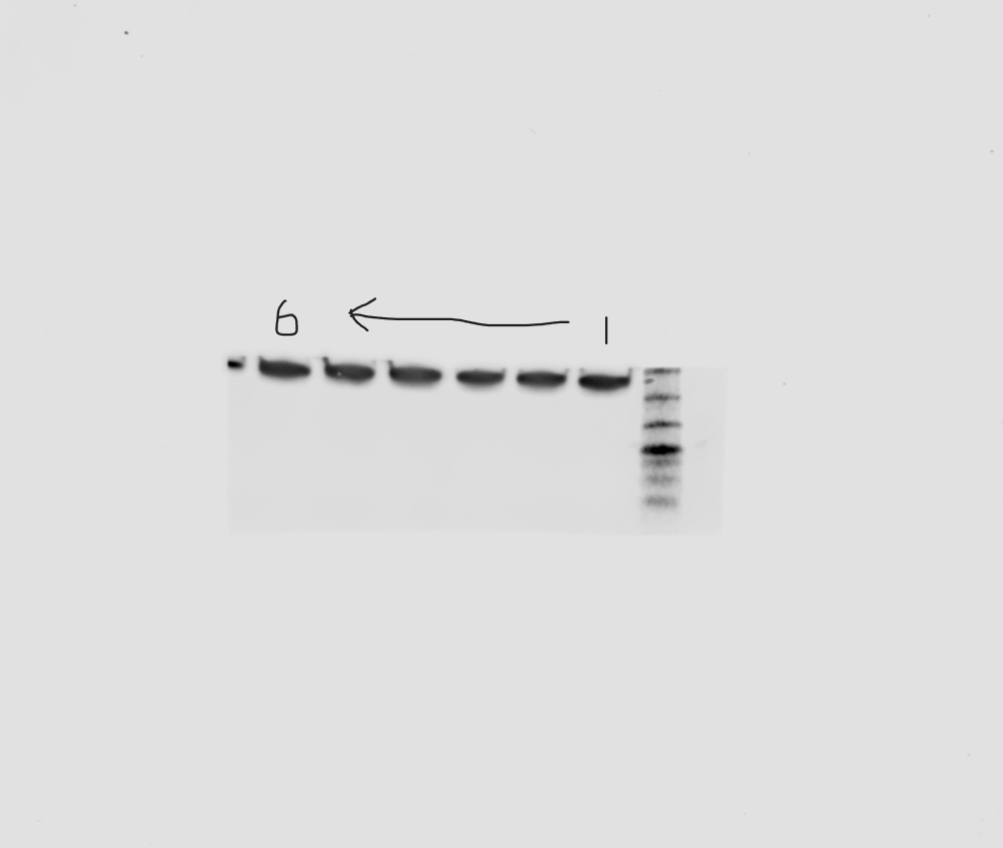

Supplement: Supplementary file 5 — Source data Fig. 3 [file 44321_2024_148_MOESM5_ESM.zip › Figure 3/G-L/G - Cerebrum/n2/Tub_for_AP4E1_Cerebrum_JAC-01_2nd set.tif]

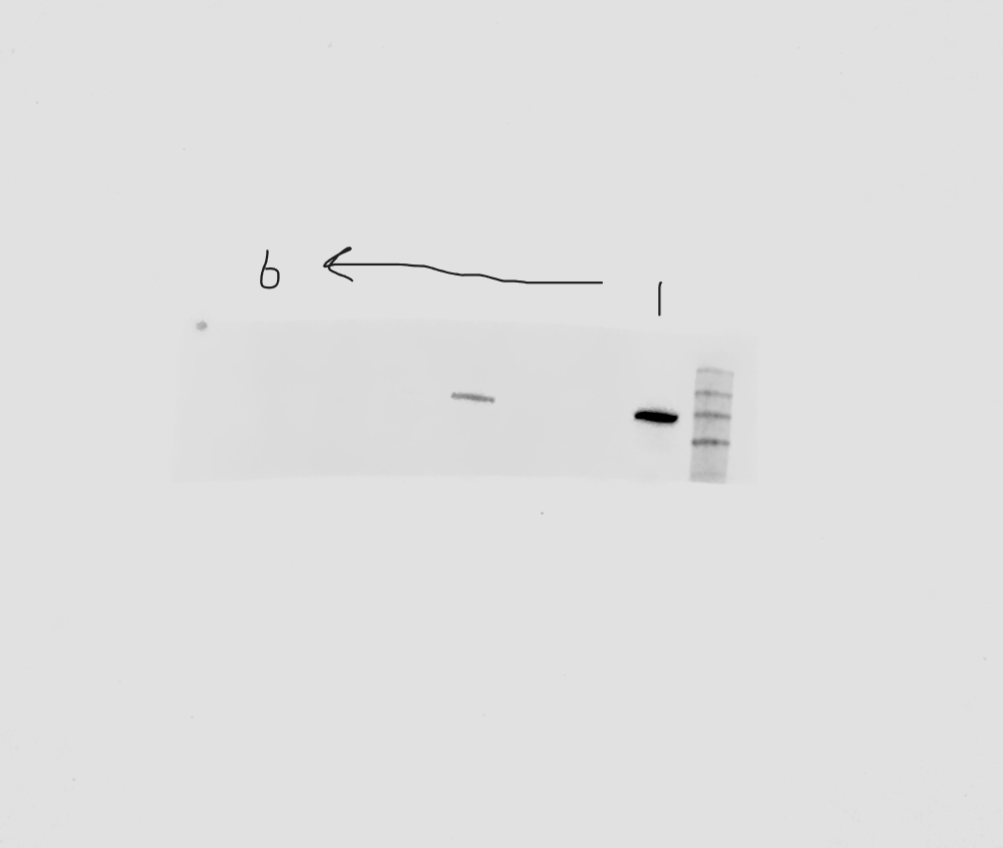

Supplement: Supplementary file 5 — Source data Fig. 3 [file 44321_2024_148_MOESM5_ESM.zip › Figure 3/G-L/G - Cerebrum/n3/AP4E1_Cerebrum_JAC-01_3rd set.tif]

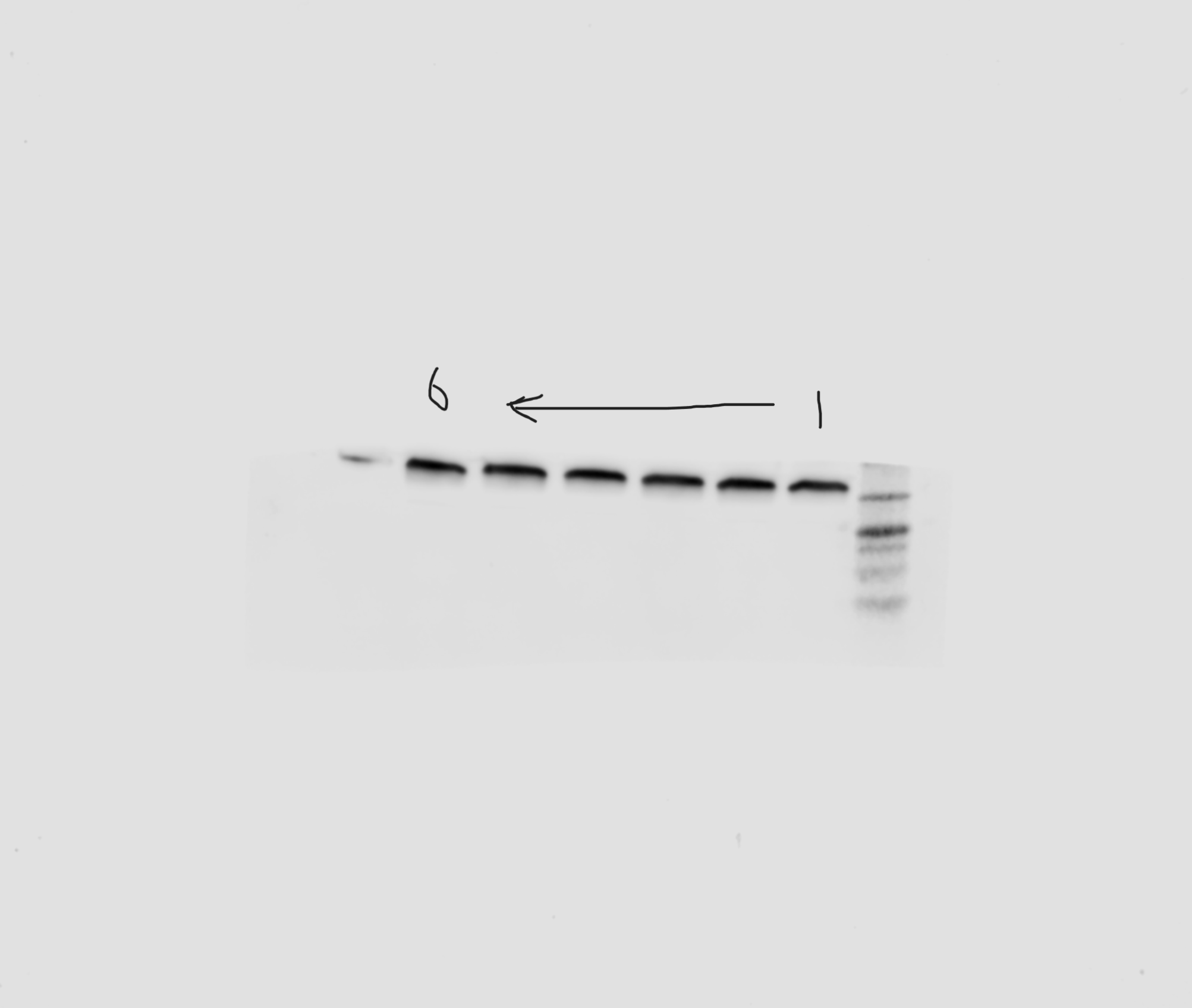

Supplement: Supplementary file 5 — Source data Fig. 3 [file 44321_2024_148_MOESM5_ESM.zip › Figure 3/G-L/G - Cerebrum/n3/GAPDH_for_AP4E1_Cerebrum_JAC-01_3rd set.tif]

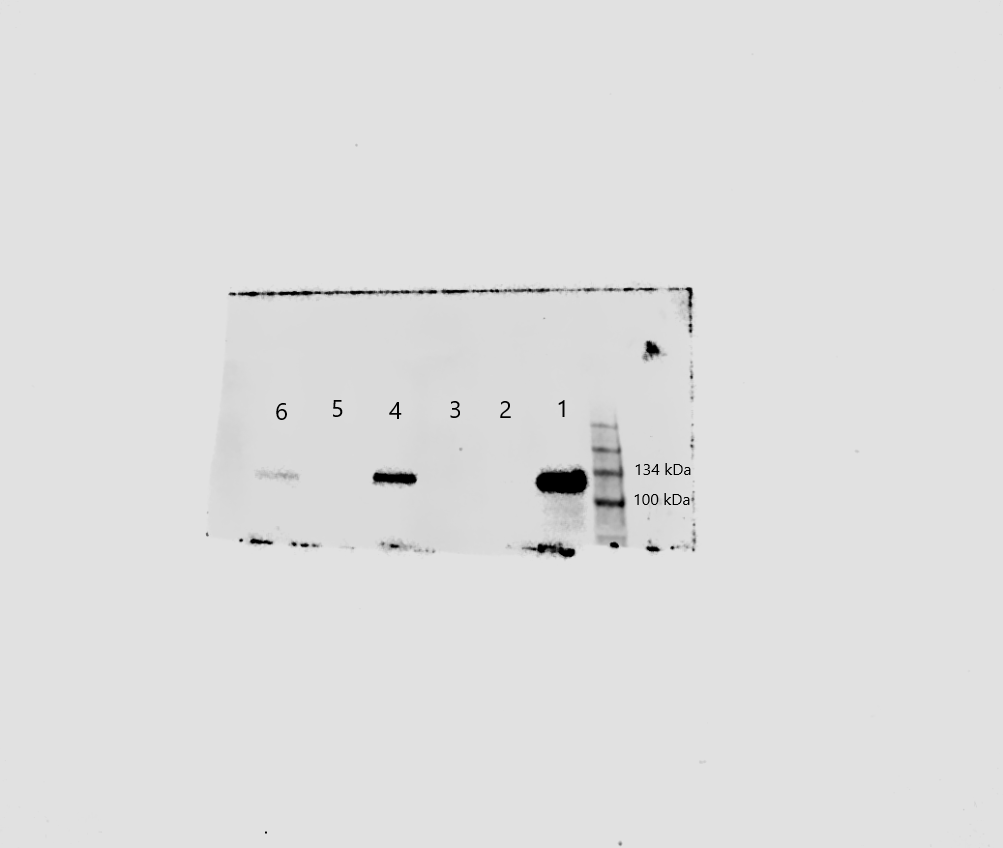

Supplement: Supplementary file 5 — Source data Fig. 3 [file 44321_2024_148_MOESM5_ESM.zip › Figure 3/G-L/I - Spinal cord/n1 - figure images/AP4E1_SC_JAC-01_1st set.tif]

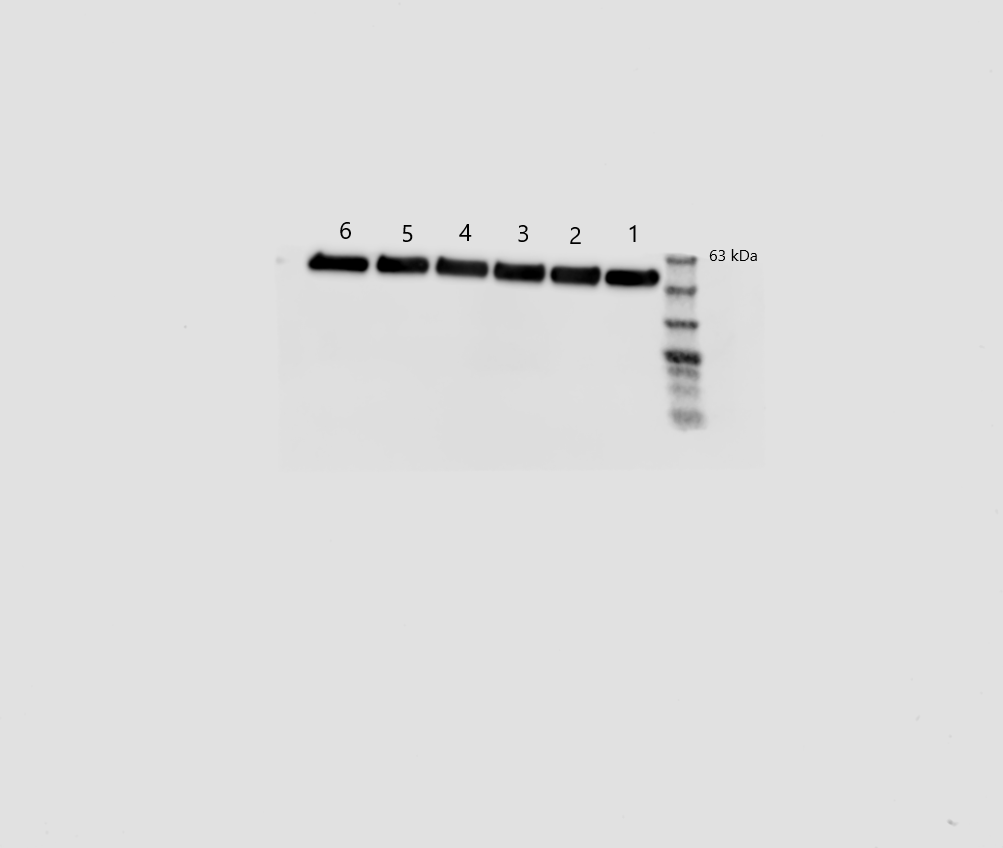

Supplement: Supplementary file 5 — Source data Fig. 3 [file 44321_2024_148_MOESM5_ESM.zip › Figure 3/G-L/I - Spinal cord/n1 - figure images/Tub_for_AP4E1_SC_JAC-01_1st set.tif]

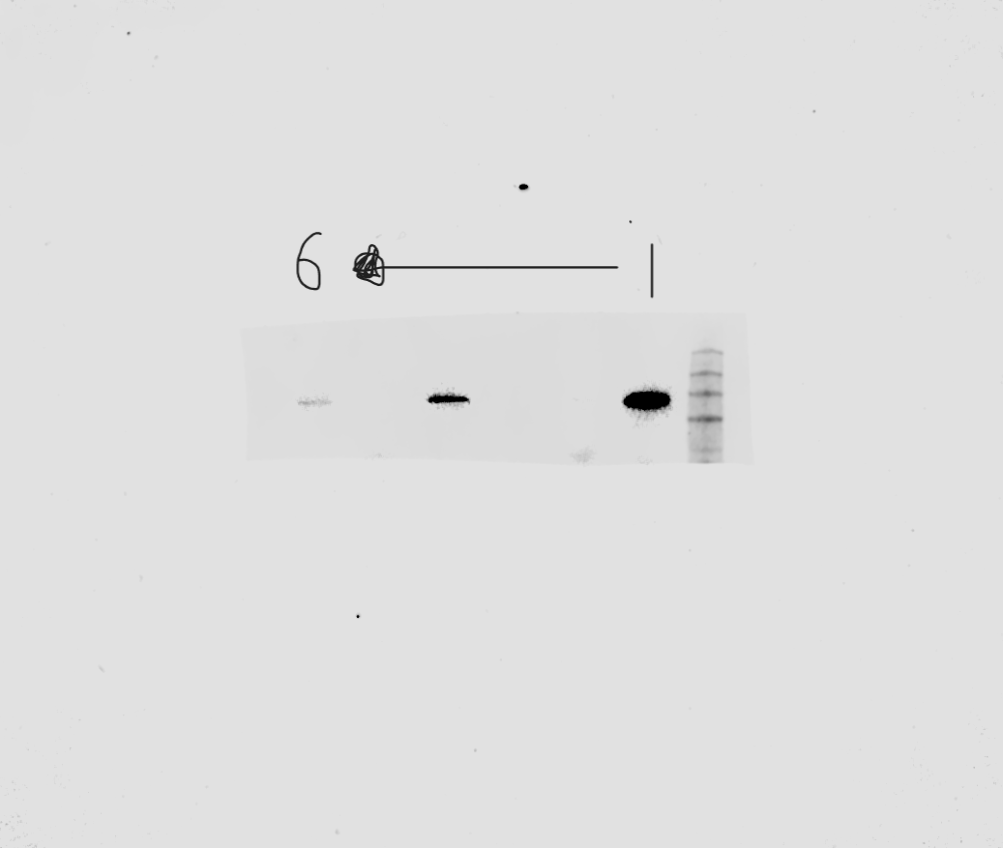

Supplement: Supplementary file 5 — Source data Fig. 3 [file 44321_2024_148_MOESM5_ESM.zip › Figure 3/G-L/I - Spinal cord/n2/AP4E1_SC_JAC-01_2nd set.tif]

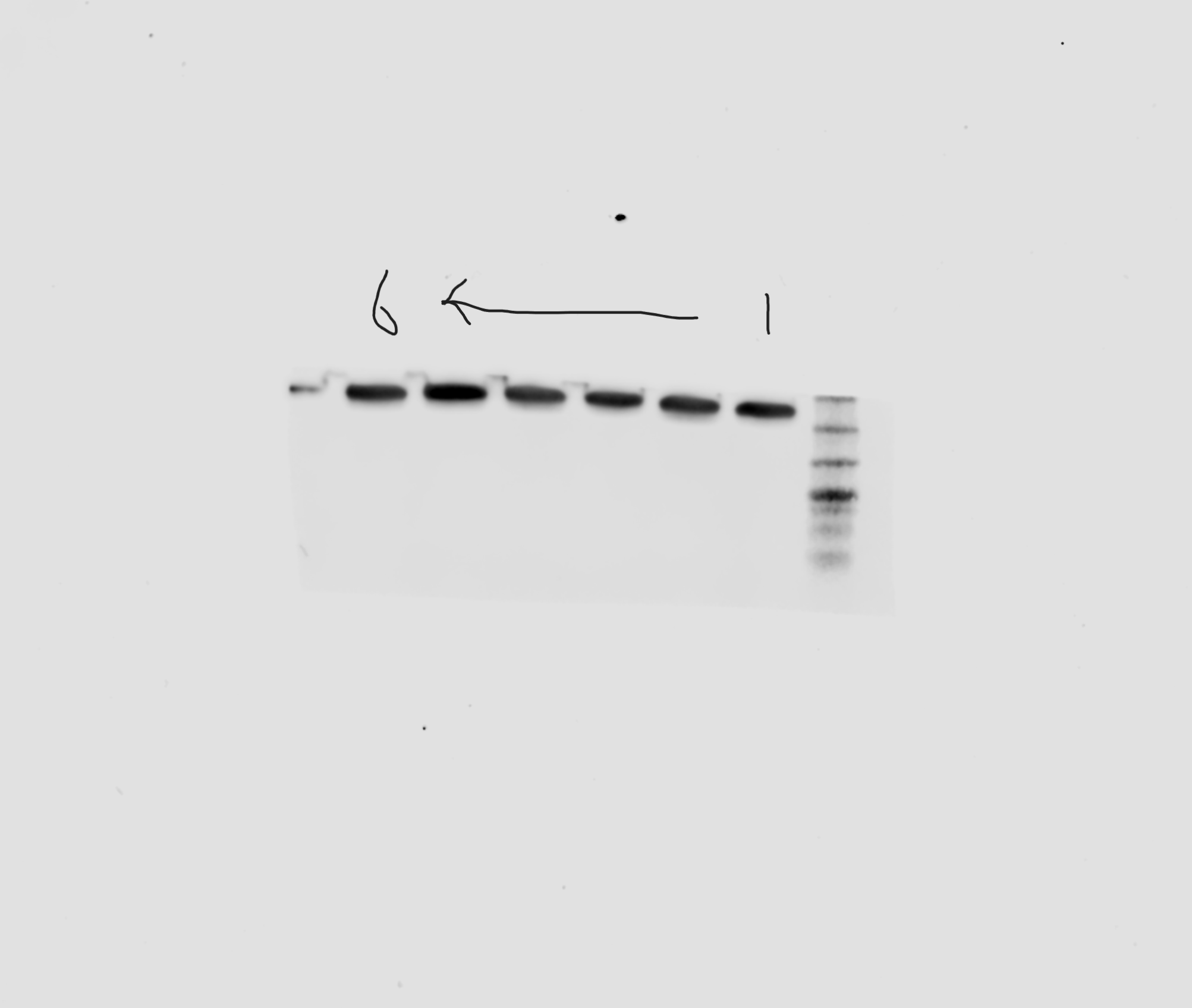

Supplement: Supplementary file 5 — Source data Fig. 3 [file 44321_2024_148_MOESM5_ESM.zip › Figure 3/G-L/I - Spinal cord/n2/Tub_for_AP4E1_SC_JAC-01_2nd set.tif]

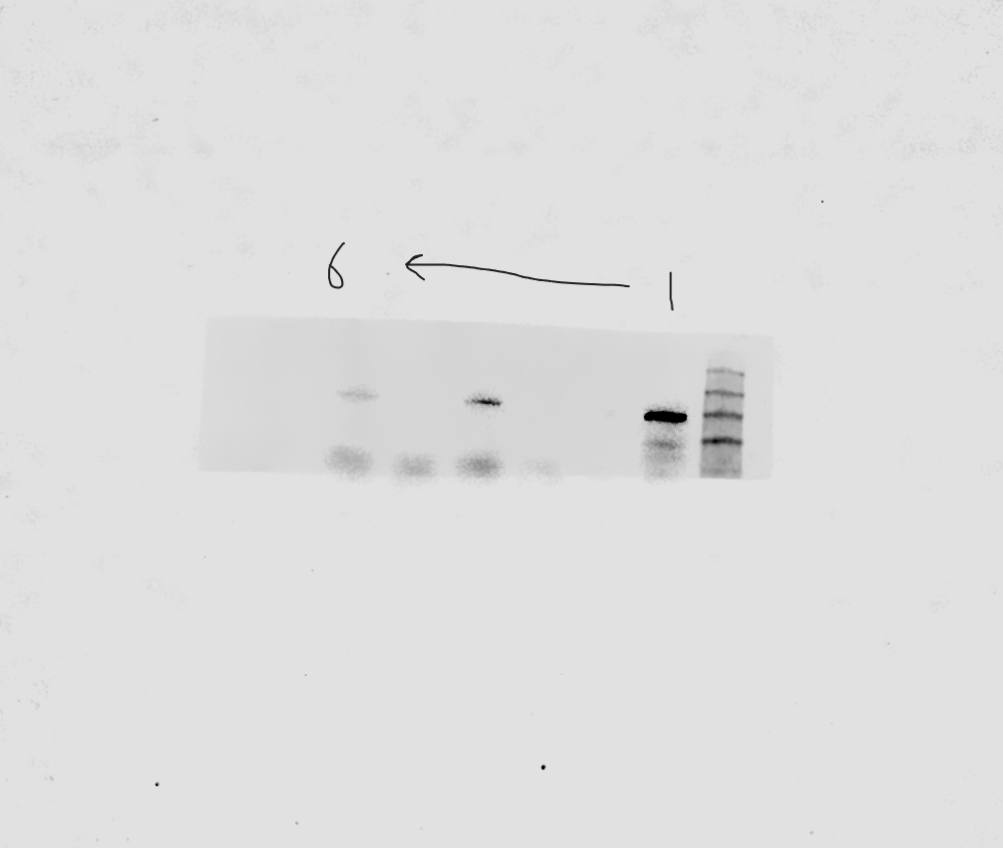

Supplement: Supplementary file 5 — Source data Fig. 3 [file 44321_2024_148_MOESM5_ESM.zip › Figure 3/G-L/I - Spinal cord/n3/AP4E1_SC_JAC-01_3rd set.tif]

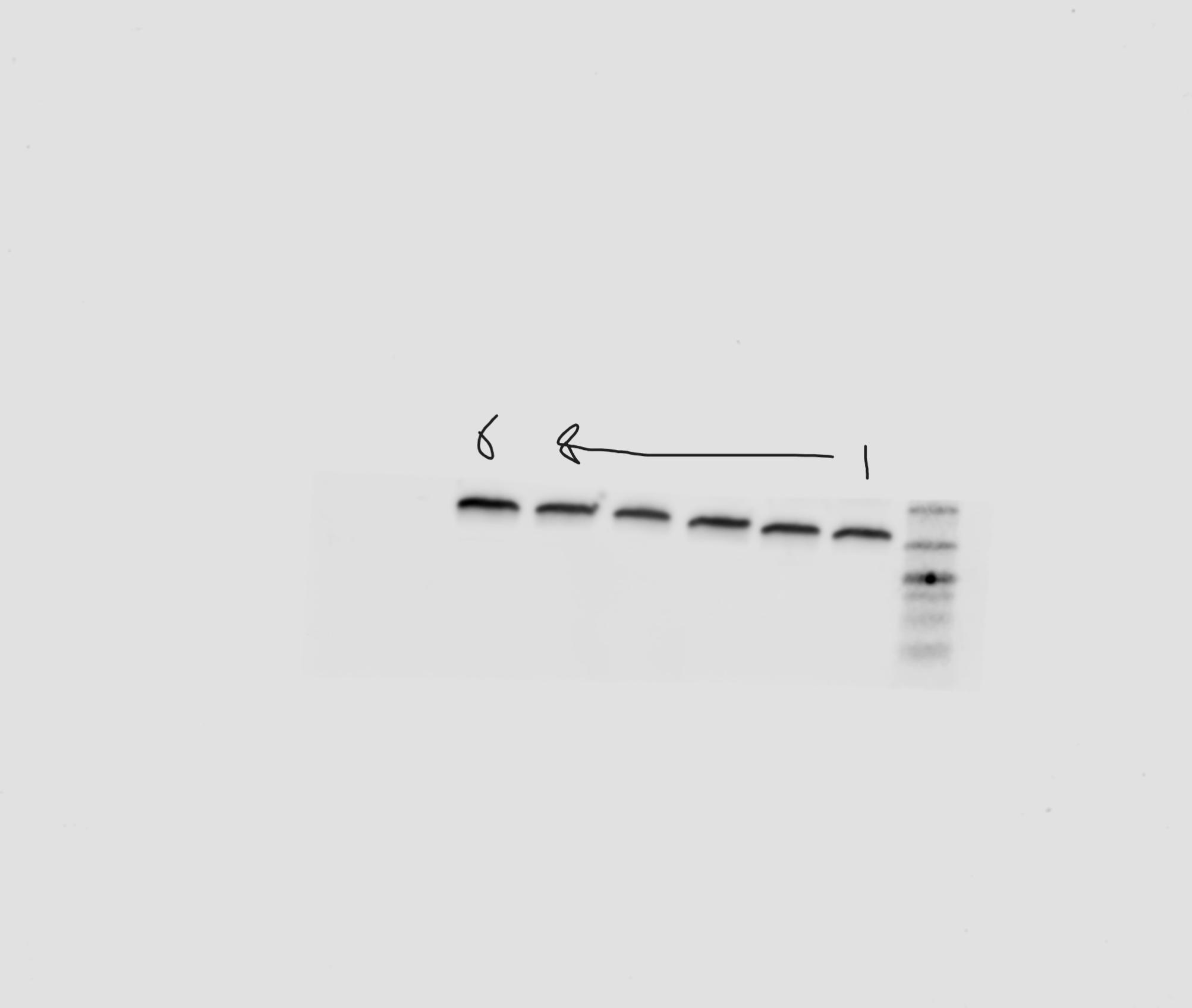

Supplement: Supplementary file 5 — Source data Fig. 3 [file 44321_2024_148_MOESM5_ESM.zip › Figure 3/G-L/I - Spinal cord/n3/GAPDH_for_AP4E1_SC_JAC-01_3rd set.tif]

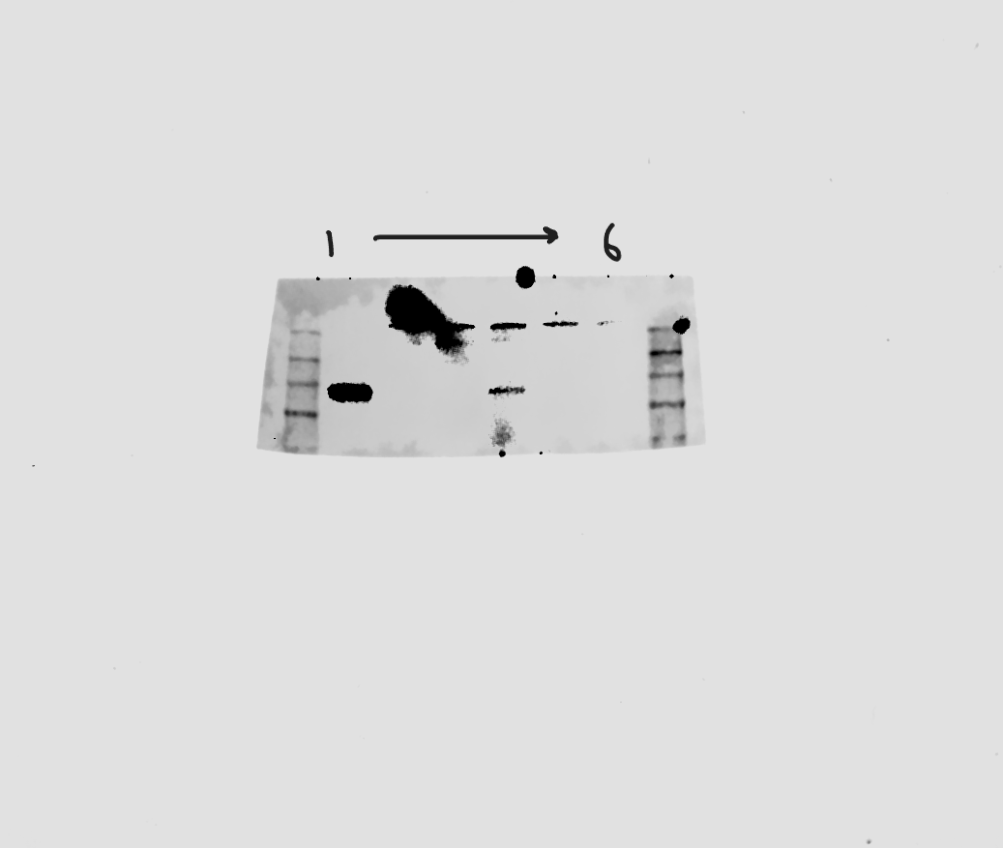

Supplement: Supplementary file 5 — Source data Fig. 3 [file 44321_2024_148_MOESM5_ESM.zip › Figure 3/G-L/K - Cerebellum/n1/AP4E1_Cereb_284224set_JAC-1_3rd set_10minExp.tif]

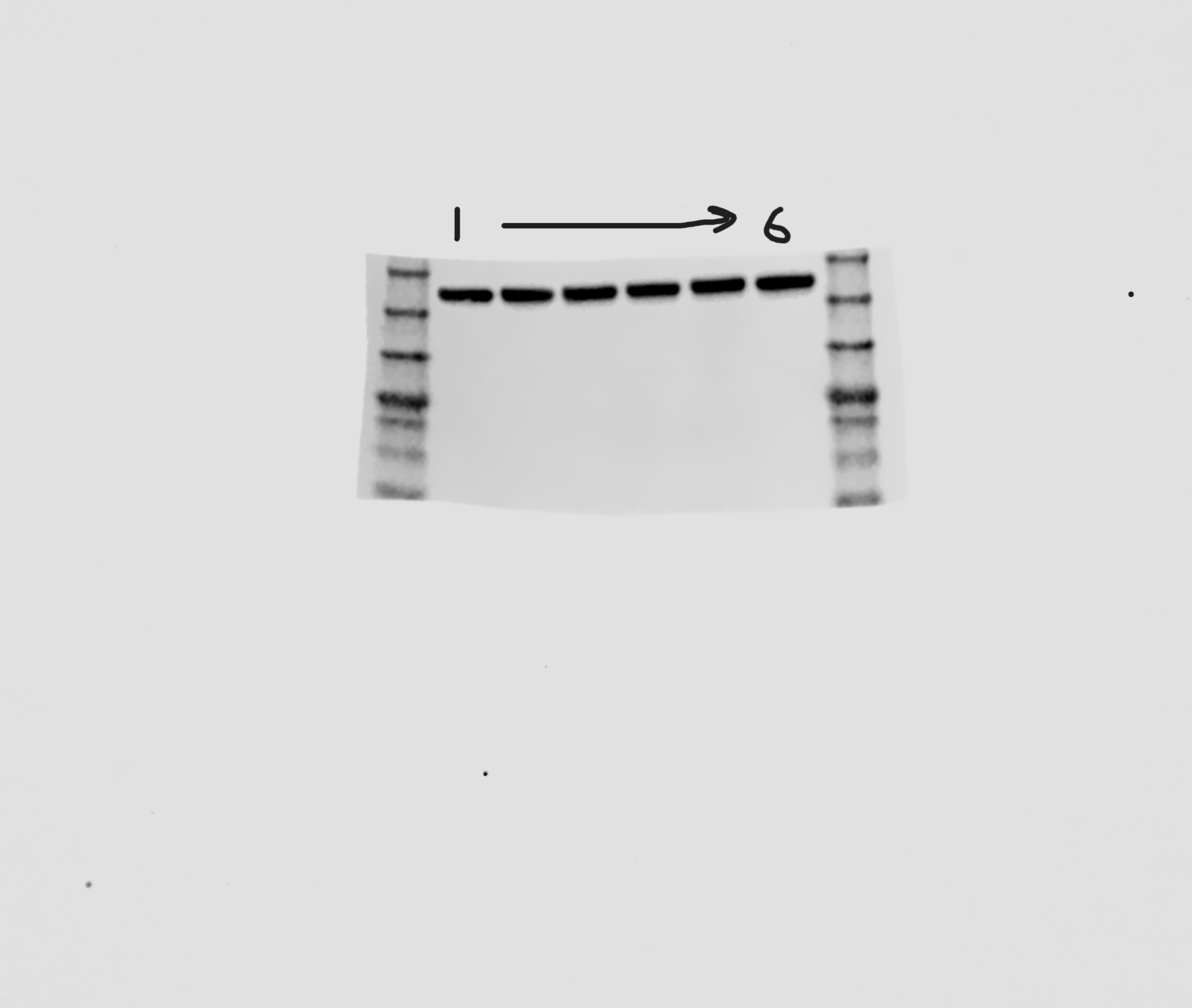

Supplement: Supplementary file 5 — Source data Fig. 3 [file 44321_2024_148_MOESM5_ESM.zip › Figure 3/G-L/K - Cerebellum/n1/Tub_for_AP4E1_Cereb_284224set_JAC-01_3rd set.tif]

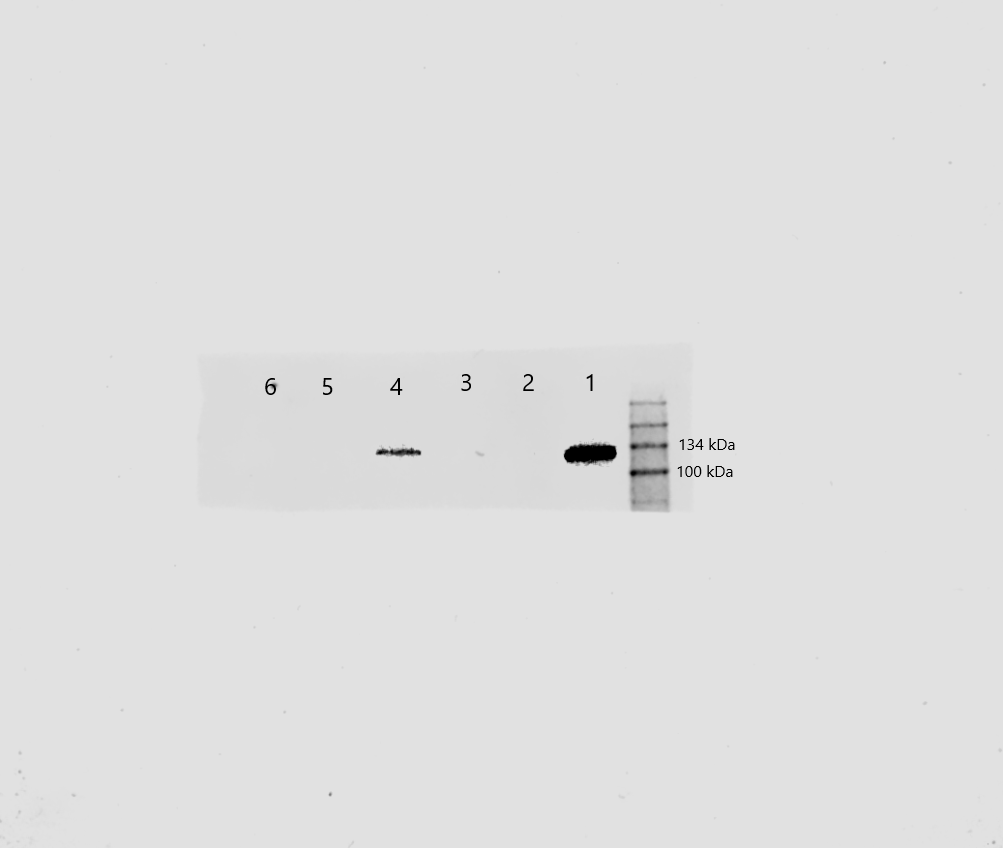

Supplement: Supplementary file 5 — Source data Fig. 3 [file 44321_2024_148_MOESM5_ESM.zip › Figure 3/G-L/K - Cerebellum/n2 -figure images/AP4E1_Cereb_JAC-01_2nd set.tif]

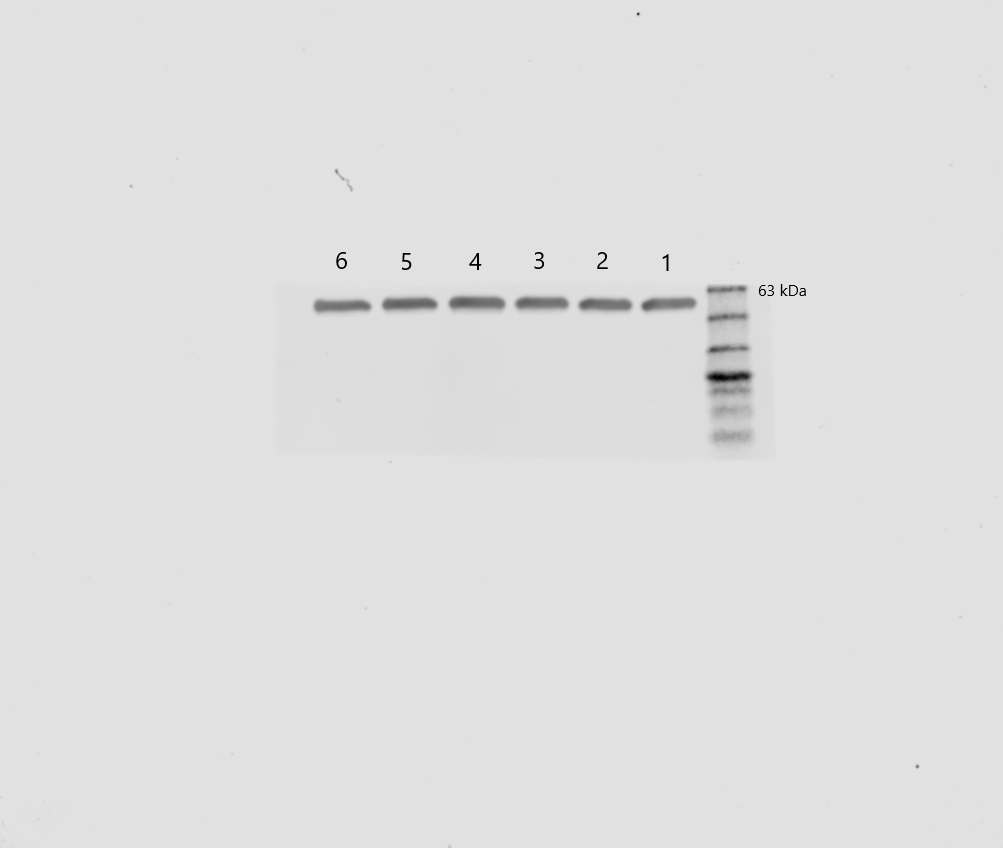

Supplement: Supplementary file 5 — Source data Fig. 3 [file 44321_2024_148_MOESM5_ESM.zip › Figure 3/G-L/K - Cerebellum/n2 -figure images/Tub_for_AP4E1_Cereb_JAC-01_2nd set.tif]

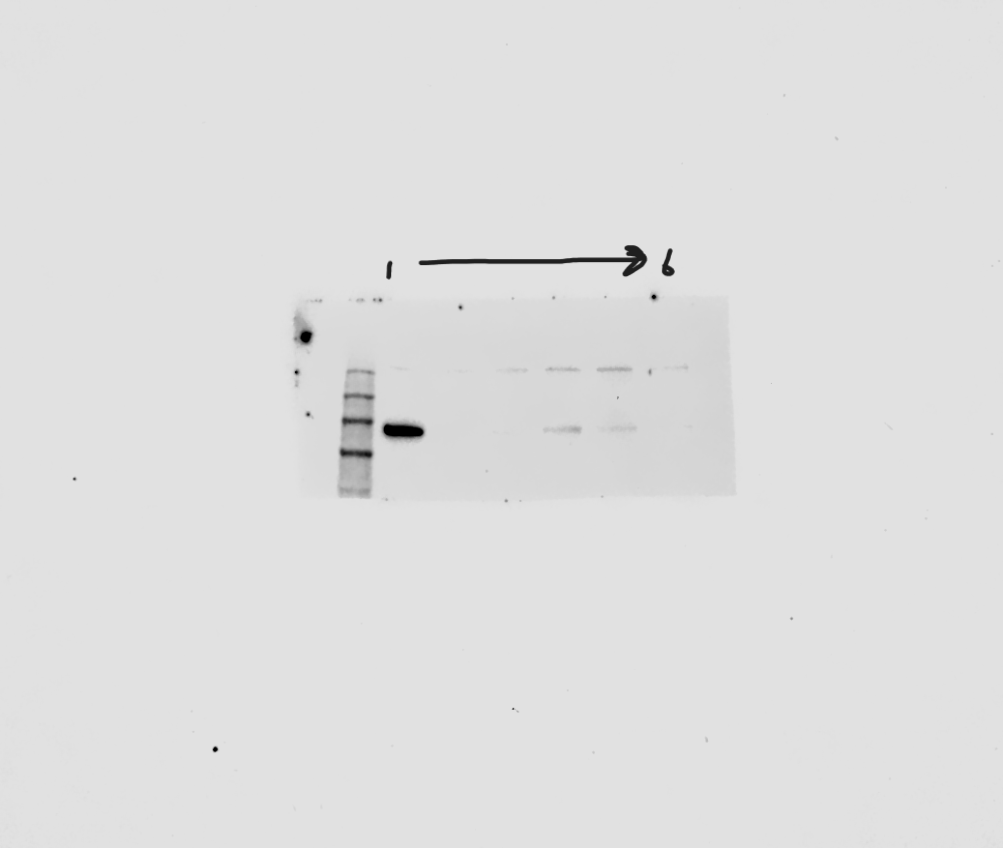

Supplement: Supplementary file 5 — Source data Fig. 3 [file 44321_2024_148_MOESM5_ESM.zip › Figure 3/G-L/K - Cerebellum/n3/AP4E1_Cereb_284229set_JAC-01_4th set.tif]

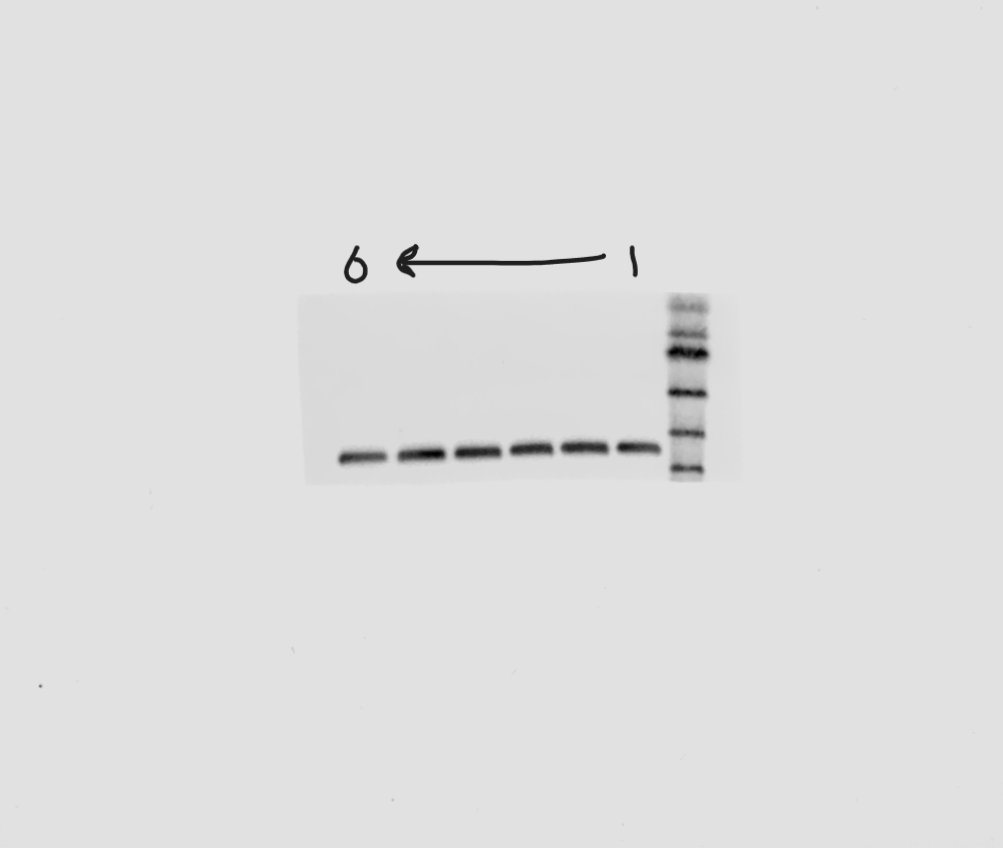

Supplement: Supplementary file 5 — Source data Fig. 3 [file 44321_2024_148_MOESM5_ESM.zip › Figure 3/G-L/K - Cerebellum/n3/Tub_for_AP4E1_Cereb_284229set_JAC-01_4th set.tif]

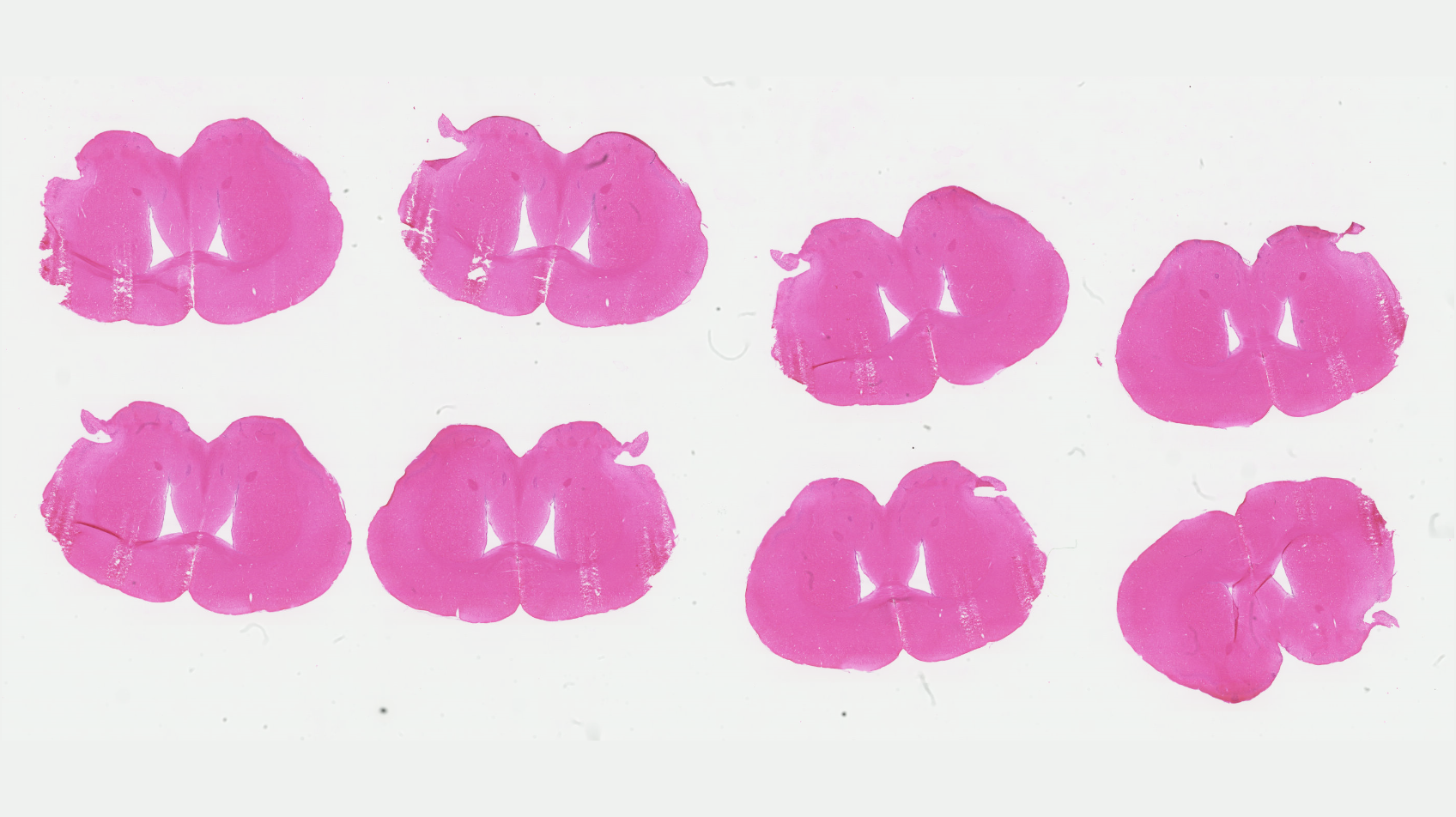

Supplement: Supplementary file 7 — Source data Fig. 5 [file 44321_2024_148_MOESM7_ESM.zip › Figure 5/A-B/Figure A/corpus callosum/CBh1 - 324362.tif]

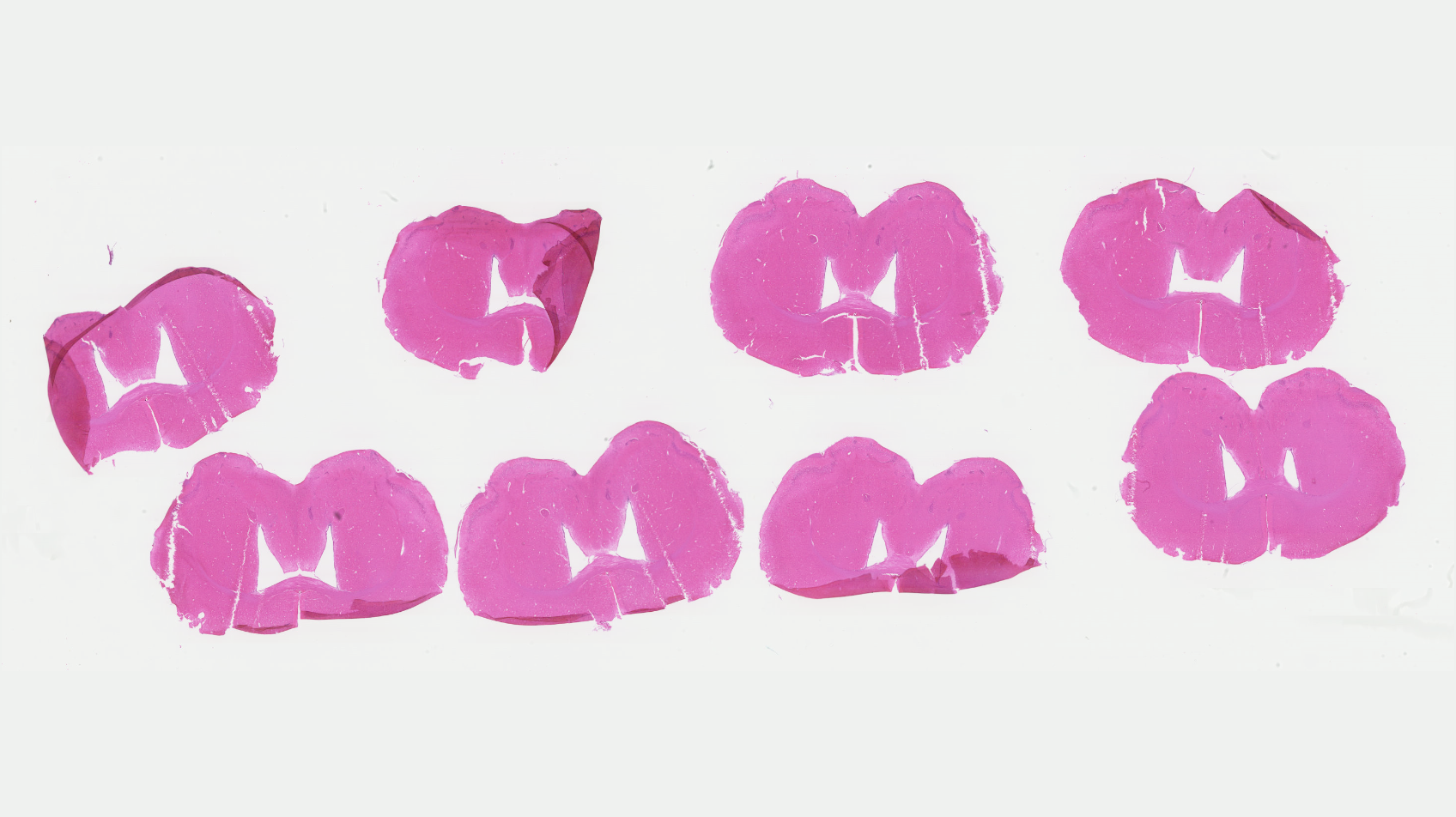

Supplement: Supplementary file 7 — Source data Fig. 5 [file 44321_2024_148_MOESM7_ESM.zip › Figure 5/A-B/Figure A/corpus callosum/CBH2 -324362.tif]

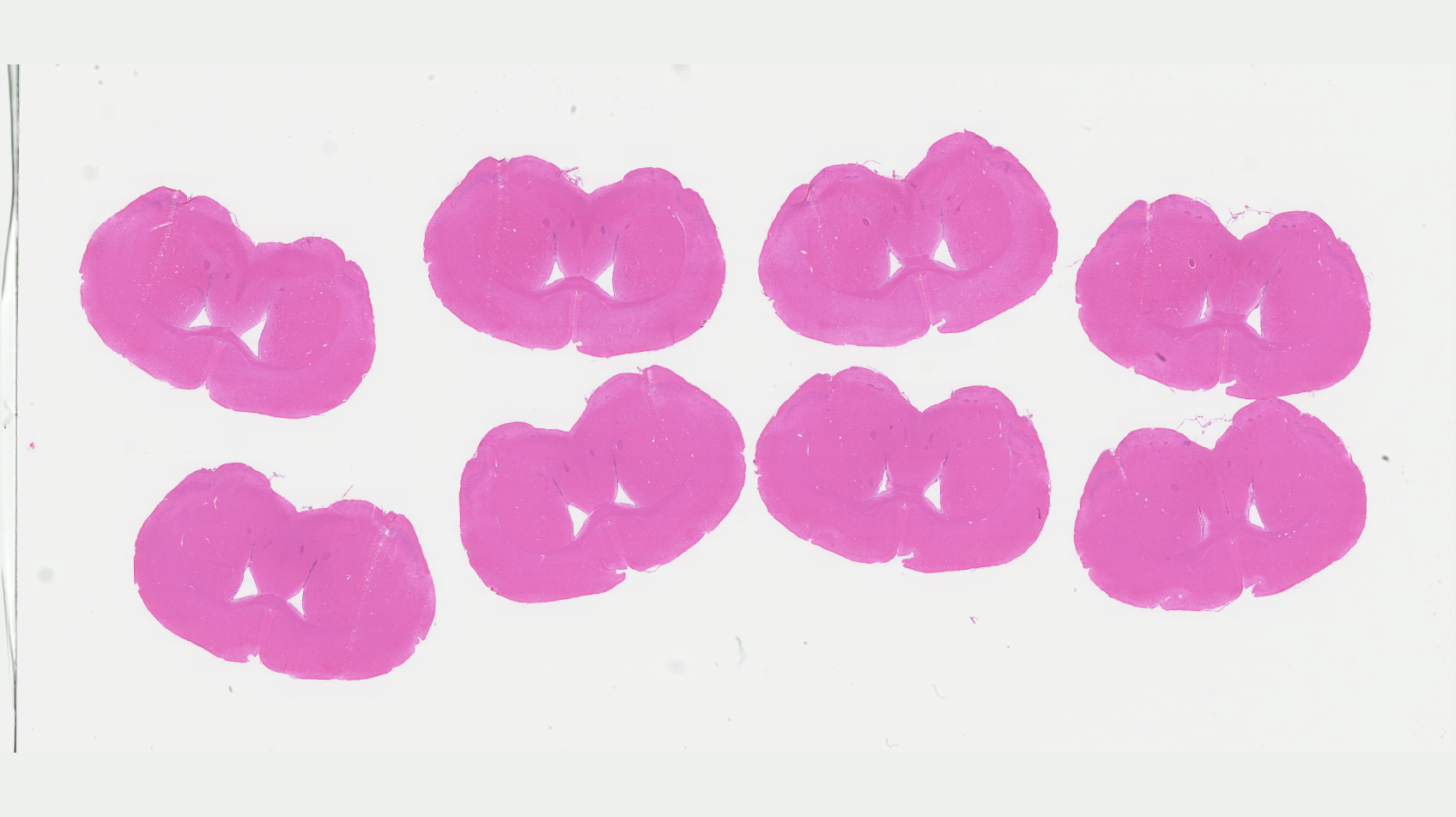

Supplement: Supplementary file 7 — Source data Fig. 5 [file 44321_2024_148_MOESM7_ESM.zip › Figure 5/A-B/Figure A/corpus callosum/CBH3 - 323266.tif]

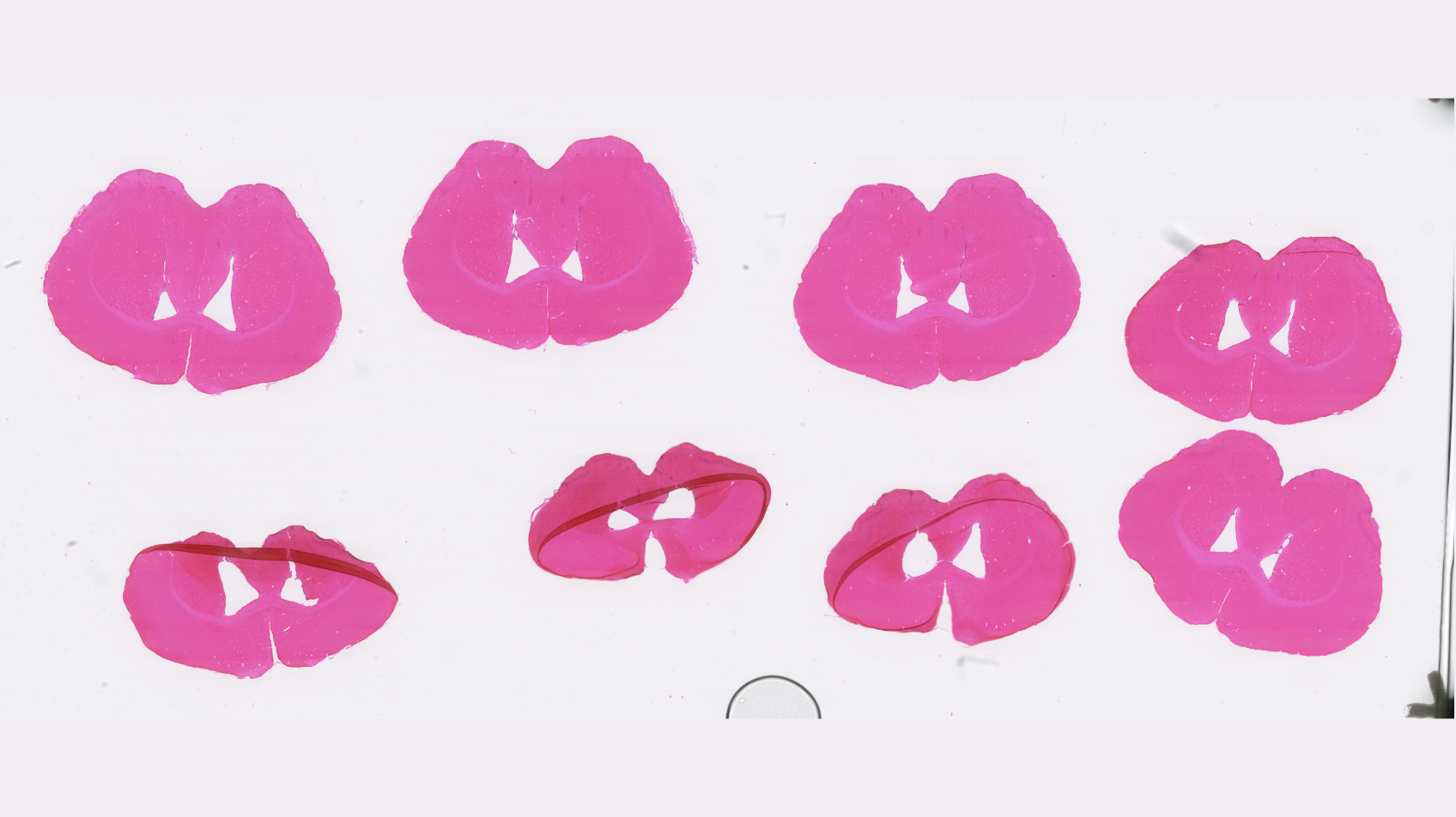

Supplement: Supplementary file 7 — Source data Fig. 5 [file 44321_2024_148_MOESM7_ESM.zip › Figure 5/A-B/Figure A/corpus callosum/cbh4 - 325298.tif]

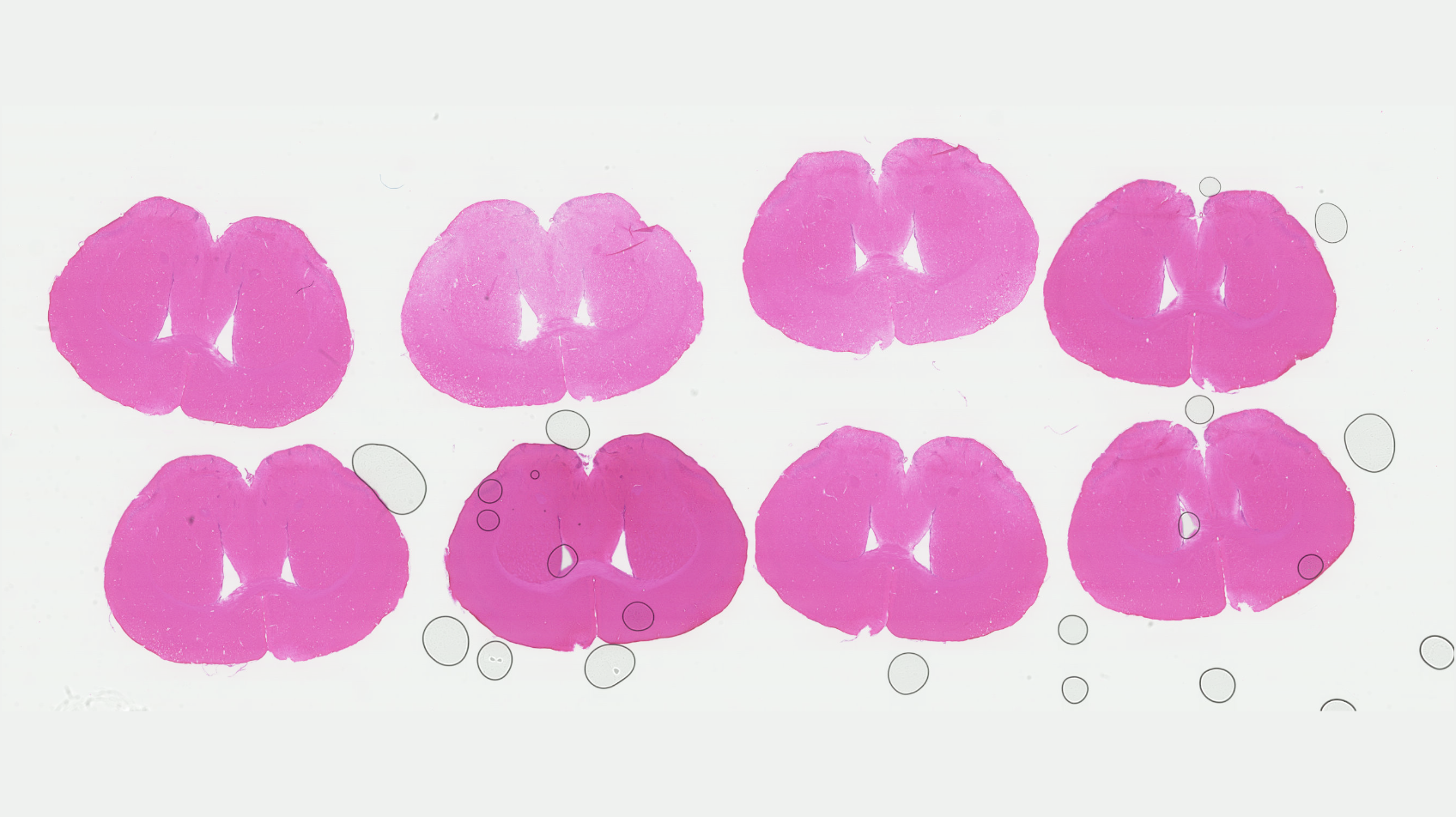

Supplement: Supplementary file 7 — Source data Fig. 5 [file 44321_2024_148_MOESM7_ESM.zip › Figure 5/A-B/Figure A/corpus callosum/SYN 2 - 325076.tif]

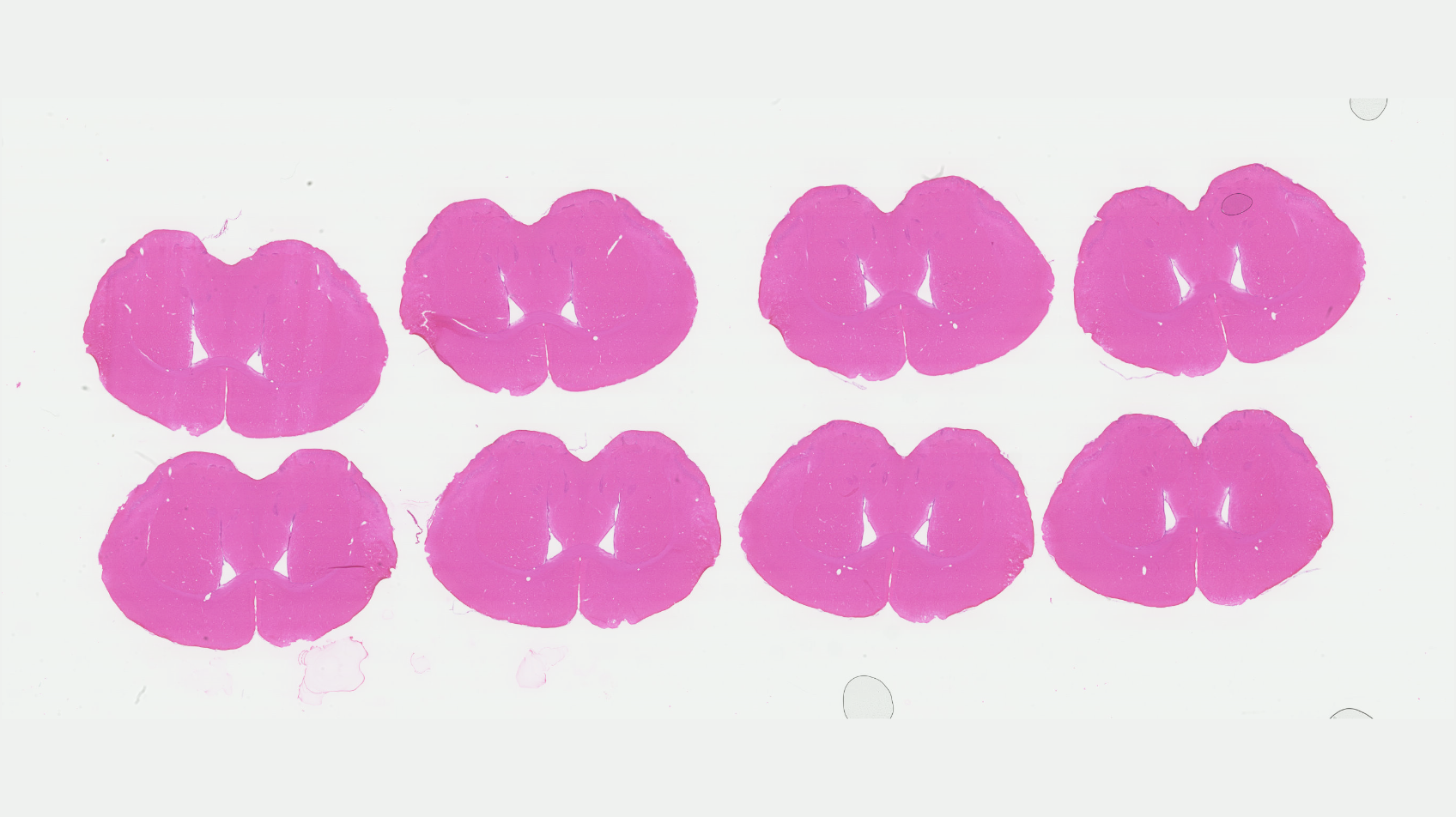

Supplement: Supplementary file 7 — Source data Fig. 5 [file 44321_2024_148_MOESM7_ESM.zip › Figure 5/A-B/Figure A/corpus callosum/SYN N3 - 325307.tif]

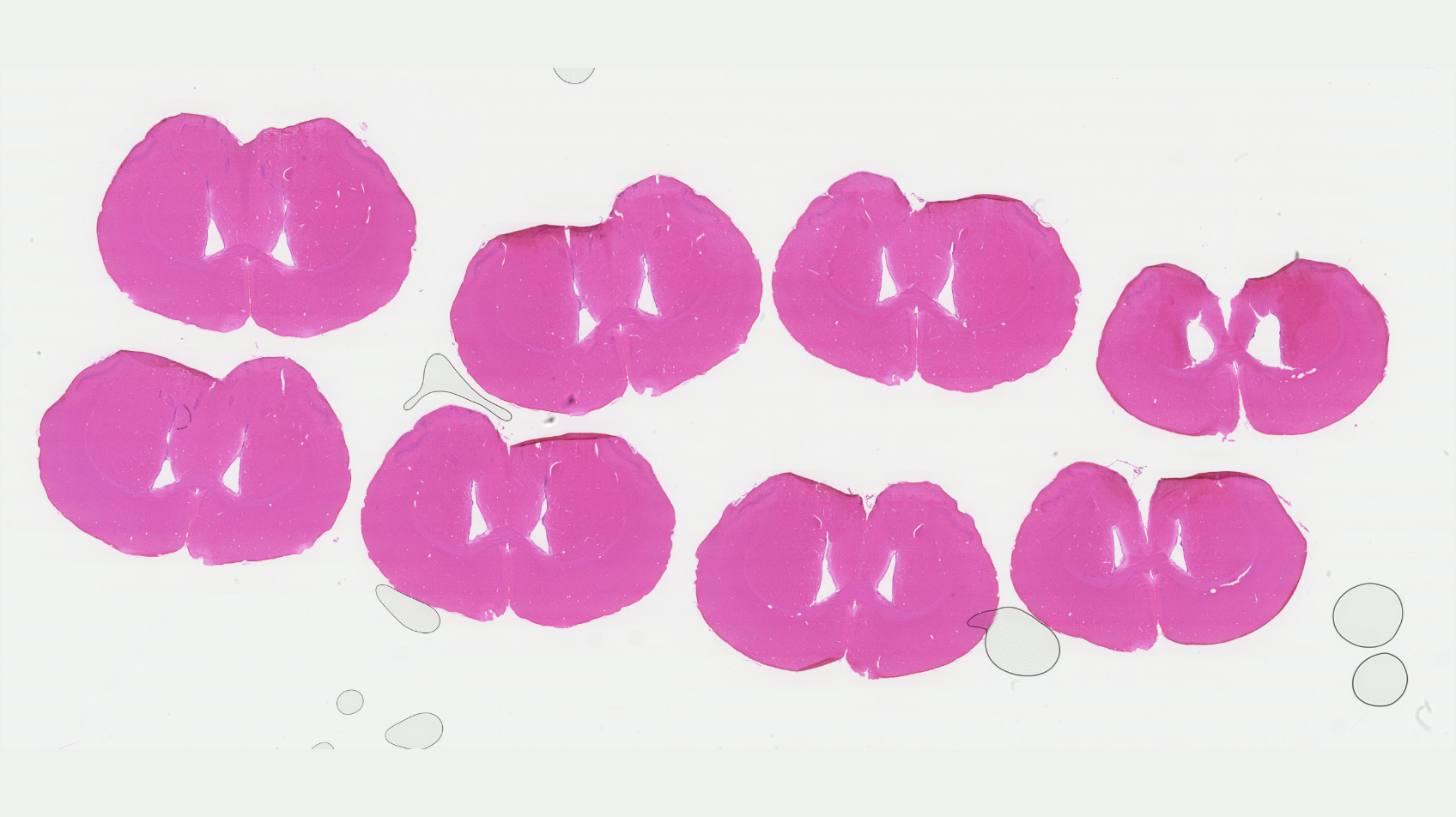

Supplement: Supplementary file 7 — Source data Fig. 5 [file 44321_2024_148_MOESM7_ESM.zip › Figure 5/A-B/Figure A/corpus callosum/SYN N4 - 325295.tif]

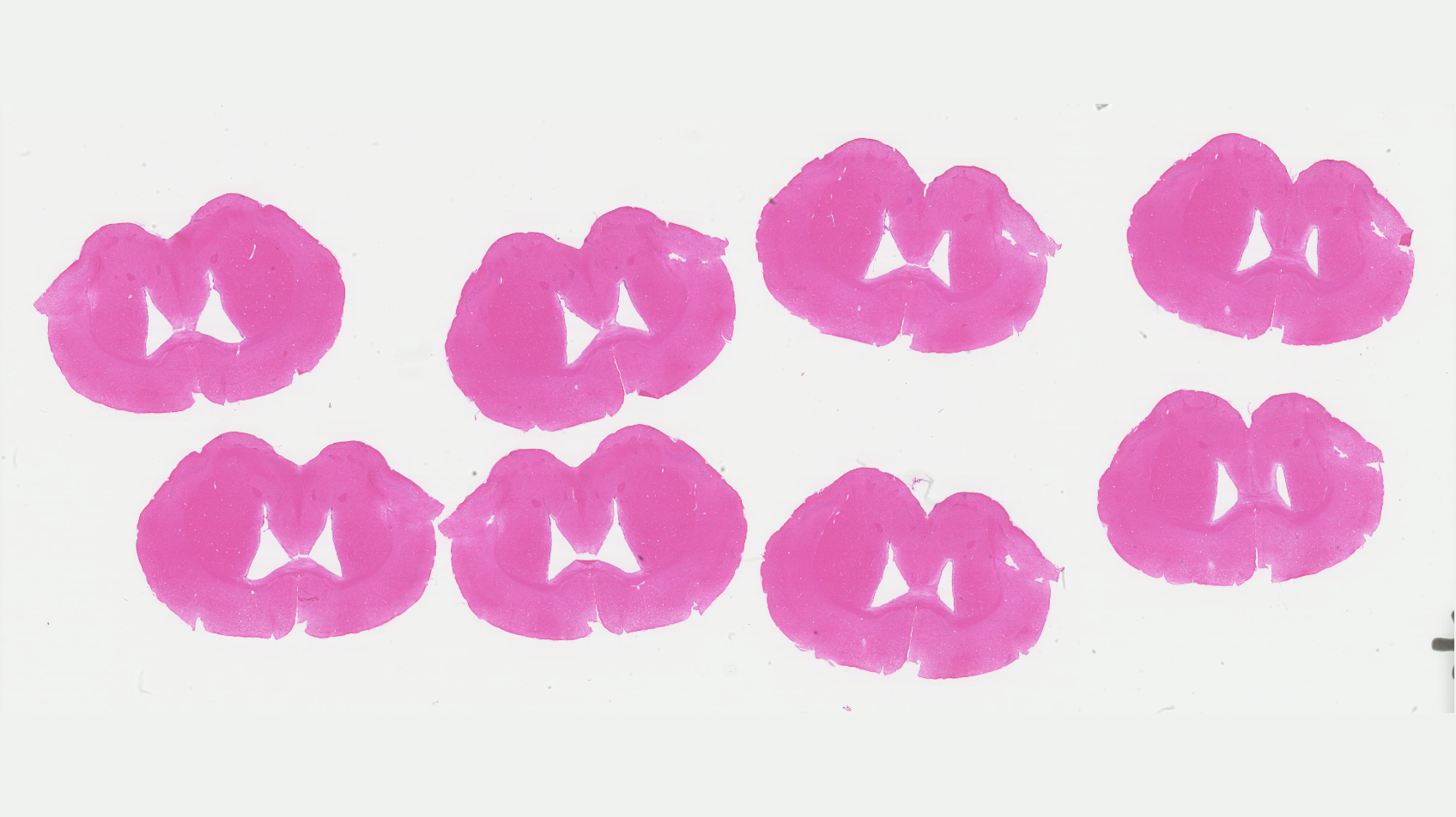

Supplement: Supplementary file 7 — Source data Fig. 5 [file 44321_2024_148_MOESM7_ESM.zip › Figure 5/A-B/Figure A/corpus callosum/V5.1 - 323259.tif]

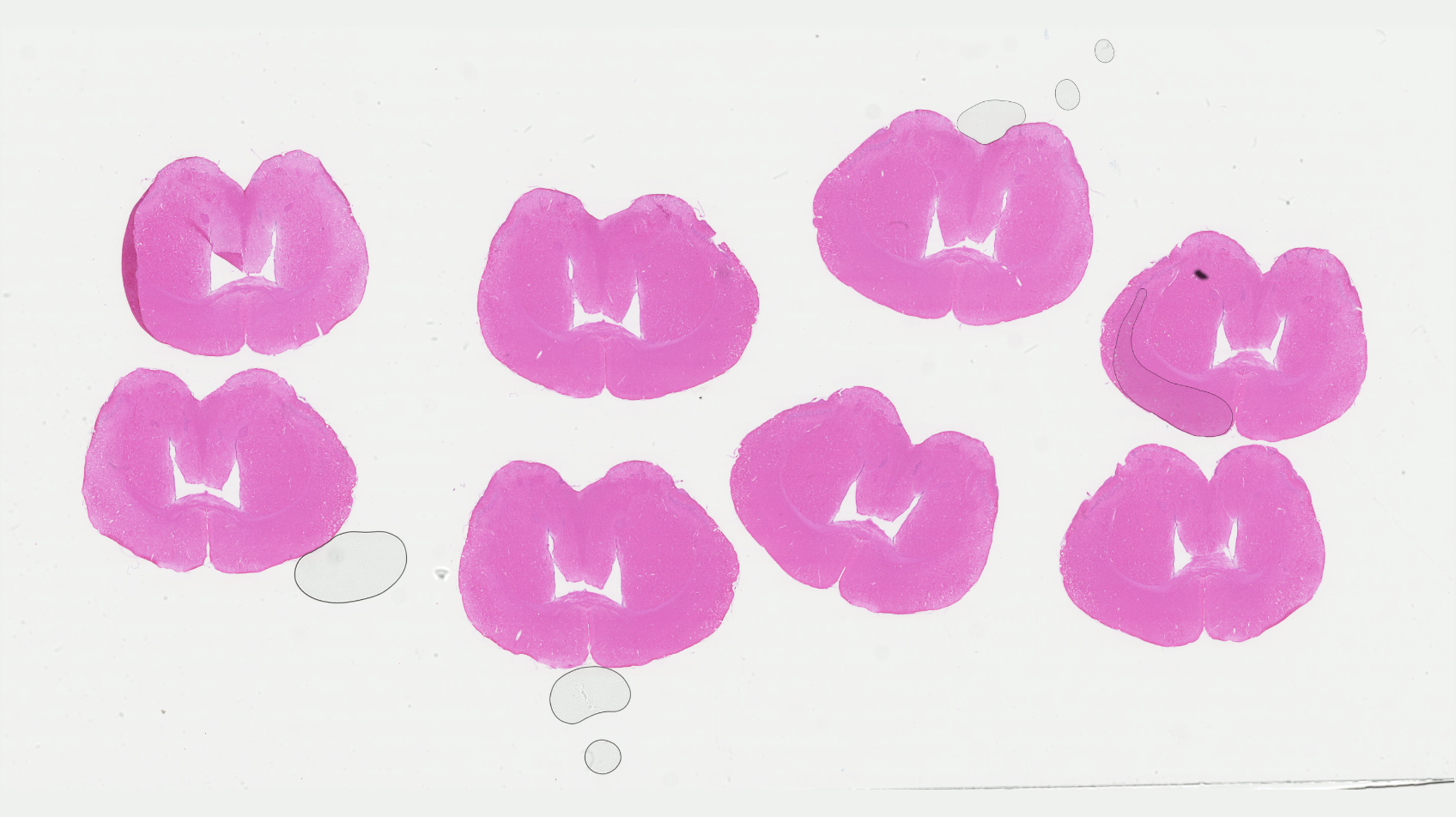

Supplement: Supplementary file 7 — Source data Fig. 5 [file 44321_2024_148_MOESM7_ESM.zip › Figure 5/A-B/Figure A/corpus callosum/V5.4 - 325308.tif]

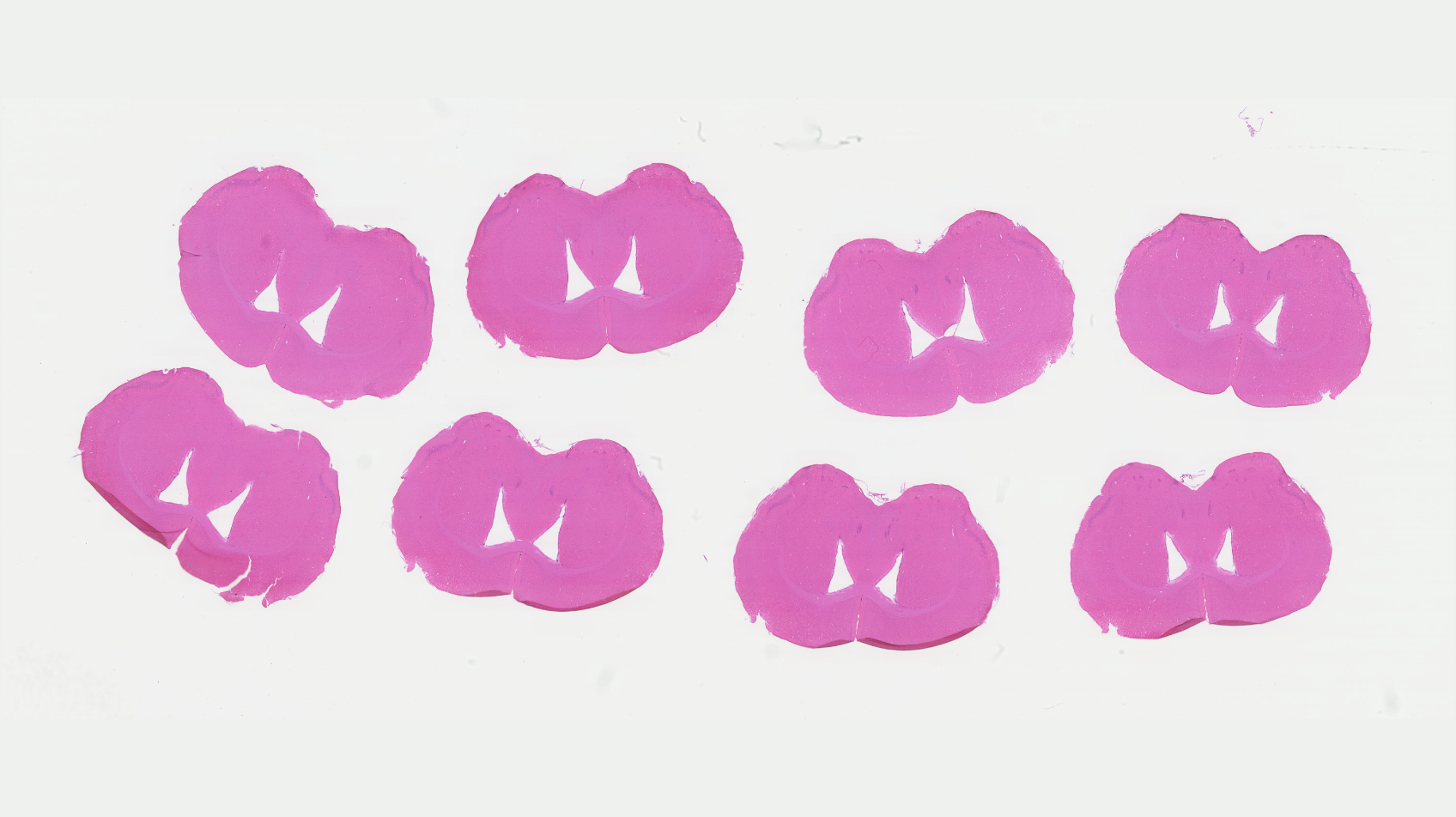

Supplement: Supplementary file 7 — Source data Fig. 5 [file 44321_2024_148_MOESM7_ESM.zip › Figure 5/A-B/Figure A/corpus callosum/V52 - 323263.tif]

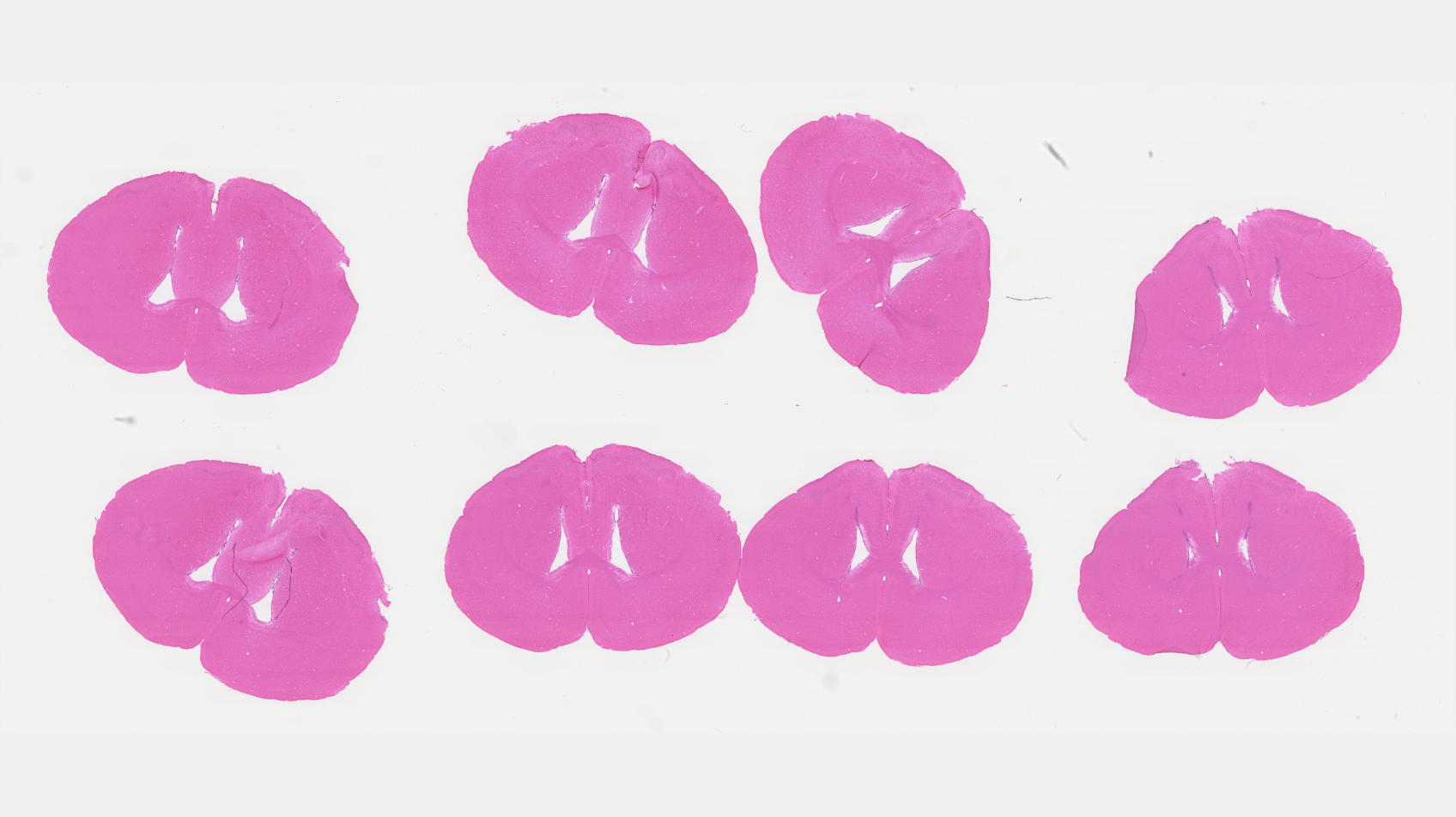

Supplement: Supplementary file 7 — Source data Fig. 5 [file 44321_2024_148_MOESM7_ESM.zip › Figure 5/A-B/Figure A/corpus callosum/V53 - 323264.tif]

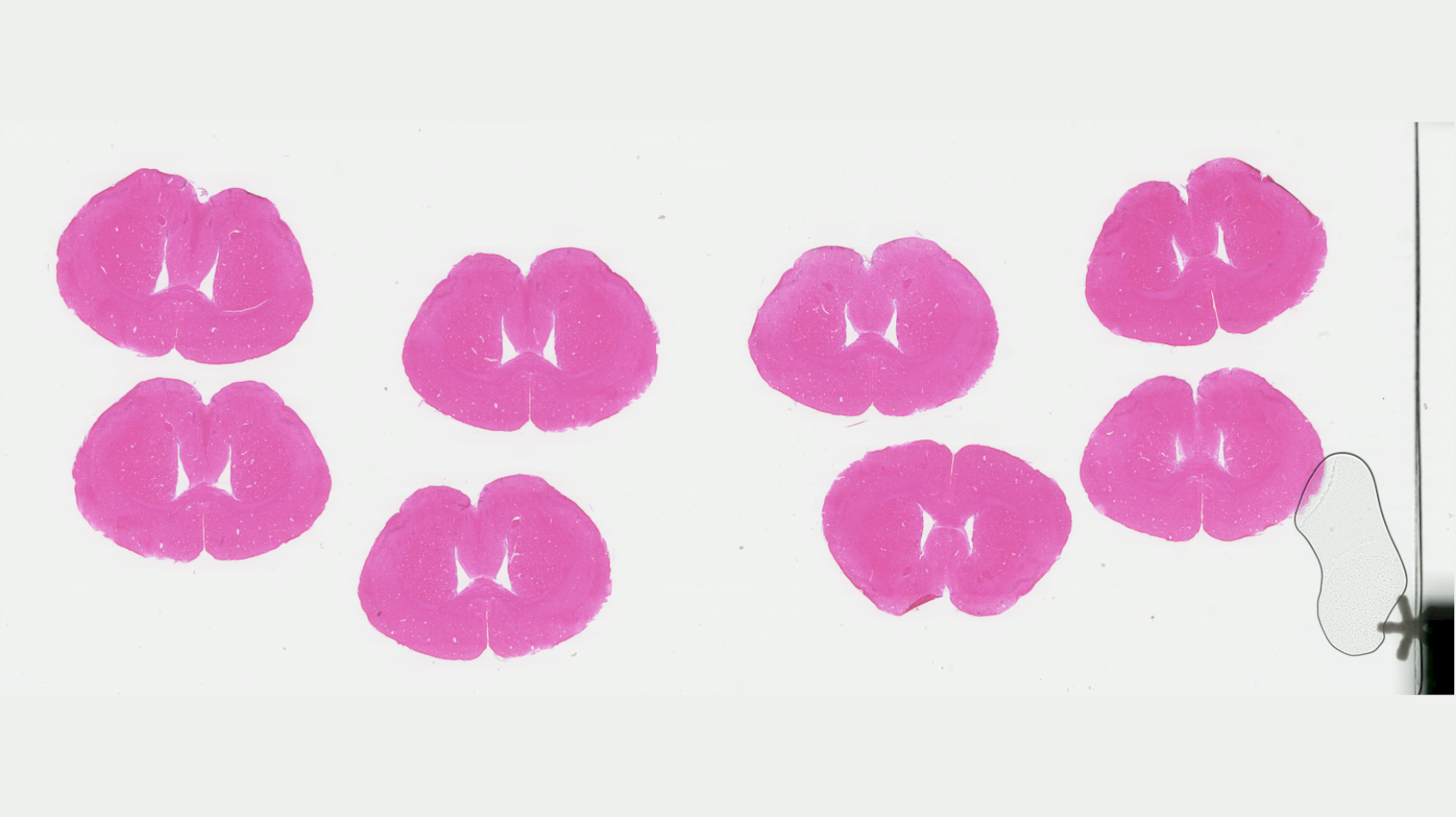

Supplement: Supplementary file 7 — Source data Fig. 5 [file 44321_2024_148_MOESM7_ESM.zip › Figure 5/A-B/Figure A/corpus callosum/WT 1 322019.tif]

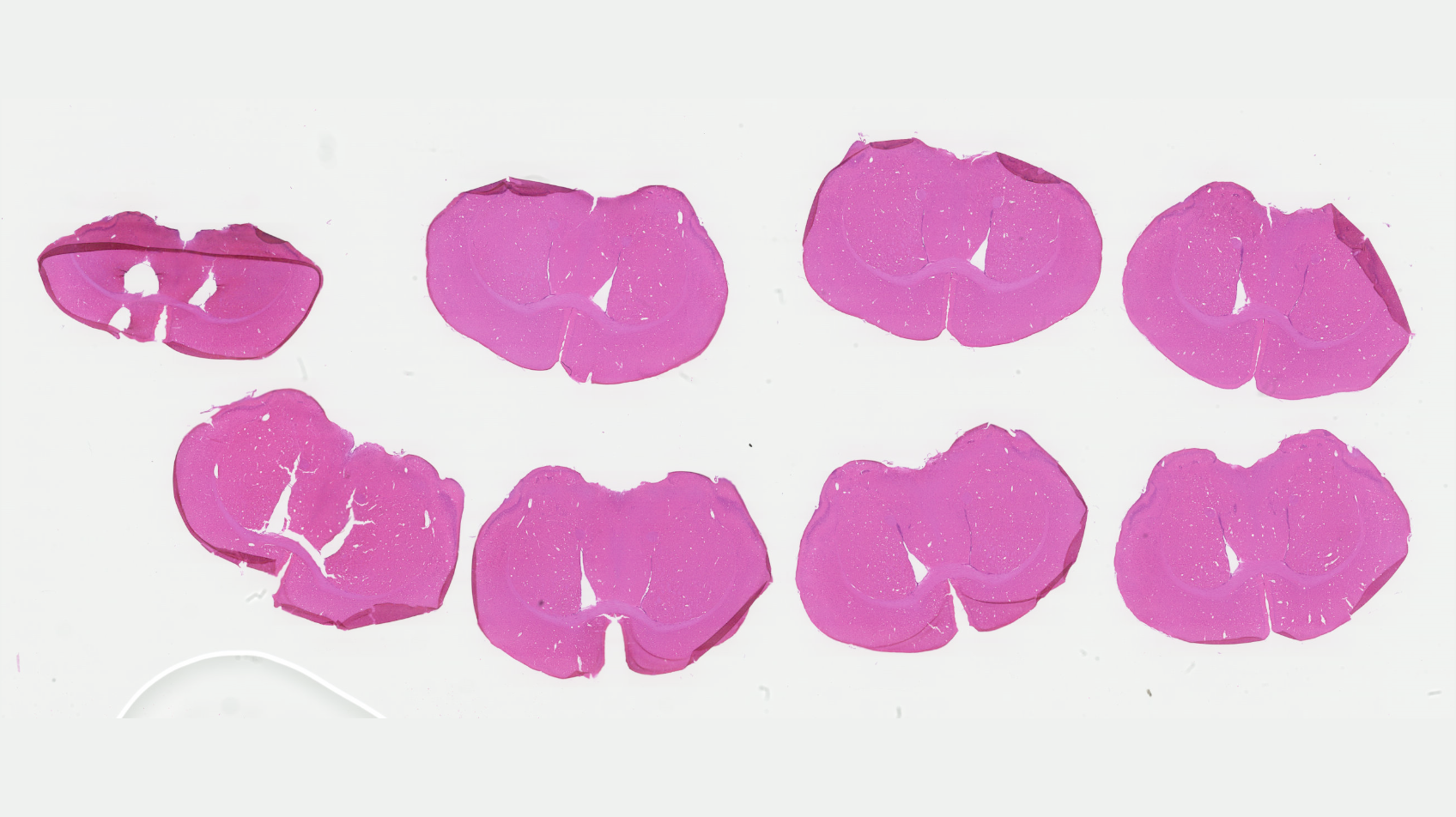

Supplement: Supplementary file 7 — Source data Fig. 5 [file 44321_2024_148_MOESM7_ESM.zip › Figure 5/A-B/Figure A/corpus callosum/WT2 - 322496.tif]

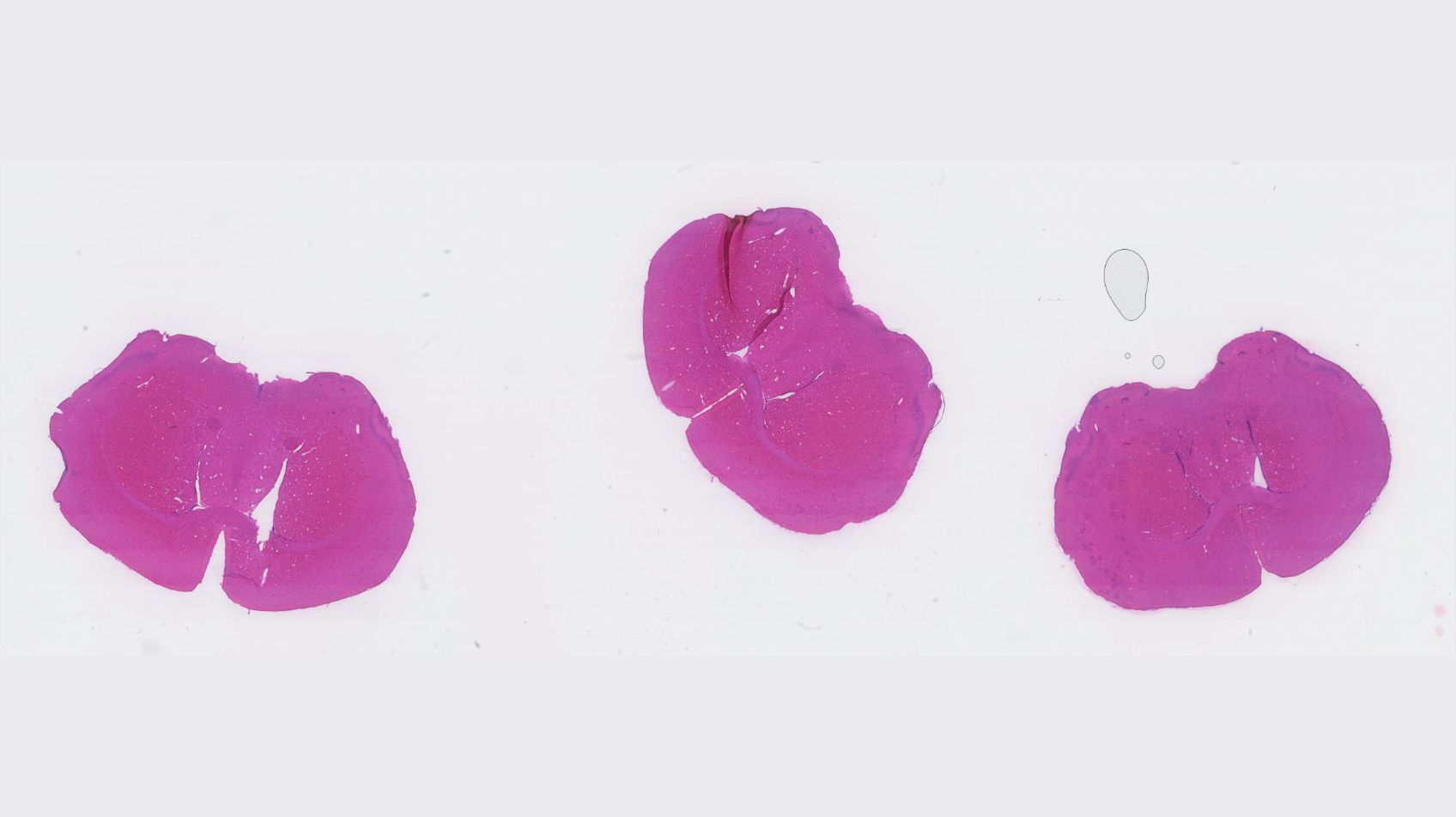

Supplement: Supplementary file 7 — Source data Fig. 5 [file 44321_2024_148_MOESM7_ESM.zip › Figure 5/A-B/Figure A/corpus callosum/WT2 section 8 and 9 replacments.tif]

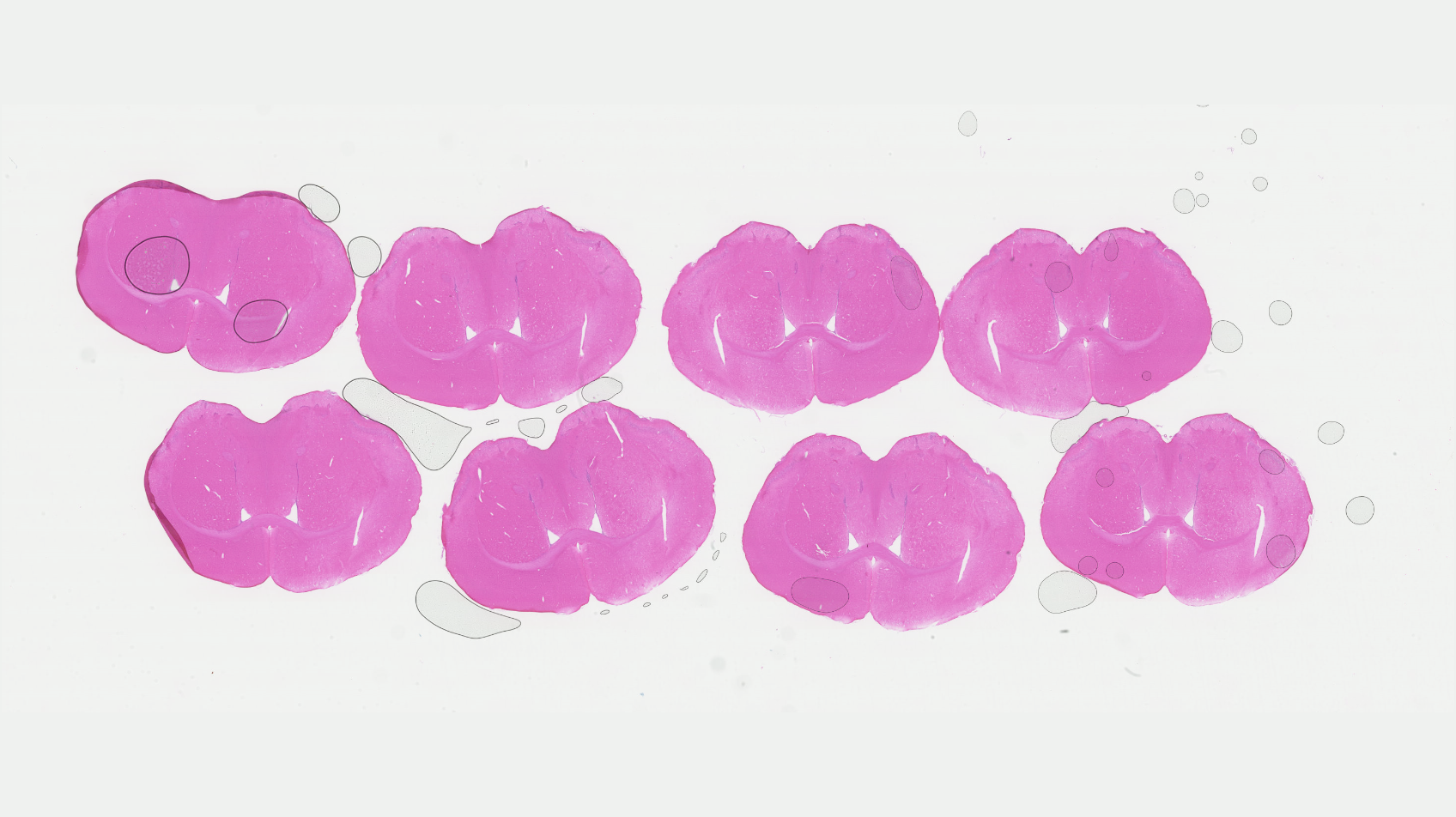

Supplement: Supplementary file 7 — Source data Fig. 5 [file 44321_2024_148_MOESM7_ESM.zip › Figure 5/A-B/Figure A/corpus callosum/WT4 - 325538.tif]

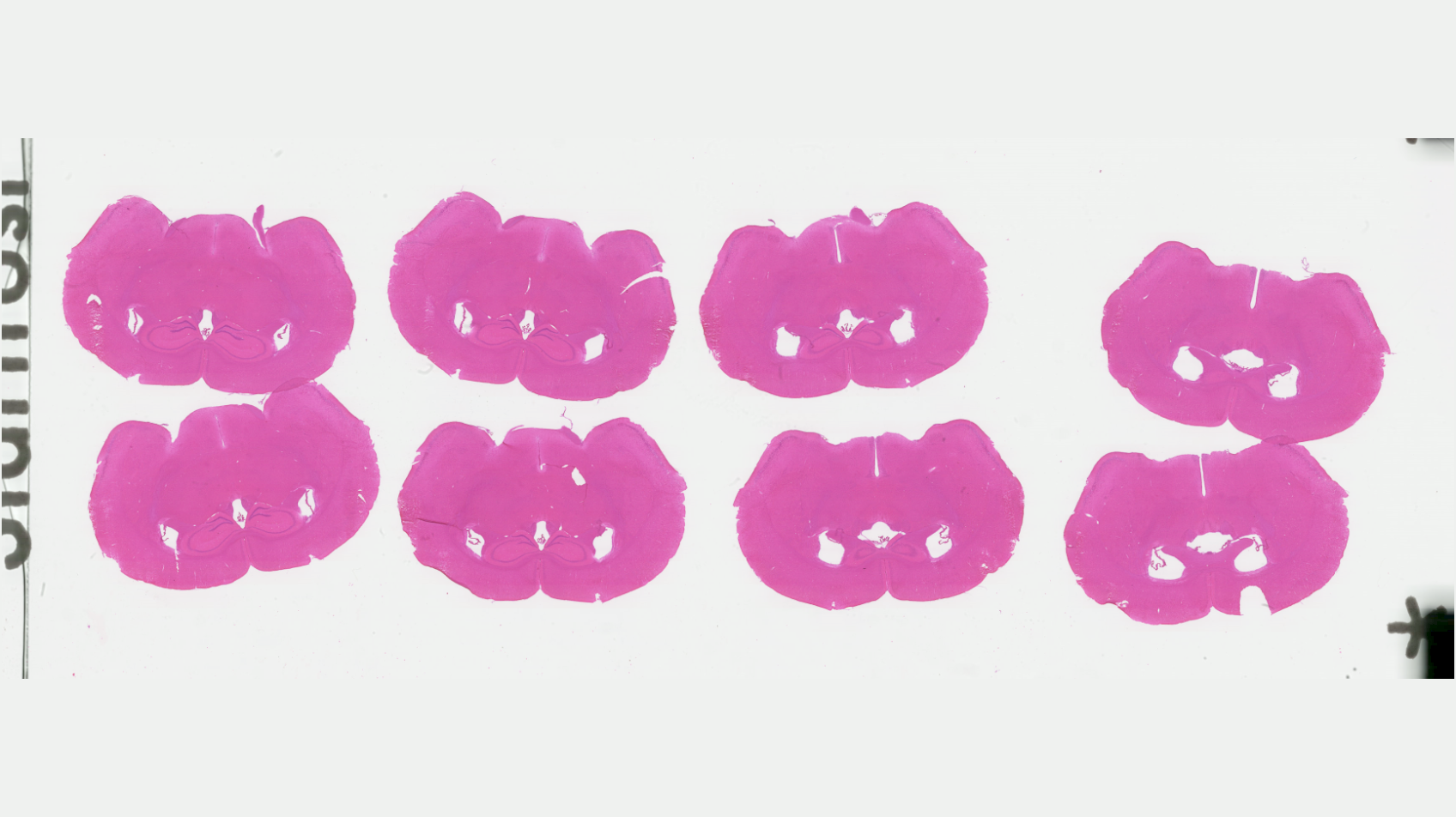

Supplement: Supplementary file 7 — Source data Fig. 5 [file 44321_2024_148_MOESM7_ESM.zip › Figure 5/A-B/Figure B/lateral ventricle/CBH 1 - 324362.tif]

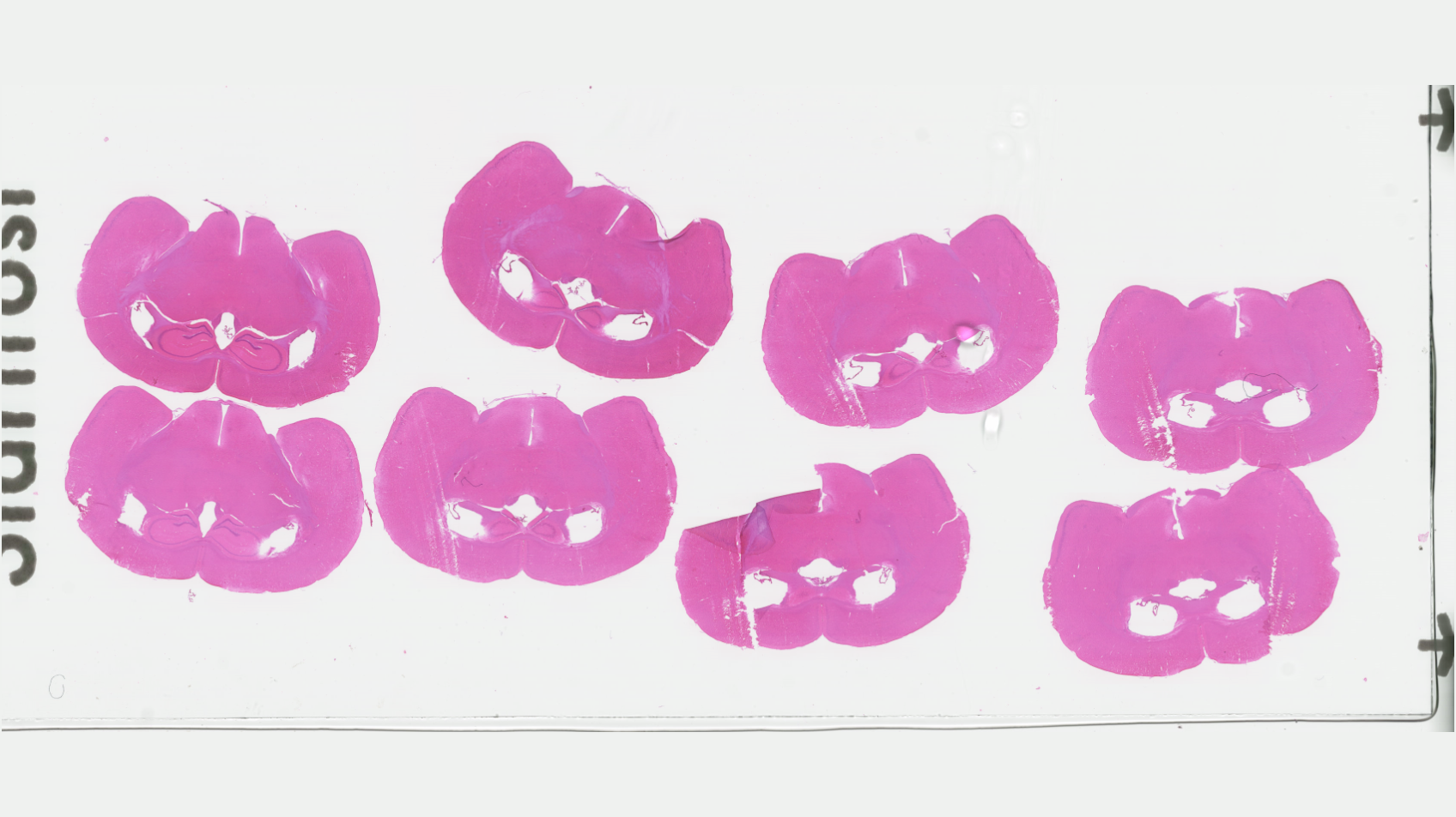

Supplement: Supplementary file 7 — Source data Fig. 5 [file 44321_2024_148_MOESM7_ESM.zip › Figure 5/A-B/Figure B/lateral ventricle/CBH2 - 324360.tif]

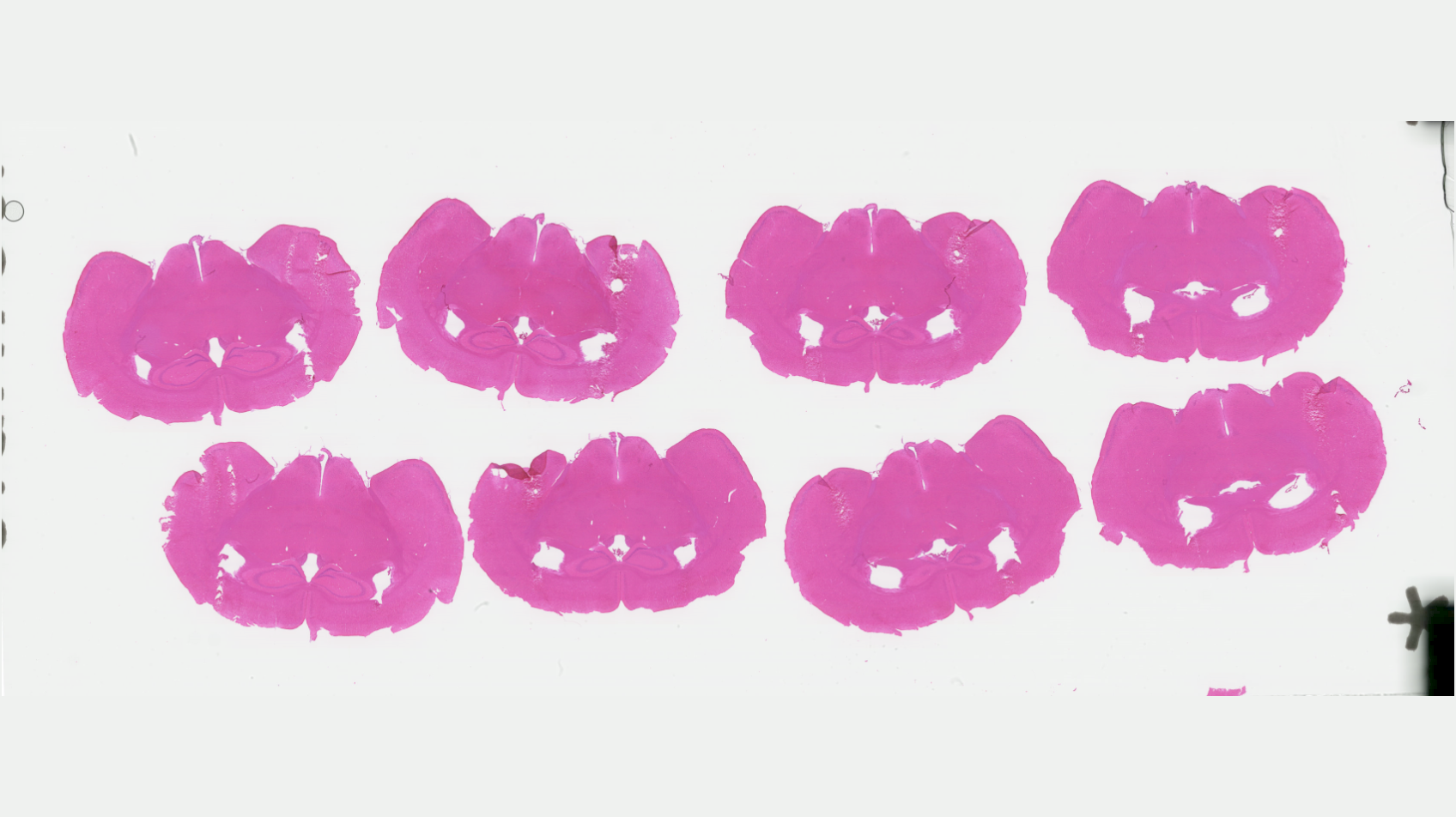

Supplement: Supplementary file 7 — Source data Fig. 5 [file 44321_2024_148_MOESM7_ESM.zip › Figure 5/A-B/Figure B/lateral ventricle/CBH3 323266.tif]

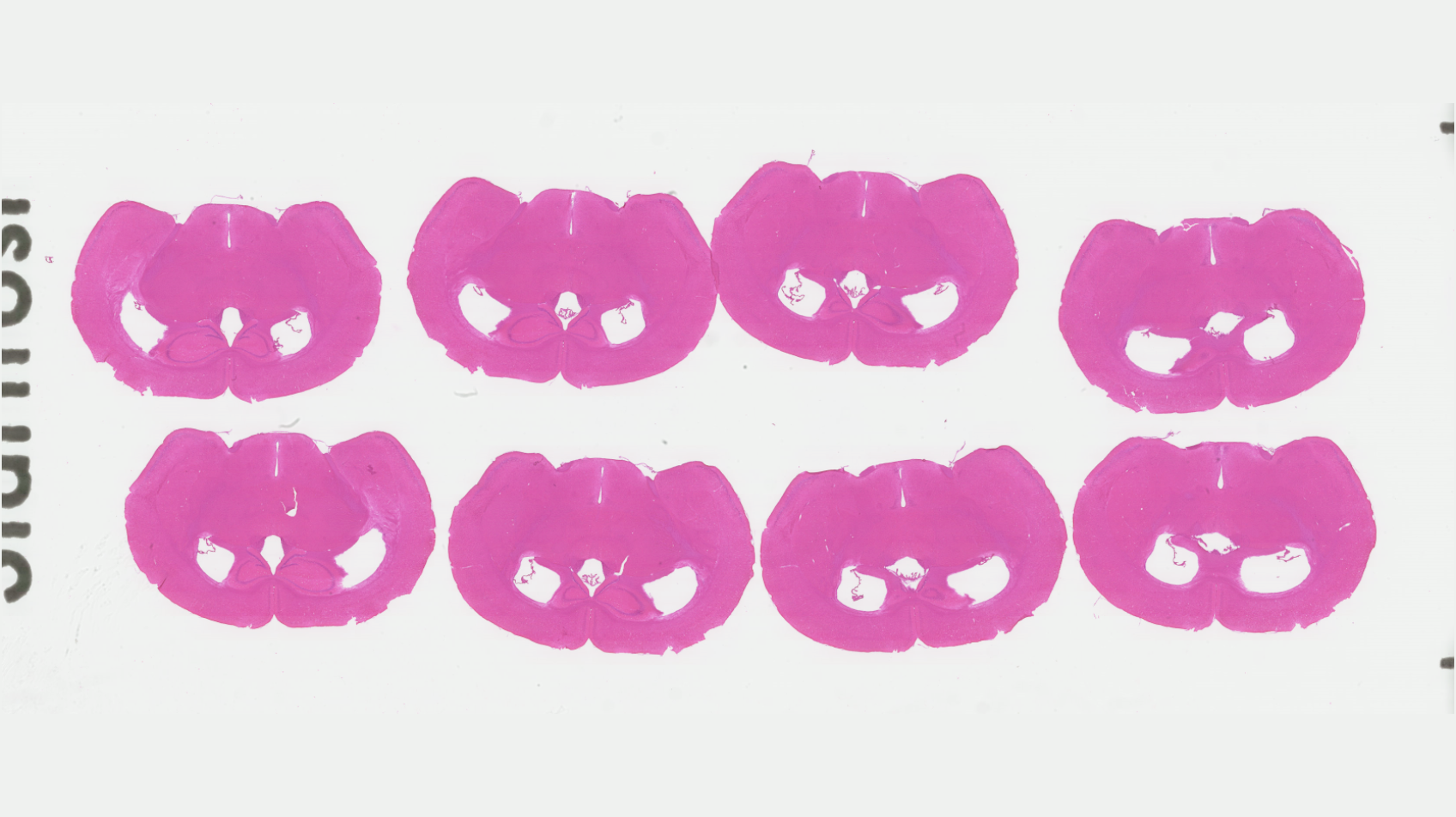

Supplement: Supplementary file 7 — Source data Fig. 5 [file 44321_2024_148_MOESM7_ESM.zip › Figure 5/A-B/Figure B/lateral ventricle/V5 1 - 323259.tif]

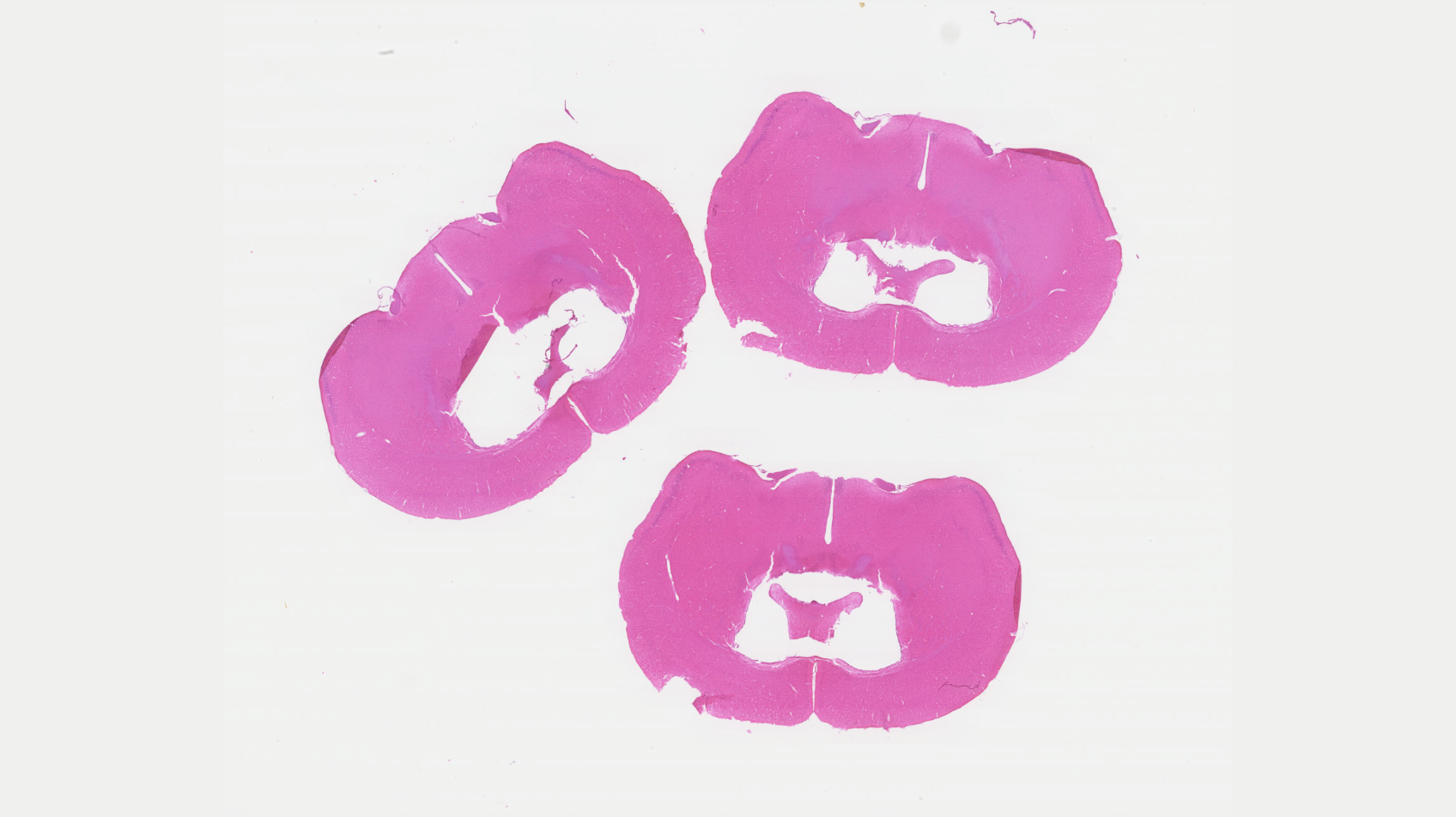

Supplement: Supplementary file 7 — Source data Fig. 5 [file 44321_2024_148_MOESM7_ESM.zip › Figure 5/A-B/Figure B/lateral ventricle/V5.2 323263.tif]

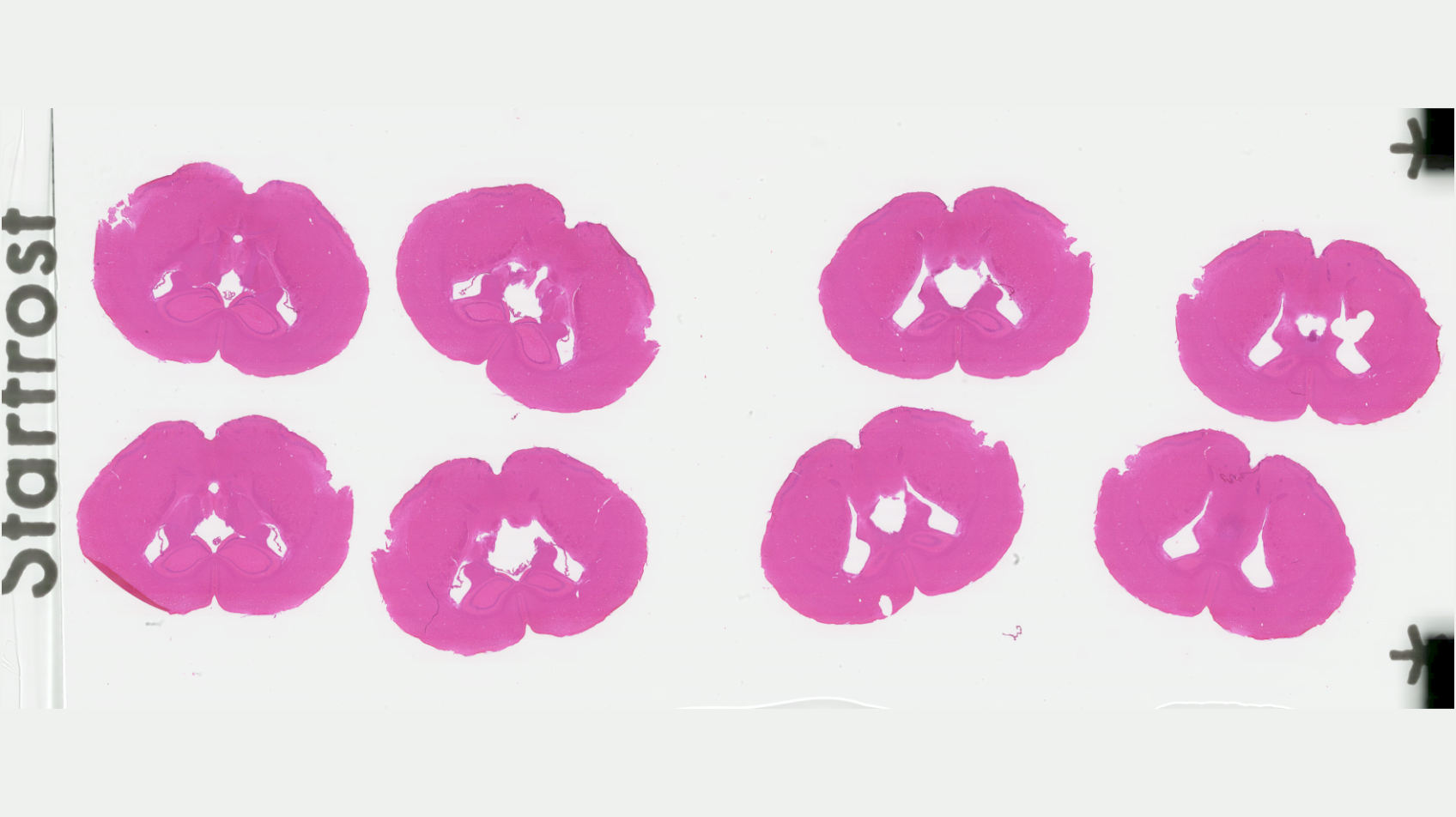

Supplement: Supplementary file 7 — Source data Fig. 5 [file 44321_2024_148_MOESM7_ESM.zip › Figure 5/A-B/Figure B/lateral ventricle/V5.3 323264.tif]

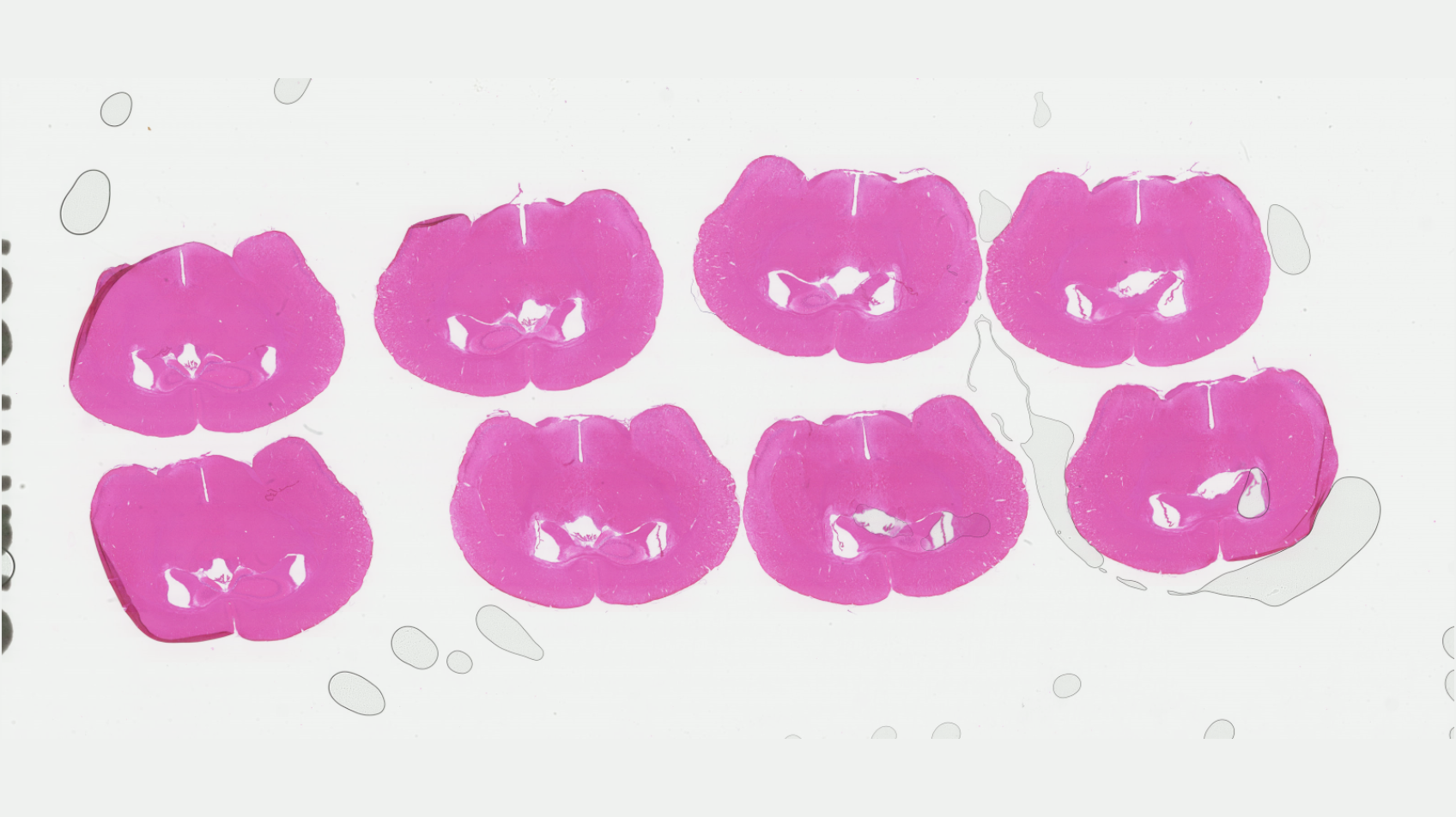

Supplement: Supplementary file 7 — Source data Fig. 5 [file 44321_2024_148_MOESM7_ESM.zip › Figure 5/A-B/Figure B/lateral ventricle/V5.4 325308.tif]

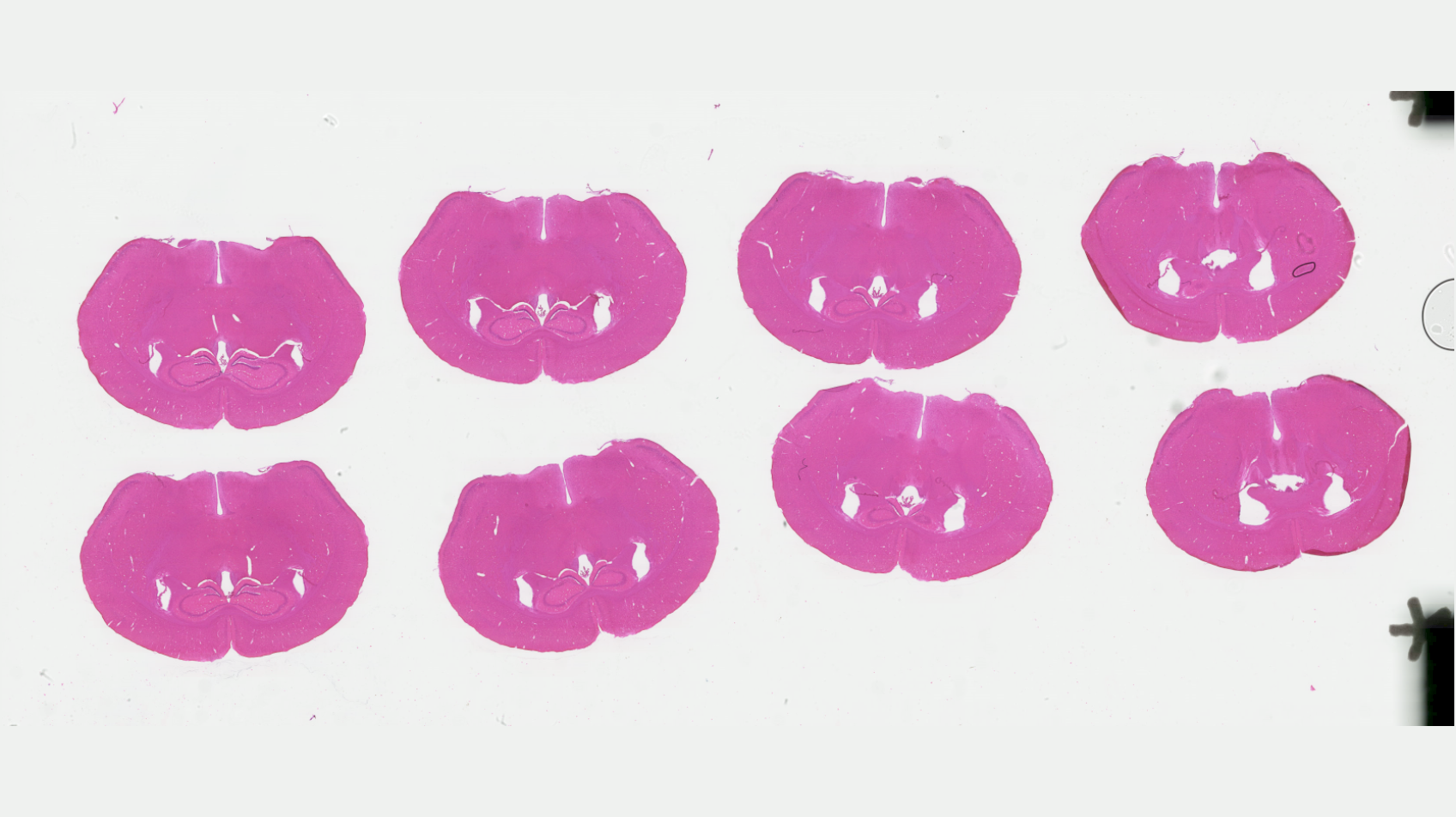

Supplement: Supplementary file 7 — Source data Fig. 5 [file 44321_2024_148_MOESM7_ESM.zip › Figure 5/A-B/Figure B/lateral ventricle/WT 1 - 322019.tif]

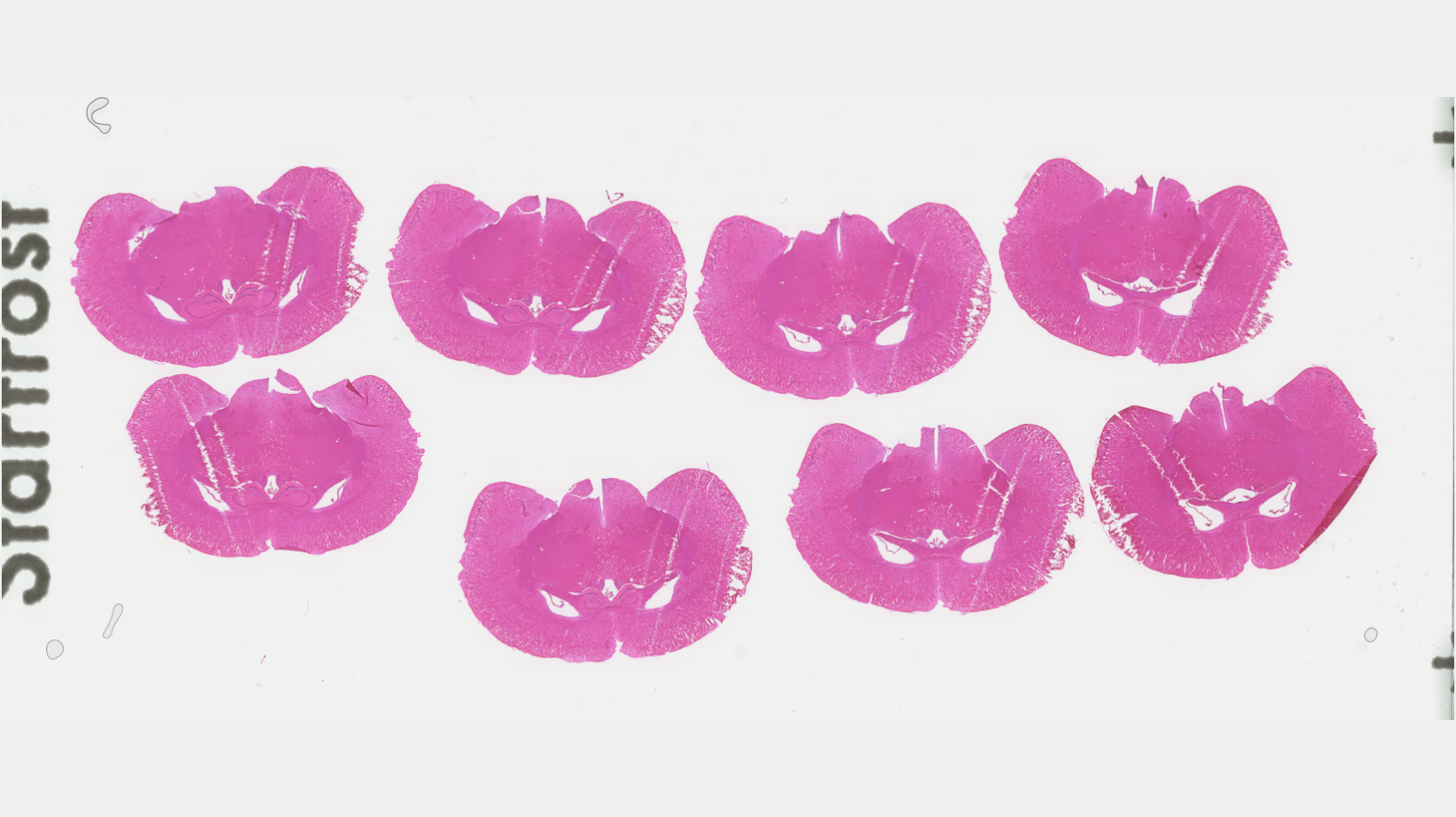

Supplement: Supplementary file 7 — Source data Fig. 5 [file 44321_2024_148_MOESM7_ESM.zip › Figure 5/A-B/Figure B/lateral ventricle/WT3 323394.tif]

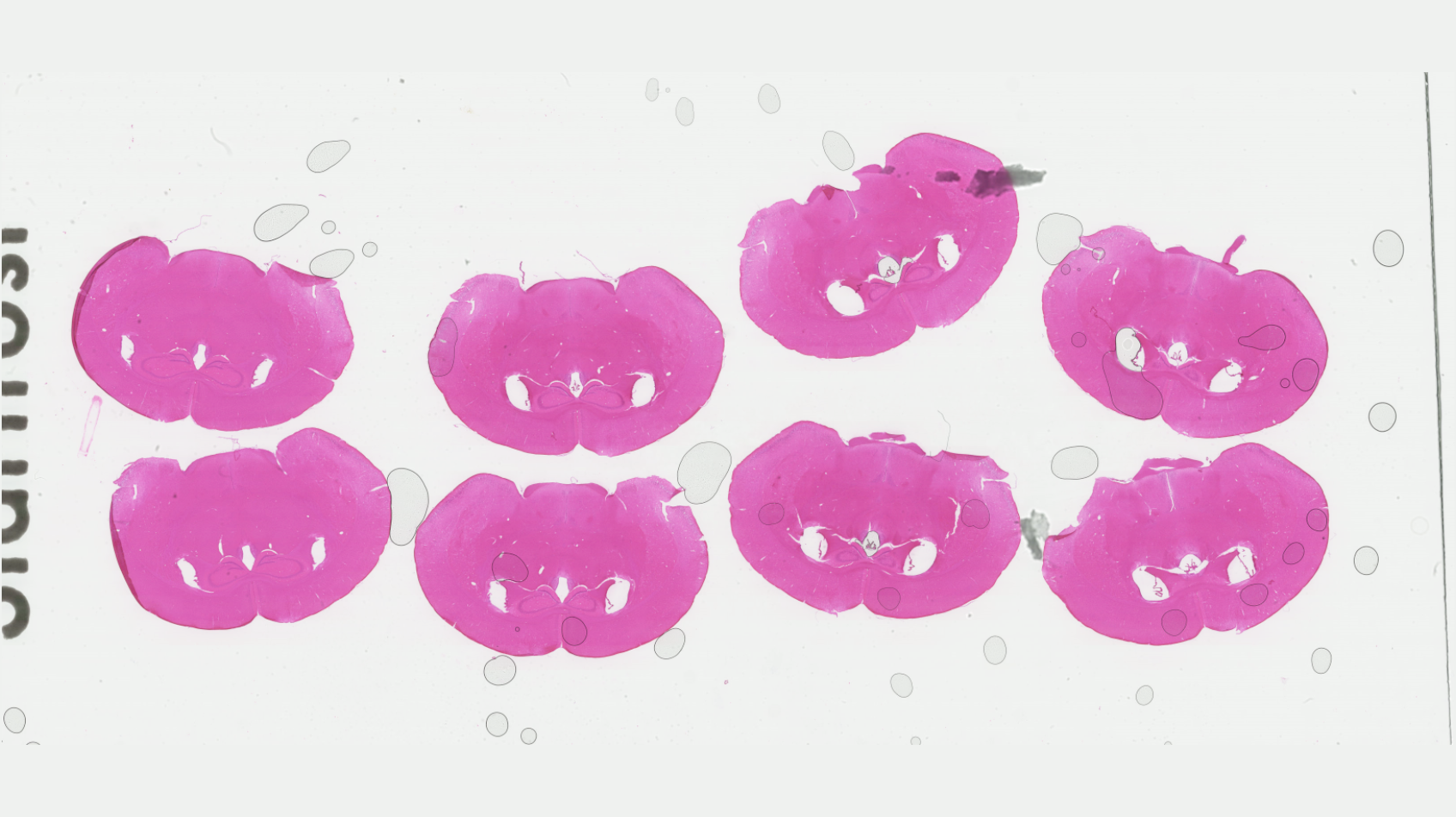

Supplement: Supplementary file 7 — Source data Fig. 5 [file 44321_2024_148_MOESM7_ESM.zip › Figure 5/A-B/Figure B/lateral ventricle/WT4 325538.tif]

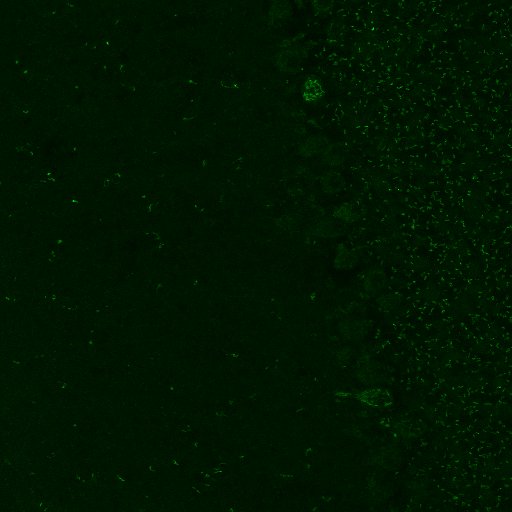

Supplement: Supplementary file 7 — Source data Fig. 5 [file 44321_2024_148_MOESM7_ESM.zip › Figure 5/C/Cbh 4 9 months.lif - cereb 3_ZProject.jpg]

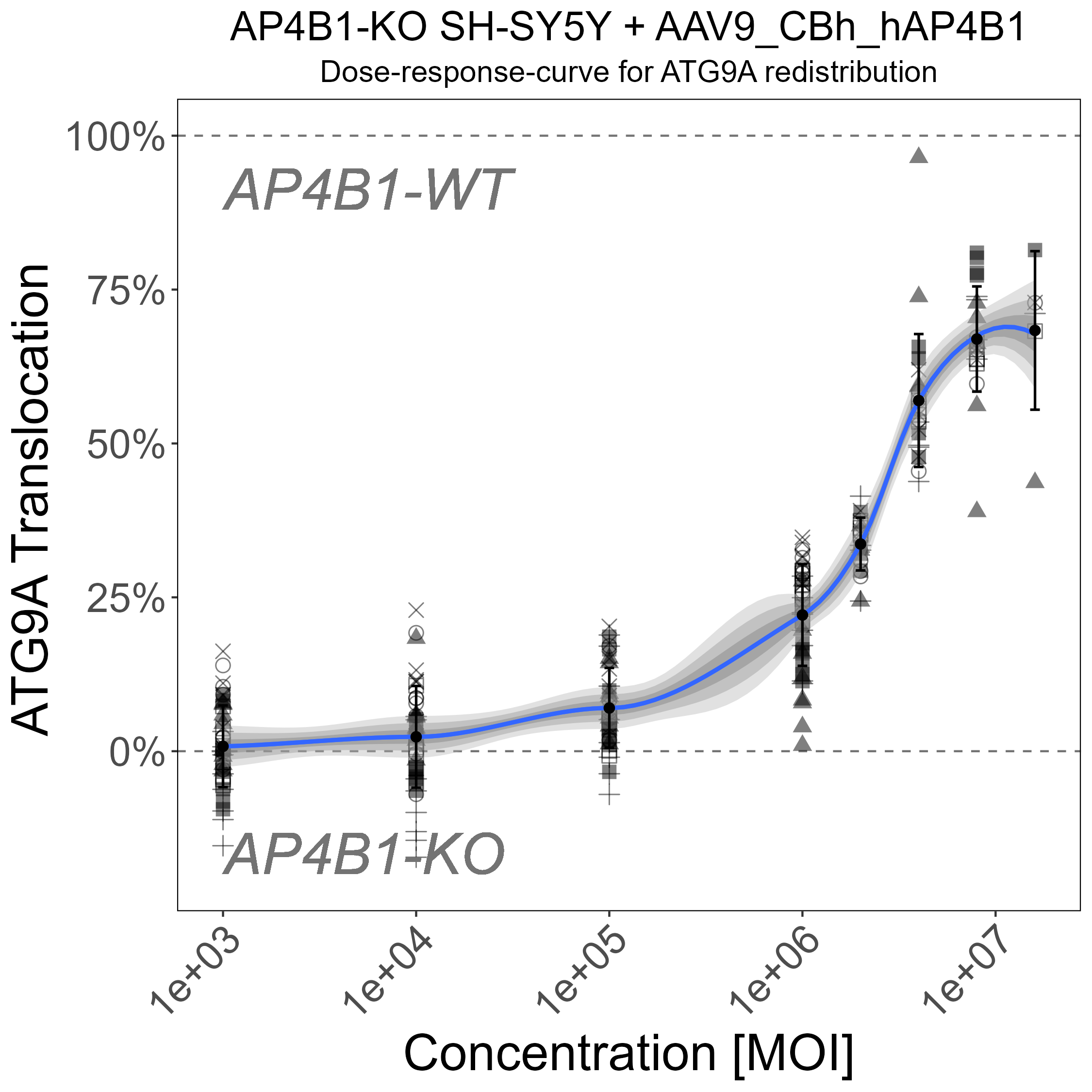

Supplement: Supplementary file 9 — Source data Fig. 7 [file 44321_2024_148_MOESM9_ESM.zip › Figure 7/A-B and D/ATG9A translocation.png]

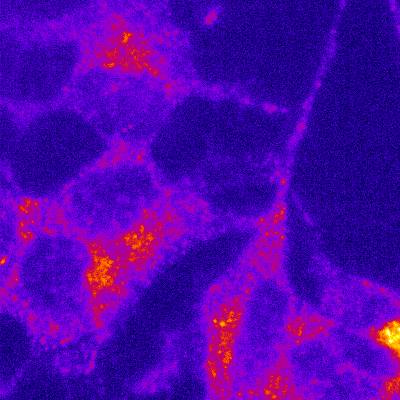

Supplement: Supplementary file 9 — Source data Fig. 7 [file 44321_2024_148_MOESM9_ESM.zip › Figure 7/C/16E7 ATG9A fire.png]

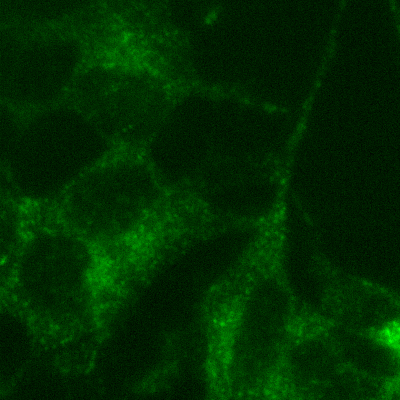

Supplement: Supplementary file 9 — Source data Fig. 7 [file 44321_2024_148_MOESM9_ESM.zip › Figure 7/C/16E7 ATG9A.png]

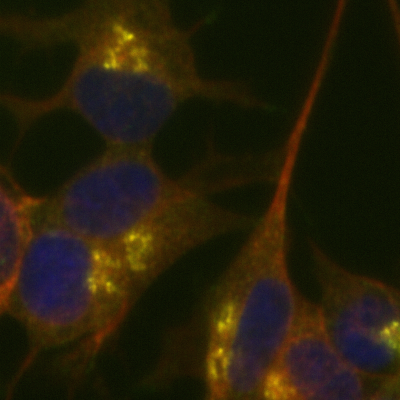

Supplement: Supplementary file 9 — Source data Fig. 7 [file 44321_2024_148_MOESM9_ESM.zip › Figure 7/C/16E7 merge.png]

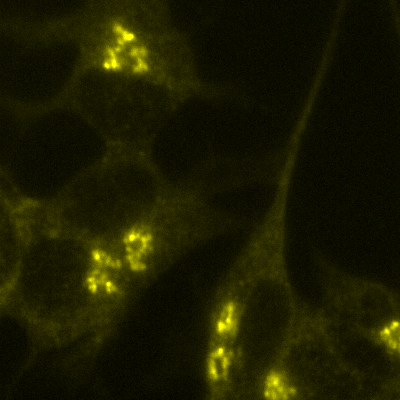

Supplement: Supplementary file 9 — Source data Fig. 7 [file 44321_2024_148_MOESM9_ESM.zip › Figure 7/C/16E7 TGN.png]

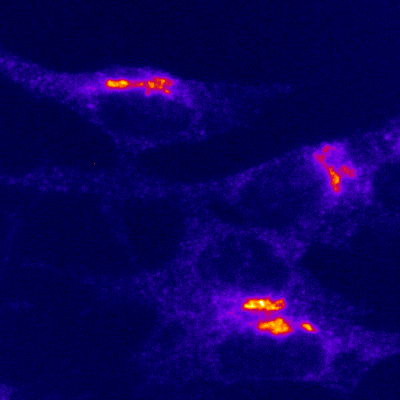

Supplement: Supplementary file 9 — Source data Fig. 7 [file 44321_2024_148_MOESM9_ESM.zip › Figure 7/C/1E4 ATG9A fire.png]

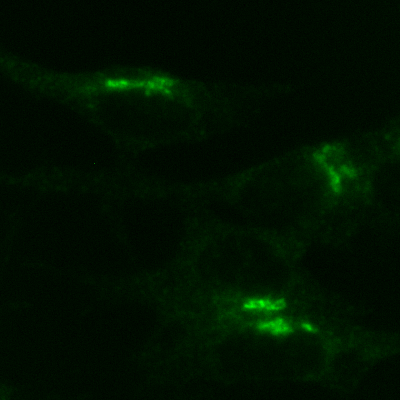

Supplement: Supplementary file 9 — Source data Fig. 7 [file 44321_2024_148_MOESM9_ESM.zip › Figure 7/C/1E4 ATG9A.png]

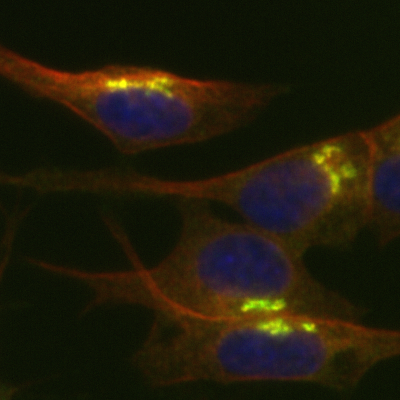

Supplement: Supplementary file 9 — Source data Fig. 7 [file 44321_2024_148_MOESM9_ESM.zip › Figure 7/C/1E4 merge.png]

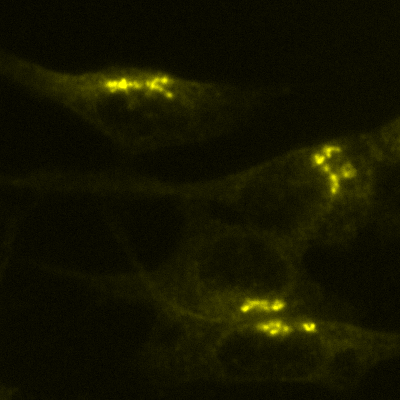

Supplement: Supplementary file 9 — Source data Fig. 7 [file 44321_2024_148_MOESM9_ESM.zip › Figure 7/C/1E4 TGN.png]

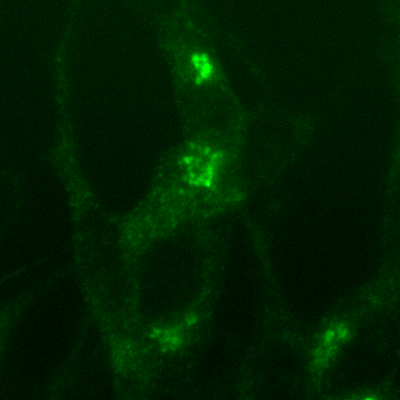

Supplement: Supplementary file 9 — Source data Fig. 7 [file 44321_2024_148_MOESM9_ESM.zip › Figure 7/C/2E6 ATG9A green.png]

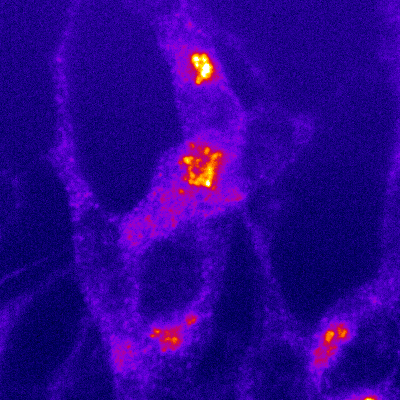

Supplement: Supplementary file 9 — Source data Fig. 7 [file 44321_2024_148_MOESM9_ESM.zip › Figure 7/C/2E6 ATG9A.png]

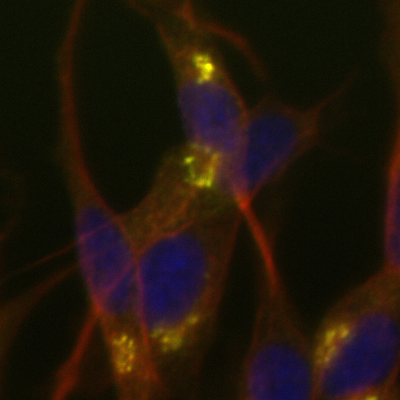

Supplement: Supplementary file 9 — Source data Fig. 7 [file 44321_2024_148_MOESM9_ESM.zip › Figure 7/C/2E6 merge.png]

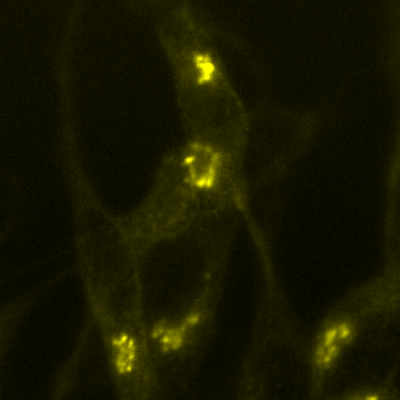

Supplement: Supplementary file 9 — Source data Fig. 7 [file 44321_2024_148_MOESM9_ESM.zip › Figure 7/C/2E6 TGN.png]

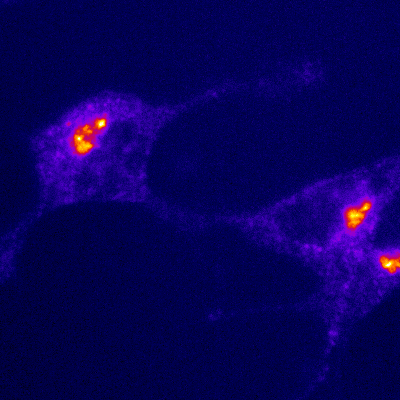

Supplement: Supplementary file 9 — Source data Fig. 7 [file 44321_2024_148_MOESM9_ESM.zip › Figure 7/C/KO ATG9A fire.png]

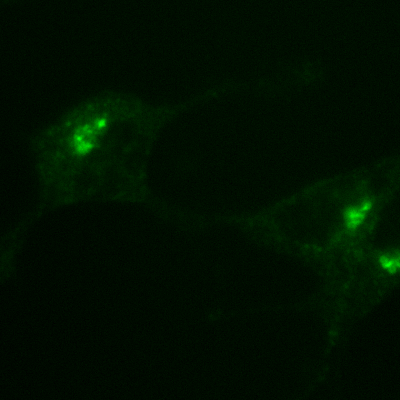

Supplement: Supplementary file 9 — Source data Fig. 7 [file 44321_2024_148_MOESM9_ESM.zip › Figure 7/C/Ko ATG9A.png]

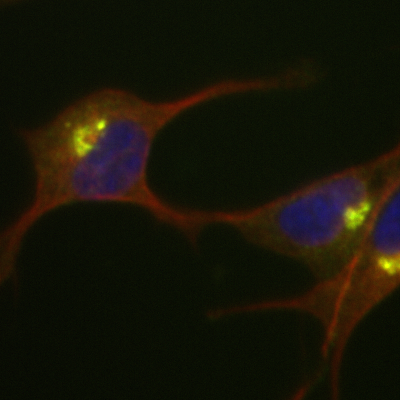

Supplement: Supplementary file 9 — Source data Fig. 7 [file 44321_2024_148_MOESM9_ESM.zip › Figure 7/C/KO merge.png]

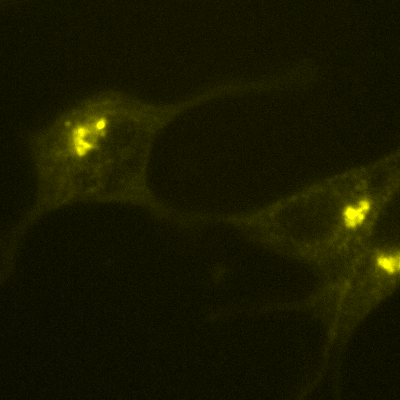

Supplement: Supplementary file 9 — Source data Fig. 7 [file 44321_2024_148_MOESM9_ESM.zip › Figure 7/C/KO TGN.png]

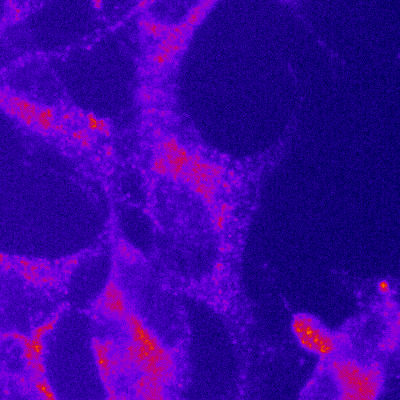

Supplement: Supplementary file 9 — Source data Fig. 7 [file 44321_2024_148_MOESM9_ESM.zip › Figure 7/C/WT ATG9A fire.png]

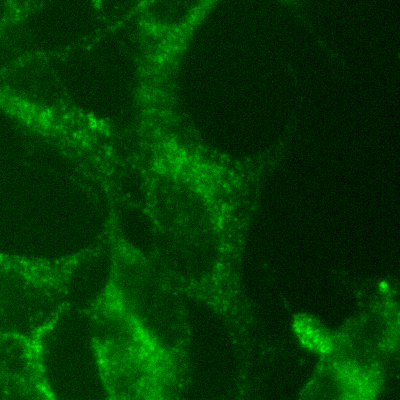

Supplement: Supplementary file 9 — Source data Fig. 7 [file 44321_2024_148_MOESM9_ESM.zip › Figure 7/C/WT ATG9A.png]

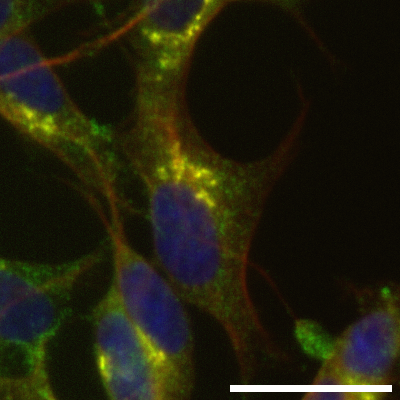

Supplement: Supplementary file 9 — Source data Fig. 7 [file 44321_2024_148_MOESM9_ESM.zip › Figure 7/C/WT merge(RGB).png]

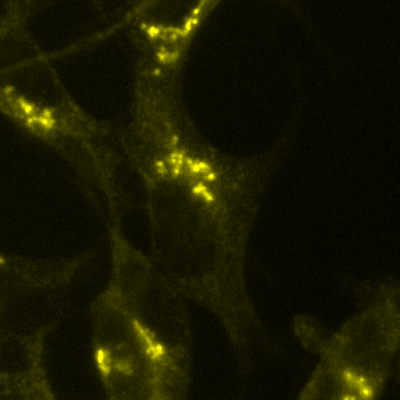

Supplement: Supplementary file 9 — Source data Fig. 7 [file 44321_2024_148_MOESM9_ESM.zip › Figure 7/C/WT TGN.png]
