# Supplementary material for: Pre-clinical development of AP4B1 gene replacement therapy for hereditary spastic paraplegia type 47
Source: EMBO Mol Med. 2024 Oct 2;16(11):2882–917. doi: 10.1038/s44321-024-00148-5 (PMC11554807; doi:10.1038/s44321-024-00148-5)
Supplement: Supplementary file 12 — Expanded View Figures [file 44321_2024_148_MOESM12_ESM.pdf]

Expanded View Figures

Figure EV1. SPG47 patients' fibroblasts show a rescue in ATG9A expression when treated with LV/hAP4B1.

(**A**) Patients' fibroblast stained with ATG9A (green) and TGN46 (red) show mislocalisation of ATG9A compared with healthy fibroblasts. SPG47 patient cells marked with white asterisks show rescue of mislocalised ATG9A after treatment with LV/V5-hAP4B1. Scale bar 20 µm (**B**) Representative western blot confirms expression of the hAP4B1 within the fibroblasts with increasing viral MOI. (**C**) Representative western blot shows the increase in ATG9A expression in KO fibroblasts ($p = 0.0005$) compared to WT and demonstrates the reduction in ATG9A expression when treated with increasing MOI of LV/hAP4B1 ($p = 0.0443$ for MOI 5, $p = 0.0014$ MOI 10, $p = 0.0011$ MOI 20). Corresponding quantification shows MOI 10 and 20 both rescue the ATG9A phenotype to healthy fibroblasts (control) levels. Data is presented as mean +/− standard error of the mean (SEM), $n = 3$ biological repeats. Data analysed by one-way ANOVA followed by post hoc Dunnett's multiple comparisons test with respect to Ctrl. Stars indicate $p \le 0.05$ (*); $p \le 0.01$ (**), $p \le 0.001$ (***); ns = not significant.

 © The Author(s)

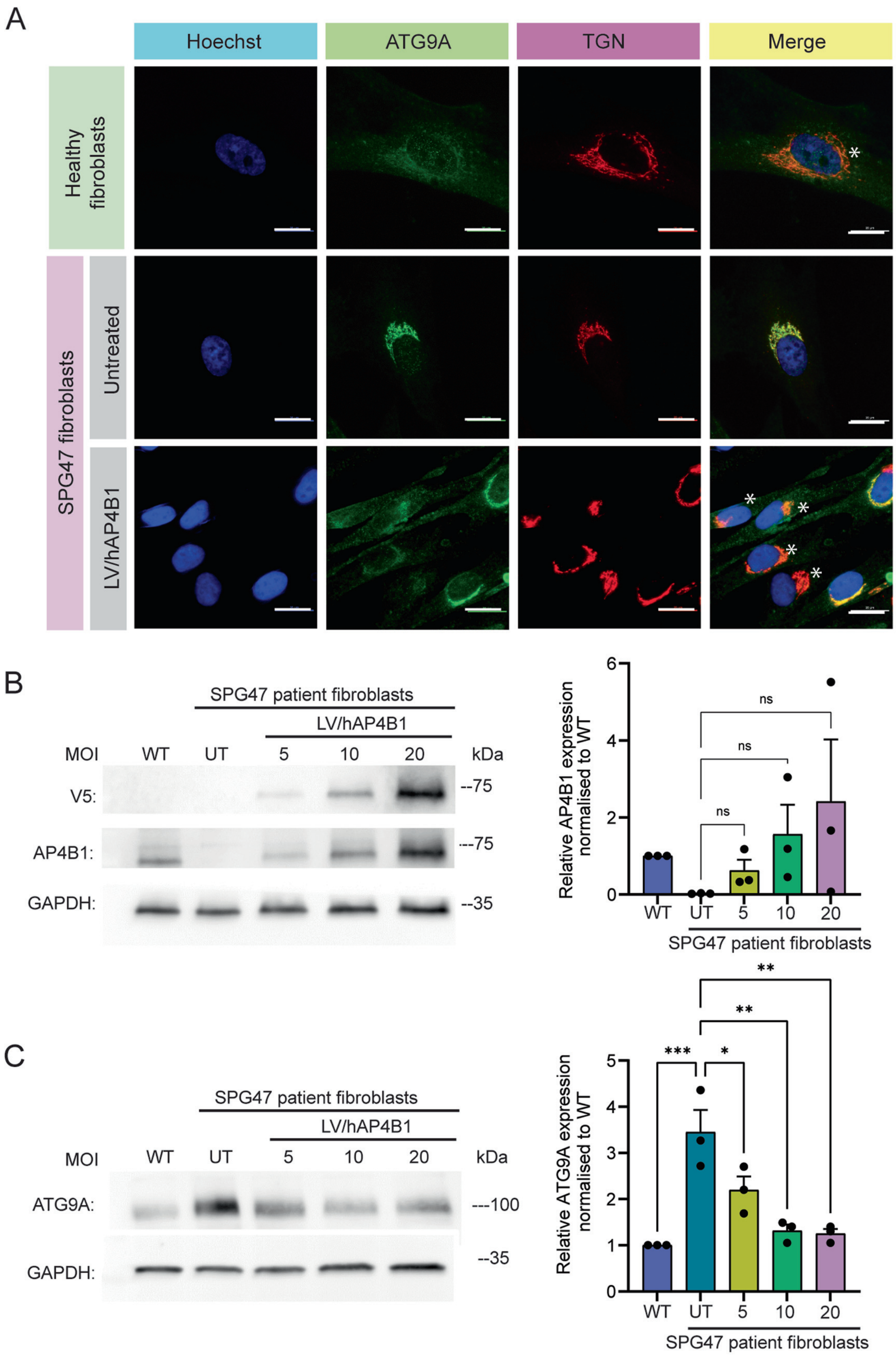

A
Hoechst
ATG9A
TGN
Merge
Healthy fibroblasts
SPG47 fibroblasts
Untreated
LV/hAP4B1
B
SPG47 patient fibroblasts
LV/hAP4B1
MOI WT UT 5 10 20 kDa
V5:
AP4B1:
GAPDH:
--75
---75
--35
Relative AP4B1 expression normalised to WT
ns
ns
ns
WT UT 5 10 20
SPG47 patient fibroblasts
C
SPG47 patient fibroblasts
LV/hAP4B1
MOI WT UT 5 10 20 kDa
ATG9A:
GAPDH:
---100
--35
Relative ATG9A expression normalised to WT
***
*
**
**
WT UT 5 10 20
SPG47 patient fibroblasts

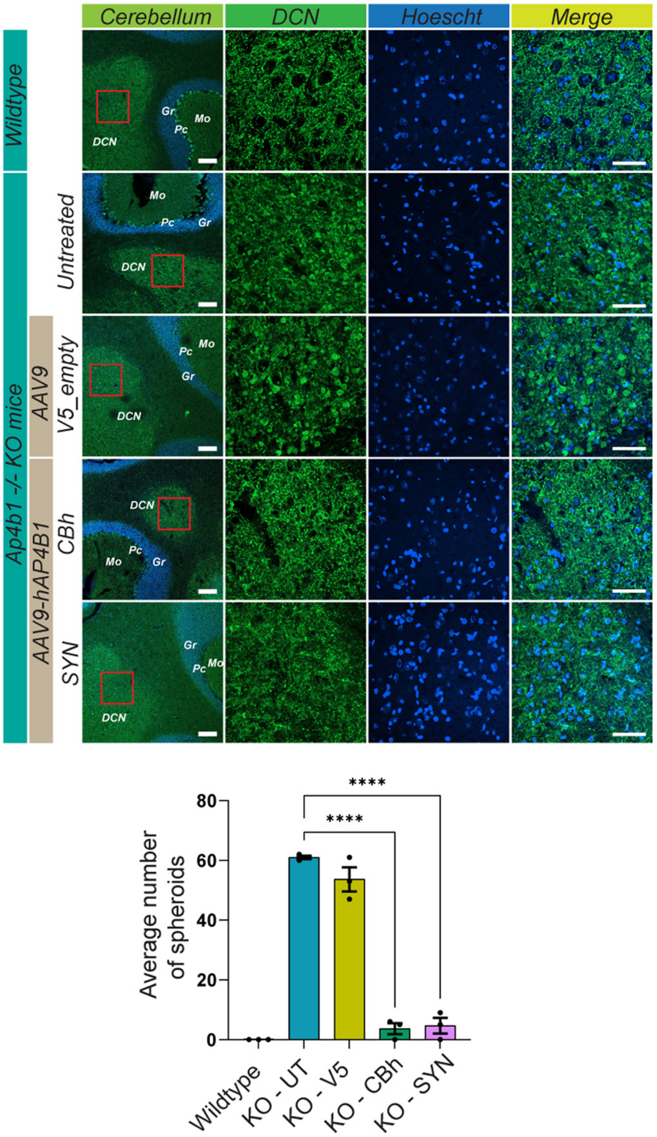

Figure EV2. Neonatal ICM treatment with AAV9/hAP4B1 in *Ap4b1*-KO mice rescue Calbindin-positive spheroids in the DCN 9 months following treatment.

The image panel depicts representative micrographs of the DCN within the cerebellum, stained with Calbindin (green) and Hoechst (blue). The first column shows low magnification images of the DCN and surrounding areas, with labels for the Molecular layer (Mo), Purkinje cell layer (Pc), Granular layer (Gr), and deep cerebellar nuclei (DCN). Scale bar 100 µm. A red box indicates the area of higher magnification within the DCN shown in the second column. The micrographs in the second column demonstrate a clear reduction of calbindin-positive spheroids with both AAV9-CBh-hAP4B1 and AAV9-SYN-hAP4B1 vectors. The third column shows nuclear staining with Hoechst, and the fourth column presents a merge of Calbindin and Hoechst stains. Scale bar 50 µm. The bar graph reveals a larger number of spheroids in untreated and control-treated Ap4b1 KO mice compared to no spheroids in the WT mice. These spheroids are significantly reduced with both CBh ($p < 0.0001$) and SYN ($p < 0.0001$) treatment vectors 9 months following treatment. Data are presented as mean ± standard error of the mean (SEM), with $n = 3$. The data were analysed using one-way ANOVA followed by Dunnett's multiple comparisons test. Stars indicate $p \leq 0.0001$ (****).

 © The Author(s)

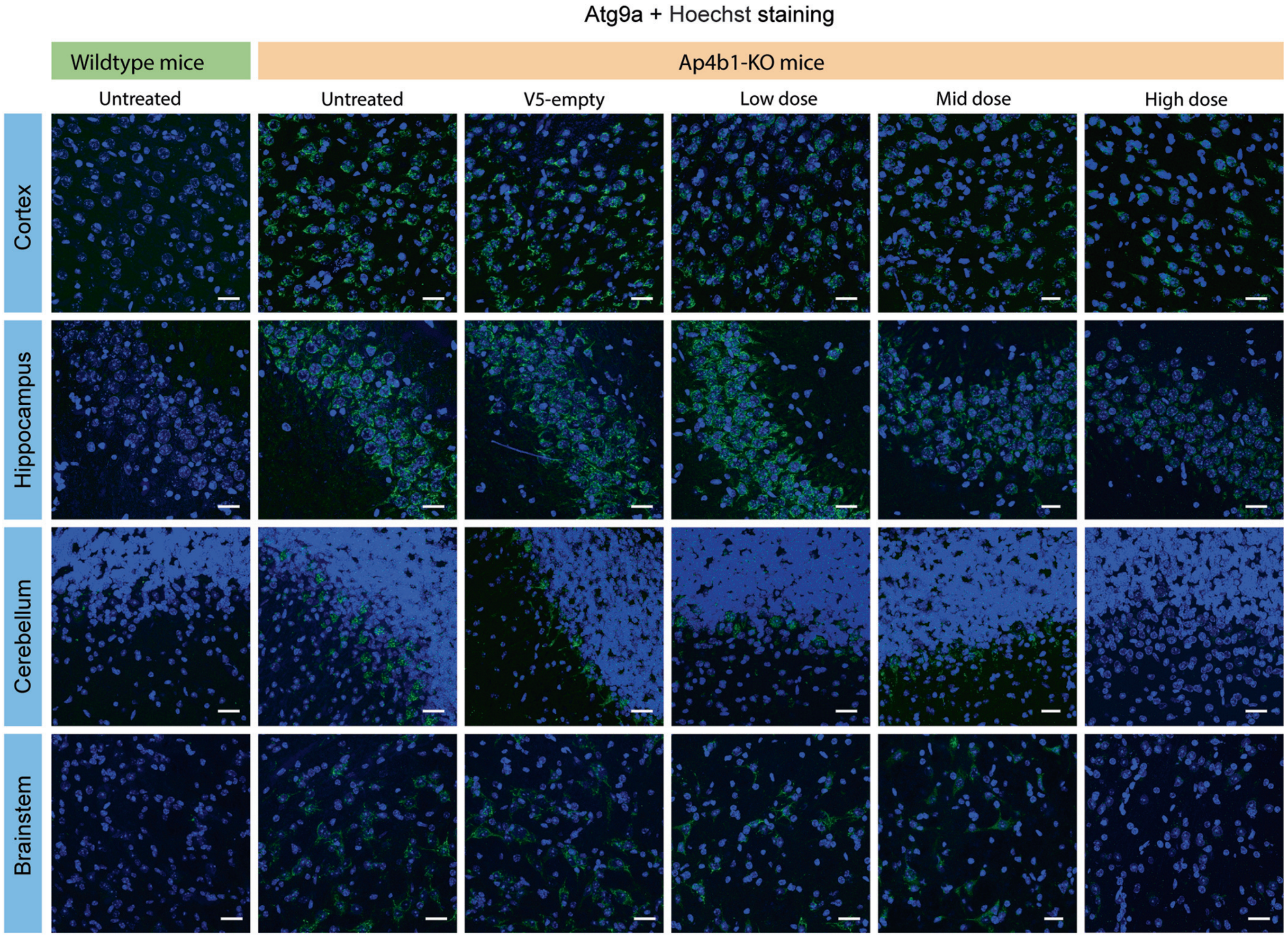

Figure EV3. Corresponding Hoechst staining to Fig. 6A to show the nuclear organisation within the separate brain regions.

Atg9a staining (green), Hoechst staining (blue). Representative micrographs showing a dose-dependent reduction of Atg9a perinuclear accumulation in the brain regions: cortex, hippocampus, cerebellum, brainstem. Scale bar 20 µm.

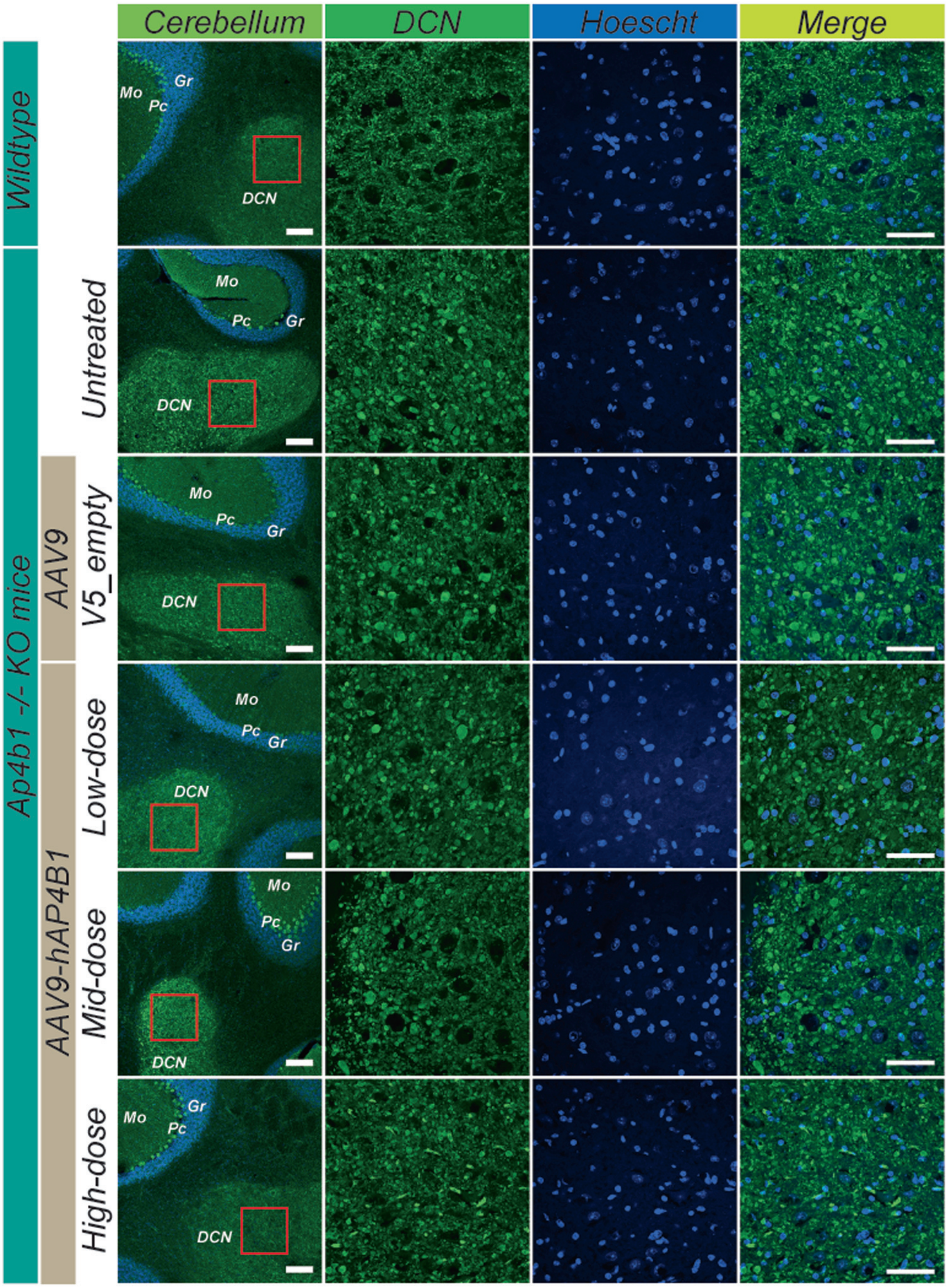

Cerebellum
DCN
Hoescht
Merge
Wildtype
Ap4b1 -/- KO mice
Untreated
AAV9
V5_empty
AAV9-hAP4B1
Low-dose
Mid-dose
High-dose
Mo
Pc
Gr
DCN

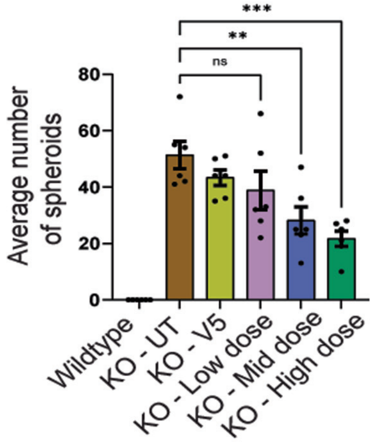

Average number of spheroids
80
60
40
20
0
***
**
ns
Wildtype
KO - UT
KO - V5
KO - Low dose
KO - Mid dose
KO - High dose

 © The Author(s)

Figure EV4. Adult ICM treatment with AAV9-CBh-hAP4B1 significantly reduces Calbindin-positive spheroids in the DCN of Ap4b1-KO mice 9 months following treatment.

The image panel depicts representative micrographs of the DCN within the cerebellum, stained with Calbindin (green) and Hoechst (blue). The first column shows low-magnification images of the DCN and surrounding areas, with labels for the Molecular layer (Mo), Purkinje cell layer (Pc), Granular layer (Gr), and deep cerebellar nuclei (DCN). Scale bar 100 µm. A red box indicates the area of higher magnification within the DCN shown in the second column. The micrographs in the second column demonstrate a clear dose-dependent reduction of calbindin-positive spheroids with increasing dose of the therapeutic vector (CBh-hAP4B1). The third column shows nuclear staining with Hoechst, and the fourth column presents a merge of Calbindin and Hoechst stains. Scale bar 50 µm. The bar graph reveals spheroids are reduced on a dose-dependent basis with mid- and high-dose significantly reducing the presence of spheroids ($p = 0.0066$ and $p = 0.0004$, respectively). Data are presented as mean ± standard error of the mean (SEM), with $n = 6$. The data were analysed using one-way ANOVA followed by Dunnett's multiple comparisons test. Stars indicate $p \leq 0.001$ (***), $p \leq 0.01$ (**), ns = not significant.

© The Author(s)

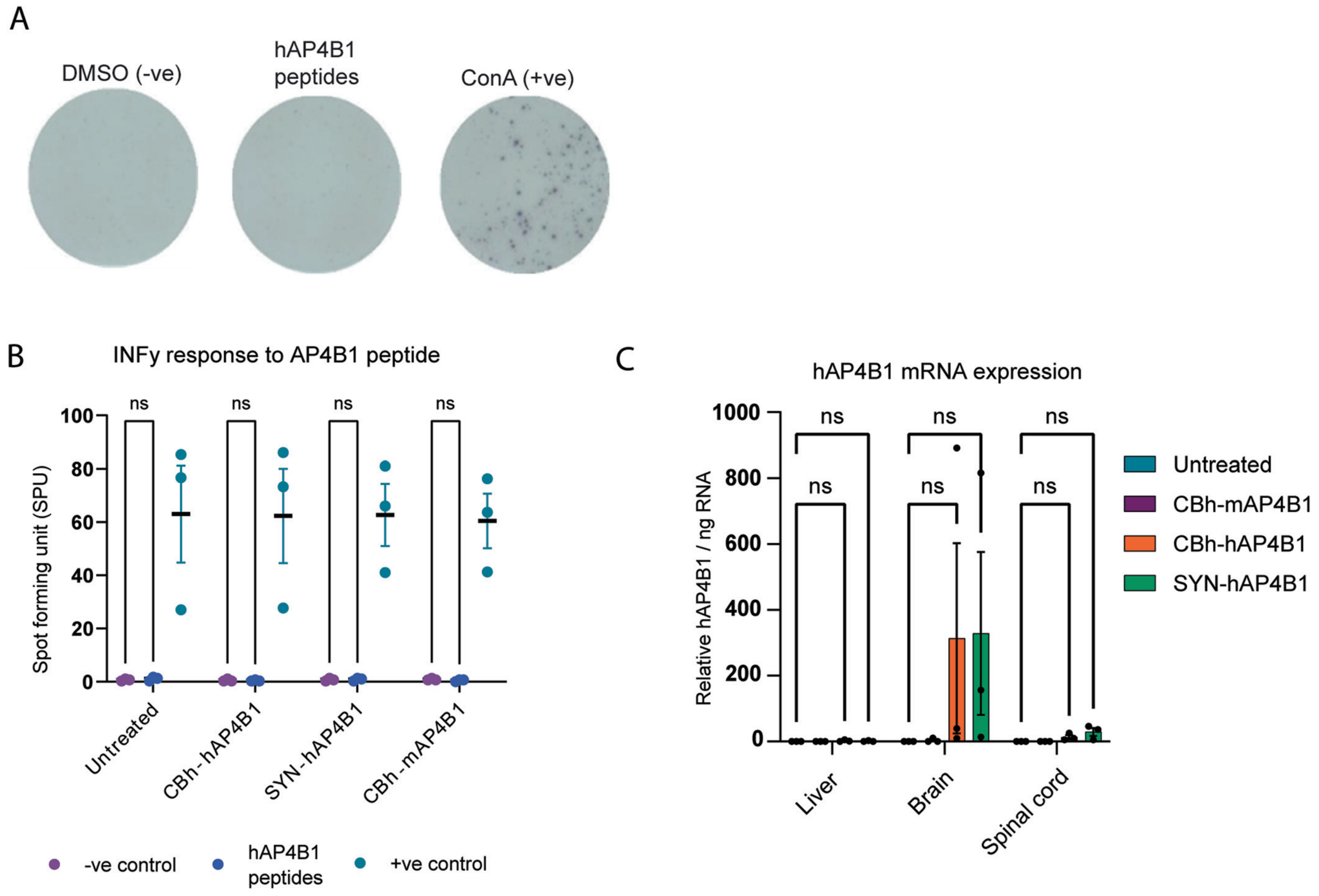

Figure EV5. In vivo safety study in WT mice through ELISpot assay, AAV9/hAP4B1 treatment generated no B cell response to the hAP4B1 peptides.

Vectors CBh-hAP4B1 and SYN-hAP4B1 were tested, alongside the vector containing the mouse *Ap4b1* gene (mAP4B1). Splenocytes that were prepared from WT mice following were assessed through an ELISpot assay for IFN-γ responses to AP4B1 peptides. (**A**) Representative images of the spot forming detection revealed following the exposure to the negative control (DMSO), the hAP4B1 peptides and the positive control (Concanavalin A). (**B**) Treated mice did not show any inflammatory response to the peptides. (**C**) RT-qPCR of total RNA extracted demonstrated elevated hAP4B1 mRNA expression in the brain, liver and spinal cord of treated WT mice with lower expression within the liver. Data are presented as mean ± SEM, $n = 3$ per group. Data are analysed by a two-way ANOVA with Tukey's post hoc multiple comparison test. ns = not significant.

 © The Author(s)
